# Eurotiumins A–E, Five New Alkaloids from the Marine-Derived Fungus *Eurotium* sp. SCSIO F452

**DOI:** 10.3390/md16040136

**Published:** 2018-04-21

**Authors:** Wei-Mao Zhong, Jun-Feng Wang, Xue-Feng Shi, Xiao-Yi Wei, Yu-Chan Chen, Qi Zeng, Yao Xiang, Xia-Yu Chen, Xin-Peng Tian, Zhi-Hui Xiao, Wei-Min Zhang, Fa-Zuo Wang, Si Zhang

**Affiliations:** 1 CAS Key Laboratory of Tropical Marine Bio-Resources and Ecology, RNAM Center for Marine Microbiology, Guangdong Key Laboratory of Marine Materia Medica, South China Sea Institute of Oceanology, Chinese Academy of Sciences, 164 West Xingang Road, Guangzhou 510301, China; wmzhong@scsio.ac.cn (W.-M.Z.); wangjunfeng@scsio.ac.cn (J.-F.W.); shixuefeng@scsio.ac.cn (X.-F.S.); 18489875310@163.com (Q.Z.); xy920412@sina.cn (Y.X.); xychen1994@gmail.com (X.-Y.C.); xinpengtian@scsio.ac.cn (X.-P.T.); xzh@scsio.ac.cn (Z.-H.X.); 2 University of Chinese Academy of Sciences, 19 Yuquan Road, Beijing 100049, China; 3 Key Laboratory of Plant Resources Conservation and Sustainable Utilization, South China Botanical Garden, Chinese Academy of Sciences, Guangzhou 510650, China; wxy@scbg.ac.cn; 4 State Key Laboratory of Applied Microbiology Southern China, Guangdong Provincial Key Laboratory of Microbial Culture Collection and Application, Guangdong Open Laboratory of Applied Microbiology, Guangdong Institute of Microbiology, 100 Central Xianlie Road, Guangzhou 510070, China; chenyc@gdim.cn (Y.-C.C.); wmzhang@gdim.cn (W.-M.Z.)

**Keywords:** prenylated indole alkaloid, benzyl pyrimidine, antioxidative and cytotoxic activities, marine fungi

## Abstract

Three new prenylated indole 2,5-diketopiperazine alkaloids (**1**–**3**) with nine known ones (**5**–**13**), one new indole alkaloid (**4**), and one new bis-benzyl pyrimidine derivative (**14**) were isolated and characterized from the marine-derived fungus *Eurotium* sp. SCSIO F452. **1** and **2**, occurring as a pair of diastereomers, both presented a hexahydropyrrolo[2,3-*b*]indole skeleton. Their chemical structures, including absolute configurations, were elucidated by 1D and 2D NMR, HRESIMS, quantum chemical calculations of electronic circular dichroism, and single crystal X-ray diffraction experiments. Most isolated compounds were screened for antioxidative potency. Compounds **3**, **5**, **6**, **7**, **9**, **10**, and **12** showed significant radical scavenging activities against DPPH with IC_50_ values of 13, 19, 4, 3, 24, 13, and 18 µM, respectively. Five new compounds were evaluated for cytotoxic activities.

## 1. Introduction

Prenylated indole 2,5-diketopiperazines (DKPs) constitute a significant class of densely functionalized structures characterized by condensation of two α-amino acids, generally featuring a reverse prenyl moiety at C-2 of the indole nucleus. They are widely distributed in fungi, especially in the genera *Aspergillus*, *Penicillium*, and *Eurotium* [1,2,3]. Interest in indole 2,5-DKPs is due to their significant biological activities, such as antibacterial [4], antiviral [5], anticancer [6], immunomodulatory [7], and α-glucosidase inhibitory activities [8]. Specifically, Plinabulin, a synthetic analog of the natural DKP product halimide from marine-derived *Aspergillus* sp. CNC-139, has entered phase-III clinical study for the treatment of non-small cell lung cancer [9,10]. In the course of our continuing investigation on marine fungi [11,12], a further chemical investigation was performed on *Eurotium* sp. SCSIO F452, which was isolated from a South China Sea sediment sample and found to be producing DKPs [13]. Careful chemical examination led to the isolation and characterization of three new DKPs (**1**–**3**) along with nine known ones (**5**–**13**), one new prenylated indole alkaloid (**4**), and one new bis-benzyl pyrimidine derivative (**14**). Herein, we report the isolation, structure elucidation, antioxidative, and cytotoxic activities of these compounds.

## 2. Results

**Figure 1:**
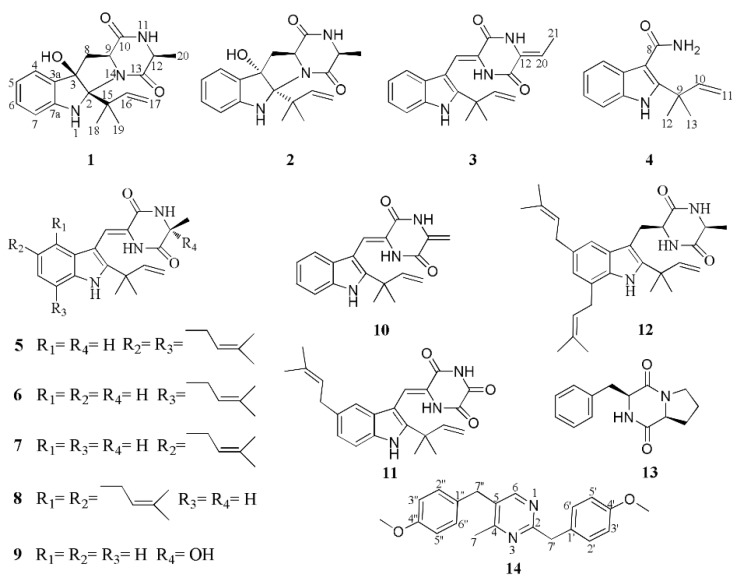
Chemical structures of compounds **1**–**14**.

A 30 L fermentation broth of marine-derived fungus *Eurotium* sp. SCSIO F452 was extracted three times with ethyl acetate at room temperature. The solvent was evaporated in vacuo to produce a crude extract. Subsequent fractionation by various chromatographic methods including column chromatography (CC) over silica gel, octadecylsilyl silica gel (ODS), and high performance liquid chromatography (HPLC) yielded compounds **1**–**14** (Figure 1).

**Figure 2:**
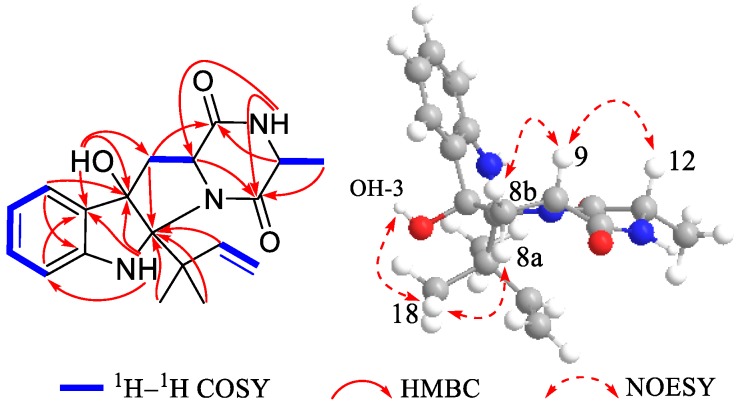
Key ^1^H-^1^H COSY, HMBC, and NOESY correlations of compound **1**.

**Figure 3:**
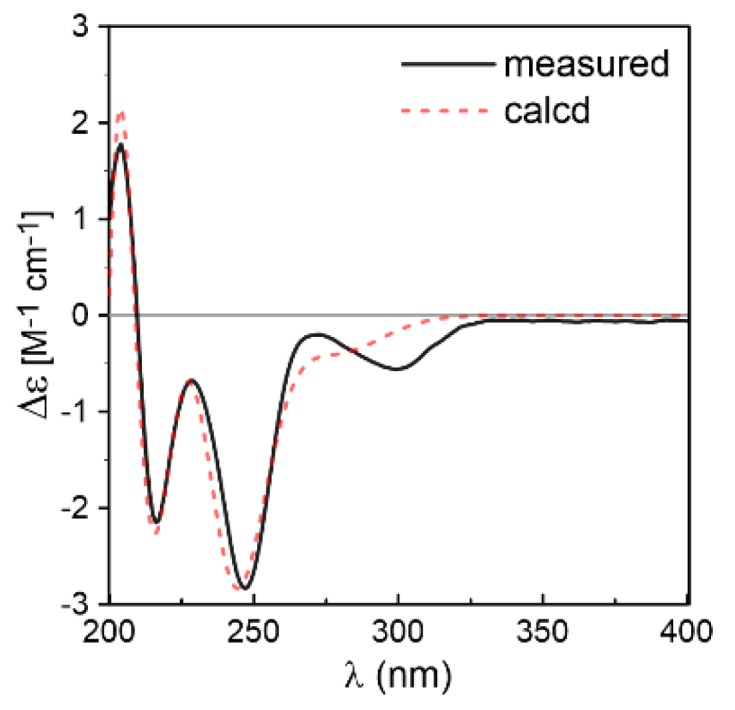
Comparison between the experimental and the CAM-B3LYP/TZVP/PCM (MeOH) calculated ECD spectra of **1**.

**Figure 4:**
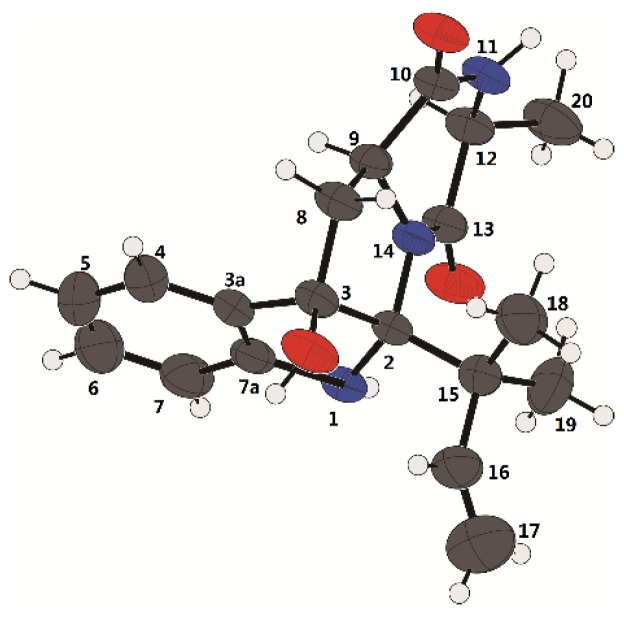
X-ray crystallographic structure of compound **1** (Black: carbon atom; gray: hydrogen atom; red: oxygen atom; blue: nitrogen atom).

Compound **1** was isolated as white crystals. Its molecular formula was determined as C_19_H_23_N_3_O_3_ by the positive HRESIMS (*m/z* 342.1816 [M + H]^+^, calcd for 342.1812), indicating an index of hydrogen deficiency of 10. Its IR spectrum suggested the presence of hydroxyl and amine groups (3366, 3312 cm^−1^) and carbonyl functionalities (1684, 1668 cm^−1^). The ^1^H NMR (Table 1) spectrum of **1** recorded in acetone-*d*_6_ showed three methyls at *δ*_H_ 1.27 (s), 1.29 (s), 1.33 (d, *J* = 6.9 Hz), one aliphatic methylene at *δ*_H_ 2.56 (dd, *J* = 12.7, 7.6 Hz) and 2.76 (dd, *J* = 12.7, 11.2 Hz), two aliphatic methines at *δ*_H_ 3.73 (dd, *J* = 11.2, 7.6 Hz) and *δ*_H_ 3.91 (q, *J* = 6.9 Hz), four aromatic protons at *δ*_H_ 6.75 (overlap), 6.76 (overlap), 7.12 (td, *J* = 7.6, 1.2 Hz), 7.28 (d, *J* = 7.6 Hz), three exchangeable protons at *δ*_H_ 4.49 (s), 6.53 (s), 7.16 (br s), and three olefinic proton resonances at *δ*_H_ 4.90 (dd, *J* = 10.9, 1.5 Hz), 4.98 (dd, *J* = 17.7, 1.2 Hz), 6.36 (dd, *J* = 17.6, 10.9 Hz) suggesting the existence of a monosubstituted double bond. The ^13^C NMR (Table 1) and DEPT revealed the presence of 19 carbon resonances, including three methyls, two methylenes (one olefinic carbon), seven methines (five olefinic carbons), and seven quaternary carbons (one oxygenated carbon, one nitrogenated carbon, two amide groups, two olefinic carbons, and one quaternary carbon). By the heteronuclear single quantum coherence (HSQC) spectrum, all proton resonances were unambiguously assigned to their respective carbons except for the exchangeable protons. All the above data suggested that **1** possessed a hexahydropyrrolo[2,3-*b*]indole skeleton, showing high similarities with *cyclo*-C3*β*-dimethylallyl-l-Trp-l-Ala [14], except that the dimethylallyl group was substituted at C-2 and a hydroxyl group was located at C-3 in **1**. This was supported by the heteronuclear multiple bond correlation (HMBC) correlations (Figure 2) from H-16 (*δ*_H_ 6.36), H_3_-18 (*δ*_H_ 1.27), and H_3_-19 (*δ*_H_ 1.29) to C-2 (*δ*_C_ 92.4), and from OH-3 (*δ*_H_ 4.49 s) to C-3 (*δ*_C_ 89.4), C-3a (*δ*_C_ 132.2), and C-8 (*δ*_C_ 36.4). The relative configuration of **1** was determined by nuclear Overhauser effect spectroscopy (NOESY) correlations (Figure 2). The NOE correlations of H-9 (*δ*_H_ 3.73) with H-8b (*δ*_H_ 2.56) and H-12 (*δ*_H_ 3.91) indicated that they were located at the same side as α-orientation. Furthermore, the NOE correlations of H_3_-18 and H_3_-19 with OH-3 and H-8a (*δ*_H_ 2.76) led to their cofacial assignment as *β*-orientation. On the basis of the above relative configuration analysis, **1** had only one pair of enantiomers (2*S*,3*R*,9*S*,12*S*-**1** and 2*R*,3*S*,9*R*,12*R*-**1**). The quantum chemical calculation study for electronic circular dichroism (ECD) was conducted to determine its absolute configuration using time-dependent density-functional theory (TDDFT) method. Consequently, the calculated ECD for 2*S*,3*R*,9*S*,12*S*-**1** showed good agreement with the experimental ECD of **1** (Figure 3), which allowed the assignment of the absolute configuration of **1** as 2*S*,3*R*,9*S*,12*S.* Delightedly, a single crystal of **1** suitable for X-ray diffraction (Figure 4) was obtained from methanol. The final refinement on the Cu Kα data with a good Flack parameter 0.04(6) unambiguously confirming the absolute stereochemistry to be 2*S*,3*R*,9*S*,12*S*, which was consistent with the calculated ECD result. Thus, the structure of **1** was assigned as 2*S*,3*R*,9*S*,12*S*-*cyclo*-2-dimethylallyl-3-hydroxy-l-Trp-l-Ala (Figure 1) and named eurotiumin A.

**Figure 5:**
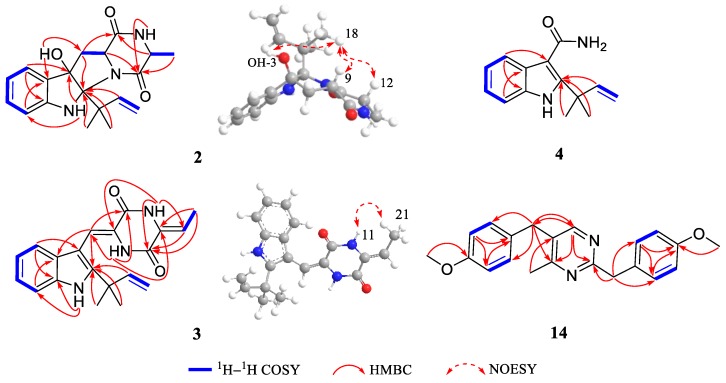
Key ^1^H-^1^H COSY, HMBC and NOESY correlations of compounds **2**–**4** and **14**.

**Figure 6:**
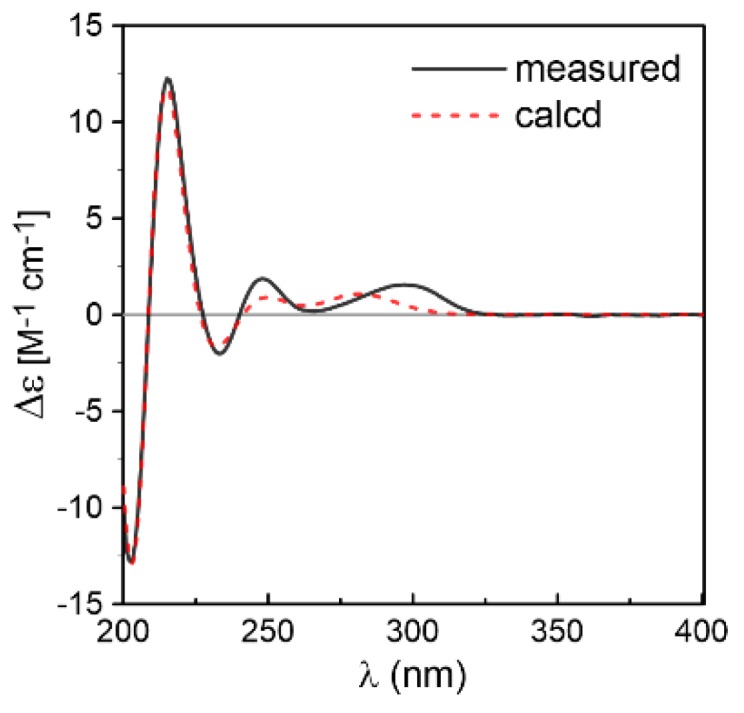
Comparison between the experimental and the CAM-B3LYP/TZVP/PCM (MeOH) calculated ECD spectra of **2**.

Compound **2** was obtained as white solid. Its molecular formula C_19_H_23_N_3_O_3_ was established by positive HRESIMS at *m/z* 342.1813 [M + H]^+^ (calcd for 342.1812), which was identical to that of **1**. The UV, IR, and 1D NMR spectra (Table 1) of **2** highly resembled those of **1**, indicating **2** also possessed a hexahydropyrrolo[2,3-*b*]indole scaffold. Detailed analysis of its 2D NMR (Figure 5) data indicated that **2** and **1** were represented as a pair of diastereomers. This conclusion could be made by the key HMBC (Figure 5) correlations from H-16 (*δ*_H_ 6.52, dd, *J* = 17.6, 10.8 Hz), H_3_-18 (*δ*_H_ 1.36, s), and H_3_-19 (*δ*_H_ 1.39, s) to C-2 (*δ*_C_ 95.4), and from OH-3 (*δ*_H_ 4.32, s) to C-3 (*δ*_C_ 88.3), C-3a (*δ*_C_ 132.7), and C-8 (*δ*_C_ 36.8), and NOESY (Figure 5) correlations of H_3_-18 and H_3_-19 with H-9 (*δ*_H_ 4.41, dd, *J* = 10.9, 2.5 Hz), H-12 (*δ*_H_ 4.12, q, *J* = 6.7 Hz), and OH-3, indicating that **2** was a C-2 and C-3 isomer of **1.** Since **1** was confirmed to be condensed by two l-amino acids, the stereocenters at C-9 and C-12 of **2** tentatively had the same 9*S*,12*S* configuration as **1** in view of the same biogenetic pathway. Cumulatively, the absolute configuration of **2** was assigned as 2*R*,3*S*,9*S*,12*S*. Furthermore, the calculated ECD curve for 2*R*,3*S*,9*S*,12*S*-**2** displayed good agreement with the experimental ECD of **2** (Figure 6), which confirmed the above elucidation. Thus, the structure of **2** was assigned as 2*R*,3*S*,9*S*,12*S*-*cyclo*-2-dimethylallyl-3-hydroxy-l-Trp-l-Ala (Figure 1) and named eurotiumin B.

Compound **3** was isolated as yellow oil. The molecular formula C_20_H_21_N_3_O_2_ was determined on the basis of negative HRESIMS at *m/z* 334.1567 [M − H]^−^ (calcd for 334.1561), corresponding to an index of hydrogen deficiency of 12. Its IR spectrum suggested the presence of amine groups (3363, 3197 cm^−1^) and carbonyl functionalities (1678, 1647 cm^−1^). Its ^1^H NMR (Table 1) spectrum recorded in dimethyl sulfoxide-*d*_6_ showed four aromatic protons at *δ*_H_ 7.02 (td, *J* = 7.5, 1.0 Hz), 7.18 (d, *J* = 7.9 Hz), 7.10 (td, *J* = 7.6, 1.1 Hz), and 7.43 (d, *J* = 8.1 Hz), three NH signals at *δ*_H_ 8.68 (s), 10.21 (s), and 11.12 (s), in combination with four aromatic methines at *δ*_C_ 111.7, 118.8, 119.6, and 120.9, four quaternary carbons at *δ*_C_ 103.3, 125.9, 135.1, and 144.3, together with two amides at *δ*_C_ 156.4 and 157.6 in the ^13^C NMR (Table 1) spectrum, suggesting a 2,3-disubstituted indole DKP substructure [3]. Careful comparison of its 1D and 2D NMR (Figure 5) data with neoechinulin B (**10**) [15] showed high similarities except for that an olefinic methylene (*δ*_H_ 5.02, s; 5.36, s; *δ*_C_ 100.0) in neoechinulin B was transformed into an olefinic methine (*δ*_H_ 5.87, q, *J* = 7.6 Hz; *δ*_C_ 112.9) substituted by a doublet methyl (*δ*_H_ 1.81, d, *J* = 7.6 Hz; *δ*_C_ 11.1) in **3**. The above conclusion was verified by the key ^1^H-^1^H correlation spectroscopy (^1^H-^1^H COSY) cross peak of H-20/H_3_-21 and HMBC correlations from NH-11 (*δ*_H_ 10.21, s) to C-9 (*δ*_C_ 124.5), C-12 (*δ*_C_ 128.4), and C-13 (*δ*_C_ 156.4), from H-20 (*δ*_H_ 5.87) to C-13 and C-21 (*δ*_C_ 11.1), and from H_3_-21 (*δ*_H_ 1.81) to C-12, C-13 and C-20 (*δ*_C_ 112.9). The geometry of the Δ^8^ double bond was elucidated to be *Z* configuration by the downfield shift of H-8 (*δ*_H_ 6.94, s) due to the deshielding effect of the carbonyl group on the *β*-vinyl proton, which was also coincident with the lack of NOE effect between H-8 and NH-14 [16]. The newly generated Δ^12^ double bond in **3** was elucidated as *Z* geometry by the key NOESY (Figure 5) cross peak of H_3_-21 with NH-11. Thus the gross structure of **3** was depicted as shown in Figure 1 and named eurotiumin C.

Compound **4** was isolated as yellow solid. The molecular formula C_14_H_16_N_2_O was determined on the basis of positive HRESIMS at *m/z* 251.1159 [M + Na]^+^ (calcd for 251.1155), corresponding to an index of hydrogen deficiency of 8. Its IR spectrum suggested the presence of amine groups (3312, 3273 cm^−1^) and carbonyl functionality (1645 cm^−1^). Its ^1^H NMR (Table 2) spectrum recorded in acetone-*d*_6_ showed two methyls both at *δ*_H_ 1.64 (s), one monosubstituted double bond at *δ*_H_ 5.06 (dd, *J* = 10.6, 1.2 Hz), 5.12 (dd, *J* = 17.5, 1.2 Hz), 6.41 (dd, *J* = 17.5, 10.6 Hz), four aromatic protons at *δ*_H_ 7.04 (td, *J* = 7.1, 1.2 Hz), 7.07 (td, *J* = 7.0, 1.3 Hz), 7.37 (d, *J* = 8.1 Hz), and 7.70 (d, *J* = 7.7 Hz), combined with four aromatic methines at *δ*_C_ 112.0, 120.2, 120.8, and 122.1, four quaternary carbons at *δ*_C_ 109.8, 128.5, 135.1, and 146.1 in the ^13^C NMR (Table 2) spectrum, implying a prenylated indole alkaloid substructure. Its 1D and 2D (Figure 5) NMR data highly resembled that of 2-(2-methyl-3-en-2-yl)-1*H*-indole-3-carbaldehyde, which was isolated from this fungus previously [13]. The only difference was that the aldehyde group at C-3 was replaced by an amide group, which could be deduced by the HRESIMS data and upfield of C-8 chemical shift from *δ*_C_ 186.6 in 2-(2-methyl-3-en-2-yl)-1*H*-indole-3-carbaldehyde to *δ*_C_ 169.1 in **4**. Thus, the structure of **4** was assigned as 2-(2-methyl-3-en-2-yl)-1*H*-indole-3-carboxamide (Figure 1) and named eurotiumin D.

Compound **14** was isolated as yellow solid. Its positive HRESIMS at *m/z* 335.1764 [M + H]^+^ (calcd for 335.1754) revealed a molecular formula of C_21_H_22_N_2_O_2_ with an index of hydrogen deficiency of 12. Its ^1^H NMR (Table 2) spectrum recorded in acetone-*d*_6_ showed one methyl at *δ*_H_ 2.44 (s), two methoxyls at *δ*_H_ 3.73 (s), 3.75 (s), two aliphatic methylenes at *δ*_H_ 4.01 (s), 4.08 (s), nine aromatic protons at *δ*_H_ 6.82 (d, *J* = 8.6 Hz), 6.85 (d, *J* = 8.6 Hz), 7.12 (d, *J* = 8.7 Hz), 7.23 (d, *J* = 8.7 Hz), and 8.26 (s). The ^13^C NMR and DEPT revealed 21 carbon resonances, including one methyl, two methoxyls, two methylenes, nine methines, and seven quaternary carbons. Carefully analysis of its 1D (Table 2) and 2D NMR (Figure 5) data showed two 4-methoxybenzyl moieties and one 4-methylpyrimidine unit. The two 4-methoxybenzyl moieties could be judged by the ^1^H NMR resonances of two AA′BB′ patterns for protons at H-2′/H-6′ (*δ*_H_ 7.23, d, *J* = 8.7 Hz), H-3′/H-5′ (*δ*_H_ 6.85, d, *J* = 8.6 Hz), H-2″/H-6″ (*δ*_H_ 7.12, d, *J* = 8.7 Hz), and H-3″/H-5″ (*δ*_H_ 6.82, d, *J* = 8.7 Hz), and ^1^H-^1^H COSY correlations of H-2′/H-3′, H-5′/H-6′, H-2″/H-3″, H-5″/H-6″, and HMBC correlations from H-7′ (*δ*_H_ 4.08, s) to C-1′ (*δ*_C_ 132.2), C-2′/C-6′ (*δ*_C_ 130.8), from H-7″ (*δ*_H_ 4.01, s) to C-1″ (*δ*_C_ 131.3), C-2″/ C-6″ (*δ*_C_ 130.5), from 4′-OMe (*δ*_H_ 3.75, s) to C-4′ (*δ*_C_ 159.3), and from 4″-OMe (*δ*_H_ 3.73, s) to C-4″ (*δ*_C_ 159.3). The 4-methylpyrimidine part could be elucidated by the HRESIMS data and HMBC correlations from H-6 (*δ*_H_ 8.26, s) to C-2 (*δ*_C_ 152.9), C-4 (*δ*_C_ 154.4), C-7″ (*δ*_C_ 40.7), and from H_3_-7 (*δ*_H_ 2.44, s) to C-5 (*δ*_C_ 152.2). The two 4-methoxybenzyl moieties were located at C-2 and C-5 of 4-methylpyrimidine, respectively, which was evidenced by the HMBC correlations from H-7′ to C-2, from H-7″ to C-4, C-6. Thus, the structure of **14** was established as 2,5-bis(4-methoxybenzyl)-4-methylpyrimidine (Figure 1) and named eurotiumin E. Benzyl pyrimidines are prominent heterocyclic compounds with significant pharmaceutical activities, such as inhibiting HIV-1 reverse transcriptase [17], binding specifically to human serum albumin and determining the drug distribution [18], and acting against bacterial and avian dihydrofolate reductase [19]. To the best of our knowledge, benzyl pyrimidines were reported mainly as chemical synthetic products [17,20,21], but rarely as natural products. This investigation could shed light on the further discovery of benzyl pyrimidines from marine fungi.

In addition to the isolation of the above new compounds **1**–**4** and **14**, nine known DKPs including dehydroechinulin (**5**) [22], variecolorin G (**6**) [5], isoechinulin A (**7**) [5], dehydrovariecolorin L (**8**) [22], variecolorin O (**9**) [23], neoechinulin B (**10**) [15], variecolorin J (**11**) [5], echinulin (**12**) [22], and *cyclo*-(l-Pro-l-Phe) (**13**) [24] were also isolated and identified from this fungus. Their structures (Figure 1) were elucidated by comparison of their NMR and MS data with reported literature.

Prenylated DKP alkaloids had been reported exhibiting antioxidative and cytotoxic activities [1,2,3,6], thus all the compounds except **8** and **11**, due to the sample quantity limitation, were screened for antioxidative activities against DPPH [25] (Table 3). Compounds **3**, **5**, **6**, **7**, **9**, **10**, and **12** showed significant radical scavenging activities against DPPH with IC_50_ values of 13, 19, 4, 3, 24, 13, and 18 µM, respectively, which were comparable to that of the positive control ascorbic acid (Vc) (23 µM). Compounds **1**, **2**, and **13** exhibited moderate antioxidative activities with IC_50_ values ranging from 35 to 69 µM. Based on the antioxidative activities results, diprenylated analogs (**6** and **7**) seemed to be more active than monoprenylated ones (**1**–**3**, **9**, and **10**), and triprenylated ones (**5** and **12**). Particularly, as for **1** and **2**, the absolute configurations of the C-2 and C-3 appeared to have influence on the antioxidative activities. The new compounds **1**–**4**, and **14** were evaluated for their cytotoxic activities against SF-268 and HepG2 cell lines in vitro with the SRB method [26]. The results showed that none of them exhibited obvious cytotoxicity against these two cancer cell lines (IC_50_ > 100 µM).

## 3. Materials and Methods 

### 3.1. General Experimental Procedures

Optical rotations were measured with an MCP 500 automatic polarimeter (Anton Paar, Graz, Austria) with MeCN as solvent. UV spectra were recorded on a UV-2600 spectrometer (Shimadzu, Tokyo, Japan). IR spectra were measured on an IR Affinity-1 spectrometer (Shimadzu). ^1^H, ^13^C NMR, DEPT and 2D NMR spectra were recorded on the AVANCE III HD 700 (Bruker, Billerica, MA, USA). Circular dichroism spectra were measured with a Chirascan circular dichroism spectrometer (Applied Photophysics, Surrey, UK). HRESIMS spectra data were recorded on a MaXis quadrupole-time-of-flight mass spectrometer. Crystallographic data was collected on a Rigaku XtaLAB AFC12 single-crystal diffractometer using Cu Kα radiation. Thin layer chromatography (TLC) was performed on plates precoated with silica gel GF_254_ (10–40 µm). Column chromatography (CC) was performed over silica gel (200–300 mesh and 300–400 mesh) (Qingdao Marine Chemical Factory, Qingdao, China) and ODS (50 μm, YMC, Kyoto, Japan). High performance liquid chromatography was performed on an Agilent 1260 HPLC equipped with a DAD detector, using an ODS column (YMC-pack ODS-A, 10 × 250 mm, 5 µm, 3 mL/min). All solvents used in CC and HPLC were of analytical grade (Tianjin Damao Chemical Plant, Tianjin, China) and chromatographic grade (Oceanpak, Goteborg, Sweden), respectively. Fractions were monitored by TLC and spots were visualized by heating silica gel plates sprayed with 10% H_2_SO_4_ in EtOH.

### 3.2. Fungal Material

The fungal strain used in this investigation was isolated from a South China Sea sediment sample (17°29.804′ N, 110°0.292′ E) at a depth of 158 m in May 2010. It was identified as *Eurotium* sp. SCSIO F452, according to a molecular biological protocol by DNA amplification and sequencing of the ITS region (deposited in GenBank, accession no. JX481973). The working strain was prepared on potato dextrose agar slants modified with seawater instead of distilled water and stored at 4 °C. A reference culture was maintained at −80 °C in RNAM Center for Marine Microbiology, South China Sea Institute of Oceanology, Chinese Academy of Sciences.

### 3.3. Fermentation, Extraction, and Isolation

The strain *Eurotium* sp. SCSIO F452 was cultured under static conditions at 28 °C in 500 mL × 200 conical flasks containing the liquid medium (150 mL/flask) composed of maltose (20 g/L), glucose (10 g/L), mannitol (20 g/L), corn syrup (1 g/L), sodium glutamate (10 g/L), yeast extract (3 g/L), KH_2_PO_4_ (0.5 g/L), MgSO_4_·7H_2_O (0.3 g/L), CaCO_3_ (2 g/L), sea salt (30 g/L) (adjusted pH to 6.5 before sterilization). The fermented whole broth (30 L) was filtered through cheesecloth to separate into filtrate and mycelia. The filtrate was concentrated under vacuum to about a quarter of the original volume and then extracted three times with EtOAc, while the mycelia were extracted three times with 80% Acetone/H_2_O. The acetone solution was evaporated under reduced pressure to afford an aqueous solution. The aqueous solution was extracted three times with EtOAc to give another EtOAc solution. Both EtOAc solutions were combined and concentrated under reduced pressure to give the whole crude extract (68 g).

### 3.4. Purification

The EtOAc extract (68 g) was subjected to vacuum liquid chromatography (VLC) on a silica gel column using step gradient elution with petroleum ether (PE)/EtOAc (1:0 to 0:1) and CHCl_3_/MeOH (1:0 to 0:1) to separate into eighteen fractions based on TLC properties. Fr.4 (13 g) was separated by silica gel CC (PE/Acetone 1:0 to 0:1) to obtain nine subfractions (Frs.4.1–4.9). Then Fr.4.7 (0.9 g) was divided into ten parts (Frs.4.7.1–4.7.10) by ODS CC with a gradient elution of MeOH/H_2_O (7:3 to 1:0). Fr.4.7.1 (156 mg) was further purified by HPLC (55% CH_3_OH/H_2_O) to yield **1** (3.5 mg), **2** (2.0 mg), **4** (4.1 mg). Fr.4.7.2 (116 mg) was purified by HPLC (73% CH_3_CN/H_2_O) to yield **5** (1.0 mg), **10** (9.5 mg), **13** (24.5 mg). Fr.4.7.3 (22 mg) was purified by HPLC (72% CH_3_CN/H_2_O) to yield **6** (3.1 mg). Fr.4.6 (322 mg) was purified by repeated HPLC (65% CH_3_OH/H_2_O) to yield **7** (7.0 mg), **11** (0.8 mg), **12** (48.2 mg). Fr.3 (9.2 g) was separated by silica gel CC (PE/Acetone 1:0 to 0:1) to obtain ten subfractions (Frs.3.1–3.10). Then Fr.3.8 (162 mg) was further purified by HPLC (51% CH_3_CN/H_2_O) to yield **3** (1.5 mg), **8** (0.7 mg). Fr.3.9 (162 mg) was purified by HPLC (45% CH_3_CN/H_2_O) to yield **9** (5.1 mg), **14** (1.1 mg). 

### 3.5. Spectral Data

Eurotiumin A (**1**): white crystals; [α${]}_{D}^{25}$ = −120.9 (c 0.1, CH_3_CN); UV (CH_3_CN) *λ*_max_ (log *ε*) 205 (4.02), 241 (3.54), 295 (3.02) nm; IR (film) *v*_max_ 3366, 3312, 2926, 1684, 1668, 1364, 1151, 750 cm^−1^; HRESIMS at *m/z* 342.1816 [M + H]^+^ (calcd for 342.1812). ^1^H and ^13^C NMR see Table 1. 

Eurotiumin B (**2**): white solid; [α${]}_{D}^{25}$ = +76.5 (c 0.067, CH_3_CN); UV (CH_3_CN) *λ*_max_ (log *ε*) 204 (4.41), 237 (3.96), 296 (3.24) nm; IR (film) *v*_max_ 3366, 3273, 2924, 1683, 1653, 1472, 1379,1092, 750 cm^−1^; HRESIMS at *m/z* 342.1813 [M + H]^+^ (calcd for 342.1812). ^1^H and ^13^C NMR see Table 1.

Eurotiumin C (**3**): yellow oil; [α${]}_{D}^{25}$ = +2.8 (c 0.01, CH_3_CN); UV (CH_3_CN) *λ*_max_ (log *ε*) 226 (4.23), 272 (4.03), 353 (3.86) nm; IR (film) *v*_max_ 3364, 3292, 2924, 1678, 1647, 1396, 1371, 744 cm^−1^; HRESIMS at *m/z* 334.1567 [M − H]^−^ (calcd for 334.1561). ^1^H and ^13^C NMR see Table 1.

Eurotiumin D (**4**): yellow solid; [α${]}_{D}^{25}$ = −2.7 (c 0.1, CH_3_CN); UV (CH_3_CN) *λ*_max_ (log *ε*) 219 (4.05), 281 (3.40), 288 (3.35) nm; IR (film) *v*_max_ 3312, 3273, 2967, 1645, 1637, 1225, 746 cm^−1^; HRESIMS at *m/z* 251.1159 [M + Na]^+^ (calcd for 251.1155). ^1^H and ^13^C NMR see Table 2. 

Eurotiumin E (**14**): yellow solid; [α${]}_{D}^{25}$ = +5.1 (c 0.1, CH_3_CN); UV (CH_3_CN) *λ*_max_ (log *ε*) 208 (4.40), 226 (4.37), 280 (4.20) nm; HRESIMS at *m/z* 335.1764 [M + H]^+^ (calcd for 335.1754). ^1^H and ^13^C NMR see Table 2.

### 3.6. Computational Methods

Molecular Merck force field (MMFF) calculations were done using Spartan’14 program (Wavefunction Inc., Irvine, CA, USA). Density functional theory (DFT) and time-dependent density functional theory (TDDFT) calculations were performed with Gaussian09 program package [27]. For conformational analysis, the conformers generated by a MMFF conformational search in an energy window of 10 kcal/mol were subjected to geometry optimization using the DFT method at the B3LYP/def2-SVP level. Frequency calculations were run at the same level to estimate their relative thermal (Δ*E*) and free energies (Δ*G*) at 298.15 K. Energies of the low-energy conformers were re-calculated at the M06-2X/def2-TZVP level. Solvent (MeOH) effects were taken into account by using polarizable continuum model (IEFPCM). The TDDFT calculations were performed using the hybrid PBE1PBE, CAM-B3LYP, and M06-2X functionals, and the Ahlrichs’ basis sets TZVP [28]. The number of excited states was 36 for both compounds. The ECD spectra were generated by the program SpecDis [29] using a Gaussian band shape from dipole-length dipolar and rotational strengths. The equilibrium population of each conformer at 298.15 K was calculated from its Δ*G* using Boltzmann statistics. The calculated spectra of compounds were generated from the low-energy conformers according to the Boltzmann weighting of each conformer in MeOH solution.

### 3.7. X-ray Crystal Structure Analysis

Crystallographic data for compound eurotiumin A (**1**) was collected on a Rigaku XtaLAB AFC12 single-crystal diffractometer using Cu Kα radiation. The structure of **1** was solved by direct methods (SHELXS97), expanded using difference Fourier techniques, and refined by full-matrix least-squares calculation. The non-hydrogen atoms were refined anisotropically, and hydrogen atoms were fixed at calculated positions. Crystallographic data for the structure of eurotiumin A (**1**) had been deposited in the Cambridge Crystallographic Data Centre database (deposition number CCDC 1829910). Copies of the data could be obtained free of charge from the CCDC at www.ccdc.cam.ac.uk.

Crystal data for **1**: C_19_H_23_N_3_O_3_, M = 341.40, tetragonal, space group *P*41, *a* = 7.91520(10) Å, *b* = 7.91520(10) Å, *c* = 28.2961(5) Å, *V* = 1772.76(5) Å^3^, *a* = 90°, *b* = 90°, *g* = 90°, *Z* = 4, *T* = 299.8(2) K, *μ*(Cu Kα) = 0.712 mm^−1^, *D*_calc_ = 1.279 g/cm^3^, 9129 reflections measured (11.178° ≤ 2Θ ≤ 147.856°), 3483 unique (*R*_int_ = 0.0191, *R*_sigma_ = 0.0215), which were used in all calculations. The final *R*_1_ was 0.0422 (*I* > 2σ(*I*)) and *wR*_2_ was 0.1256 (all data). The goodness of fit on *F*^2^ was 1.066. Flack parameter = 0.04(6).

### 3.8. Antioxidative Assay

In the radical-scavenging assay, 200 μL of reaction mixture containing test sample and 200 μM of 1,1-diphenyl-2-picrylhydrazyl (DPPH), dissolved in EtOH, was plated in 96-well plates and incubated at 37 °C in the dark for 30 min [25]. After the reaction, the UV/VIS absorbance was measured at 517 nm, and the percent inhibition was calculated. IC_50_ values denoted the concentration of sample required to scavenge 50% of the DPPH free radicals.

### 3.9. Cytotoxic Assay 

New compounds **1**–**4** and **14** were evaluated for their cytotoxic activities against SF-268 and HepG2 cell lines with the SRB method [20]. Cells (180 μL) with a density of 3 × 10^4^ cells/mL of media were seeded onto 96-well plates and incubated for 24 h at 37 °C, 5% CO_2_. Twenty μL of various concentrations of compounds were added into each plate well. The plates were further incubated for 72 h. After incubation, cell monolayers were fixed with 50% (*w*/*v*) trichloroacetic acid (50 μL) and stained for 30 min with 0.4% (*w*/*v*) SRB dissolved in 1% acetic acid. Unbound dye was removed by washing repeatedly with 1% acetic acid. The protein-bound dye was dissolved in 10 mM Tris base solution (200 µL) for OD determination at 570 nm using a microplate reader. Taxol was used as a positive control possessing potent cytotoxic activity. All data were obtained in triplicate and presented as means ± SD. IC_50_ values were calculated with the Sigma Plot 10.0 software using a non-linear curve-fitting method.

## 4. Conclusions

Chemical investigation of a marine-derived fungus *Eurotium* sp. SCSIO F452 led to isolation and identification of three new prenylated indole 2,5-diketopiperazine alkaloids (**1**–**3**) with nine known ones (**5**–**13**), one new prenylated indole alkaloid (**4**), and one new bis-benzyl pyrimidine derivative (**14**). **1** and **2** both shared a hexahydropyrrolo[2,3-*b*]indole skeleton, and occurred as a pair of diastereomers. The absolute configuration of **1** was determined by quantum chemical calculations of ECD and single crystal X-ray diffraction experiment. **14** was presented as a 2,5-bis-benzyl pyrimidine derivative rarely from marine-derived fungus. Multiple prenylated DKPs implied the existence of prenyltransferase in this fungus [30,31]. In addition, compounds **3**, **5**, **6**, **7**, **9**, **10**, and **12** showed significant radical scavenging activities against DPPH with IC_50_ values of 13, 19, 4, 3, 24, 13, and 18 µM, respectively. 

## Figures and Tables

**Table 1:** ^1^H and ^13^C NMR Data for **1**–**3**^a^ (700, 175 MHz, TMS, *δ* in ppm, *J* in Hz).

Position	1		2		3	
	*δ*_C_	*δ*_H_ (*J*, Hz)	*δ*_C_	*δ*_H_ (*J*, Hz)	*δ*_C_	*δ*_H_ (*J*, Hz)
1		6.53 s		6.58 br s		11.12 s
2	92.4		95.4		144.3	
3	89.4		88.3		103.3	
3a	132.2		132.7		125.9	
4	124.5	7.28 d (7.6)	125.5	7.17 d (7.4)	118.8	7.18 d (7.9)
5	120.1	6.76 overlap	119.9	6.66 overlap	119.6	7.02 td (7.5, 1.0)
6	130.8	7.12 td (7.6, 1.2)	130.3	7.02 td (7.5, 1.3)	120.9	7.10 td (7.6, 1.1)
7	111.8	6.75 overlap	111.3	6.64 overlap	111.7	7.43 d (8.1)
7a	150.6		149.9		135.1	
8	36.4	a 2.76 dd (12.7, 11.2)	36.8	a 3.35 dd (13.3, 2.3)	110.8	6.94 s
		b 2.56 dd (12.7, 7.6)		b 2.64 dd (13.4, 10.9)		
9	59.2	3.73 dd (11.2, 7.6)	59.4	4.41 dd (10.9, 2.5)	124.5	
10	170.6		169.3		157.6	
11		7.16 br s		6.85 br s		10.21 s
12	53.0	3.91 q (6.9)	51.0	4.11 q (6.7)	128.4	
13	174.1		170.3		156.4	
14						8.68 s
15	45.7		45.8		40.0	
16	146.5	6.36 dd (17.6, 10.9)	146.3	6.52 dd (17.6, 10.8)	145.1	6.08 dd (17.4, 10.5)
17	111.8	4.98 dd (17.7, 1.2)	112.4	5.14 dd (17.7, 1.6)	111.7	5.06 dd (10.4, 1.2)
		4.90 dd (10.9, 1.5)		5.02 dd (10.7, 1.6)		5.03 dd (17.4, 1.2)
18	24.7	1.27 s	24.7	1.36 s	27.5	1.48 s
19	25.7	1.29 s	25.5	1.39 s	27.5	1.48 s
20	15.8	1.33 d (6.9)	14.9	1.15 d (6.7)	112.9	5.87 q (7.6)
21					11.1	1.81 d (7.6)
3-OH		4.49 s		4.32 s		

^a^**1**–**2** in acetone-*d*_6_, **3** in dimethyl sulfoxide-*d*_6_.

**Table 2:** ^1^H and ^13^C NMR Data for **4** and **14** ^a^ (700, 175 MHz, TMS, *δ* in ppm, *J* in Hz).

Position	4		Position	14	
	*δ*_C_	*δ*_H_ (*J*, Hz)		*δ*_C_	*δ*_H_ (*J*, Hz)
1			1		
2	146.1		2	152.9	
3	109.8		3		
3a	128.5		4	154.4	
4	120.2	7.70 d (7.7)	5	152.2	
5	120.8	7.04 td (7.1, 1.2)	6	141.5	8.26 s
6	122.1	7.07 td (7.0, 1.3)	7	22.1	2.44 s
7	112.0	7.37 d (8.1)	1′	132.2	
7a	135.1		2′/6′	130.8	7.23 d (8.7)
8	169.1		3′/5′	114.8	6.85 d (8.6)
9	40.0		4′	159.3	
10	147.0	6.41 dd (17.5, 10.6)	7′	41.0	4.08 s
11	112.3	5.12 dd (17.5, 1.2)	4′-OMe	55.4	3.75 s
		5.06 dd (10.6, 1.2)	1″	131.3	
12	27.3	1.64 s	2″/6″	130.5	7.12 d (8.7)
13	27.3	1.64 s	3″/5″	114.7	6.82 d (8.7)
			4″	159.3	
			7″	40.7	4.01 s
			4″-OMe	55.4	3.73 s

^a^**4** and **14** in acetone-*d*_6_.

**Table 3:** Antioxidative activities of compounds **1**–**7**, **9**–**10**, and **12**–**14** (IC_50_, µM).

Nos.	1	2	3	4	5	6	7	9	10	12	13	14	V_C_
IC_50_	37	69	13	>100	19	4	3	24	13	18	35	>100	23

## Supplementary Materials

The following are available online at http://www.mdpi.com/1660-3397/16/4/136/s1. This section includes 1D, 2D NMR spectra and other spectroscopic data for new compounds **1**–**4** and **14**, and computational details of compounds **1** and **2**.
