# Supplementary material for: Eurotiumins A–E, Five New Alkaloids from the Marine-Derived Fungus Eurotium sp. SCSIO F452
Source: Mar Drugs. 2018 Apr 21;16(4):136. doi: 10.3390/md16040136 (PMC5923423; doi:10.3390/md16040136)
Supplement: Supplementary file 1 [file marinedrugs-16-00136-s001.pdf]

Supporting Information

Eurotiumins A–E, Five New Alkaloids from the Marine-Derived Fungus *Eurotium* sp. SCSIO F452

Wei-Mao Zhong [1,4], Jun-Feng Wang [1], Xue-Feng Shi [1], Xiao-Yi Wei [2], Yu-Chan Chen [3], Qi Zeng [1,4], Yao Xiang [1,4], Xia-Yu Chen [1,4], Xin-Peng Tian [1], Zhi-Hui Xiao [1], Wei-Min Zhang [3], Fa-Zuo Wang [1,*] and Si Zhang [1,*]

[1] CAS Key Laboratory of Tropical Marine Bio-resources and Ecology, RNAM Center for Marine Microbiology, Guangdong Key Laboratory of Marine Materia Medica, South China Sea Institute of Oceanology, Chinese Academy of Sciences, 164 West Xingang Road, Guangzhou 510301, China; wmzhong@scsio.ac.cn (W.-M.Z.); wangjunfeng@scsio.ac.cn (J.-F.W.); shixuefeng@scsio.ac.cn (X.-F.S.); 1520340009@qq.com (Q.Z.); xy920412@sina.cn (Y.X.); xychen1994@gmail.com (X.-Y.C.); xinpengtian@scsio.ac.cn (X.-P.T.); xzh@scsio.ac.cn (Z.-H.X.); wangfazuo@scsio.ac.cn (F.-Z.W.); zhsimd@scsio.ac.cn (S.Z.)

[2] Key Laboratory of Plant Resources Conservation and Sustainable Utilization, South China Botanical Garden, Chinese Academy of Sciences, Guangzhou 510650, China; wxy@scbg.ac.cn (X.-Y.W.)

[3] State Key Laboratory of Applied Microbiology Southern China, Guangdong Provincial Key Laboratory of Microbial Culture Collection and Application, Guangdong Open Laboratory of Applied Microbiology, Guangdong Institute of Microbiology, 100 Central Xianlie Road, Guangzhou 510070, China; 454423583@qq.com (Y.-C.C.); wmzhang58@qq.com (W.-M.Z.)

[4] University of Chinese Academy of Sciences, 19 Yuquan Road, Beijing 100049, China

* Correspondence: wangfazuo@scsio.ac.cn (F.-Z.W.); zhsimd@scsio.ac.cn (S.Z.); Tel.: +86-020-3406-3746

Table of Contents

Computational Details 4

Figure S3. The ^{1}H NMR spectrum of eurotiumin A (**1**) in CD_3COCD_3. 6

Figure S4. The ^{13}C NMR spectrum of eurotiumin A (**1**) in CD_3COCD_3. 7

Figure S5. The HSQC spectrum of eurotiumin A (**1**) in CD_3COCD_3. 8

Figure S6. The HMBC spectrum of eurotiumin A (**1**) in CD_3COCD_3. 9

Figure S7. The ^{1}H–^{1}H COSY spectrum of eurotiumin A (**1**) in CD_3COCD_3. 10

Figure S8. The NOESY spectrum of eurotiumin A (**1**) in CD_3COCD_3. 11

Figure S9. The HRESIMS spectrum of eurotiumin A (**1**). 12

Figure S10. The IR spectrum of eurotiumin A (**1**). 13

Figure S11. The UV spectrum of eurotiumin A (**1**). 14

Figure S12. The ^{1}H NMR spectrum of eurotiumin B (**2**) in CD_3COCD_3. 15

Figure S13. The ^{13}C NMR spectrum of eurotiumin B (**2**) in CD_3COCD_3. 16

Figure S14. The HSQC spectrum of eurotiumin B (**2**) in CD_3COCD_3. 17

Figure S15. The HMBC spectrum of eurotiumin B (**2**) in CD_3COCD_3. 18

Figure S16. The ^{1}H–^{1}H COSY spectrum of eurotiumin B (**2**) in CD_3COCD_3. 19

Figure S17. The NOESY spectrum of eurotiumin B (**2**) in CD_3COCD_3. 20

Figure S18. The HRESIMS spectrum of eurotiumin B (**2**). 21

Figure S19. The IR spectrum of eurotiumin B (**2**). 22

Figure S20. The UV spectrum of eurotiumin B (**2**). 23

Figure S21. The ^{1}H NMR spectrum of eurotiumin C (**3**) in DMSO-d_6. 24

Figure S22. The ^{13}C NMR spectrum of eurotiumin C (**3**) in DMSO-d_6. 25

Figure S23. The HSQC spectrum of eurotiumin C (**3**) in DMSO-d_6. 26

Figure S24. The HMBC spectrum of eurotiumin C (**3**) in DMSO-d_6. 27

Figure S25. The ^{1}H–^{1}H COSY spectrum of eurotiumin C (**3**) in DMSO-d_6. 28

Figure S26. The NOESY spectrum of eurotiumin C (**3**) in DMSO-d_6. 29

Figure S27. The HRESIMS spectrum of eurotiumin C (**3**). 30

Figure S28. The IR spectrum of eurotiumin C (**3**). 31

Figure S29. The UV spectrum of eurotiumin C (**3**). 32

Figure S30. The ^{1}H NMR spectrum of eurotiumin D (**4**) in CD_3COCD_3. 33

Figure S31. The ^{13}C NMR spectrum of eurotiumin D (**4**) in CD_3COCD_3. 34

Figure S32. The HSQC spectrum of eurotiumin D (**4**) in CD_3COCD_3. 35
Figure S33. The HMBC spectrum of eurotiumin D (**4**) in CD_3COCD_3. 36
Figure S34. The ^1H–^1H COSY spectrum of eurotiumin D (**4**) in CD_3COCD_3. 37
Figure S35. The NOESY spectrum of eurotiumin D (**4**) in CD_3COCD_3. 38
Figure S36. The HRESIMS spectrum of eurotiumin D (**4**). 39
Figure S37. The IR spectrum of eurotiumin D (**4**). 40
Figure S38. The UV spectrum of eurotiumin D (**4**). 41
Figure S39. The ^1H NMR spectrum of eurotiumin E (**14**) in CD_3COCD_3. 42
Figure S40. The ^{13}C NMR spectrum of eurotiumin E (**14**) in CD_3COCD_3. 43
Figure S41. The HSQC spectrum of eurotiumin E (**14**) in CD_3COCD_3. 44
Figure S42. The HMBC spectrum of eurotiumin E (**14**) in CD_3COCD_3. 45
Figure S43. The ^1H–^1H COSY spectrum of eurotiumin E (**14**) in CD_3COCD_3. 46
Figure S44. The HRESIMS spectrum of eurotiumin E (**14**). 47
Figure S45. The UV spectrum of eurotiumin E (**14**). 48
MS and NMR data of compounds 5–13 49

Computational Details

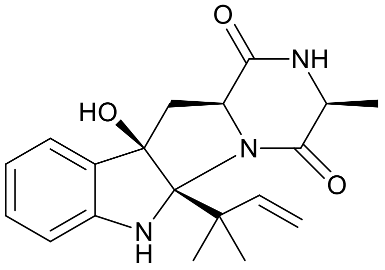

1

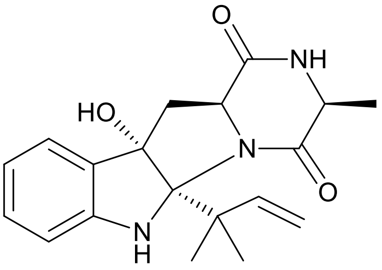

2

Figure S1. Structures applied for theoretical calculations of **1**, and **2**.

Table S1. Relative thermal energies (ΔE), relative free energies (ΔG), and equilibrium populations (P) of low-energy conformers of structures **1** and **2** in MeOH.

conformer	Δ*E* (kcal/mol) [a]	Δ*G* (kcal/mol) [a]	P (%)[b]
Compound **1**			
**1a**	0.0	0.0	69.7
**1b**	1.22	1.29	8.0
**1c**	1.33	1.31	7.6
**1d**	1.50	1.33	7.3
**1e**	1.62	1.56	5.0
**1f** [c]	1.82	1.99	2.4
Compound **2**			
**2a**	0.0	0.0	38.3
**2b**	0.35	0.31	22.8
**2c**	0.63	0.51	16.3
**2d**	0.58	0.70	11.7
**2e**	0.66	0.85	9.2
**2f** [c]	1.87	1.82	1.8

[a] At the M06-2X/def2-TZVP/ IEFPCM level of theory.
[b] From Δ*G* values at 298.15 K.
[c] Conformer not applied to ECD/TDDFT calculations.

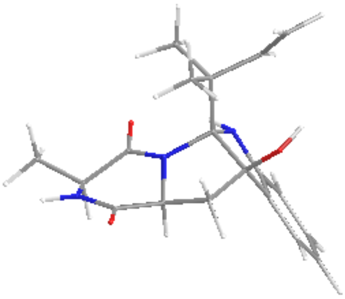

1a

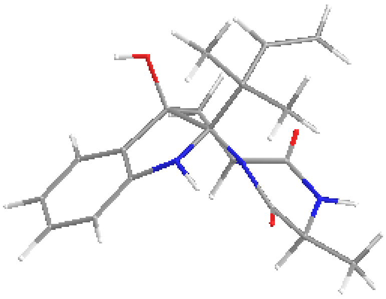

1b

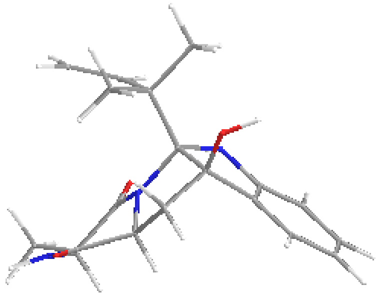

1c

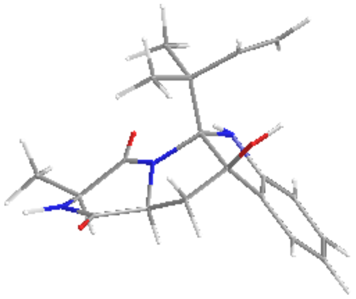

1d

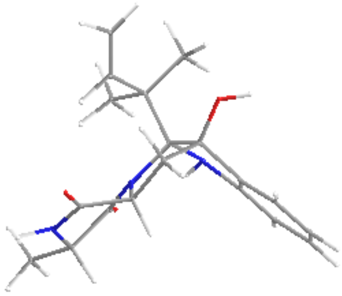

1e

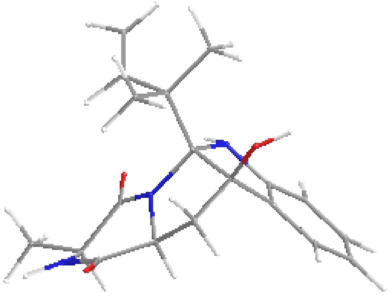

1f

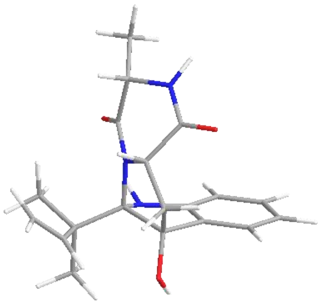

2a

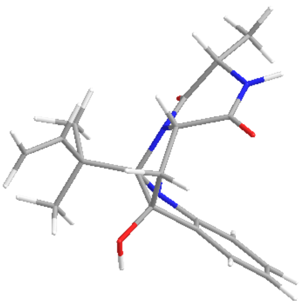

2b

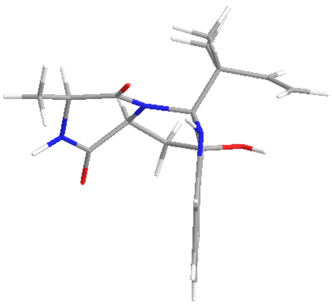

2c

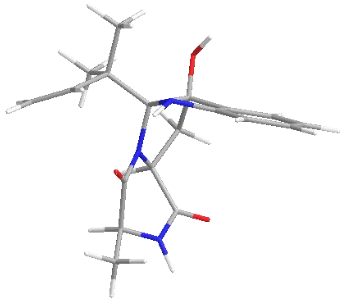

2d

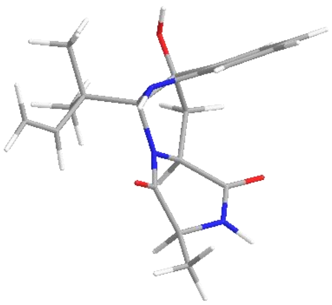

2e

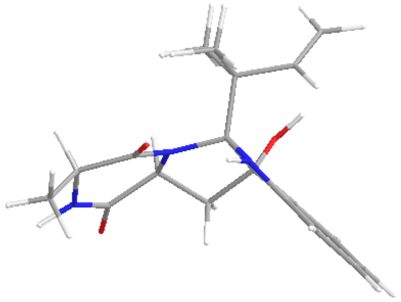

2f

Figure S2. Conformations of low-energy conformers of **1**, and **2**.

Figure S3. The ^{1}H NMR spectrum of eurotiumin A (**1**) in CD_3COCD_3.

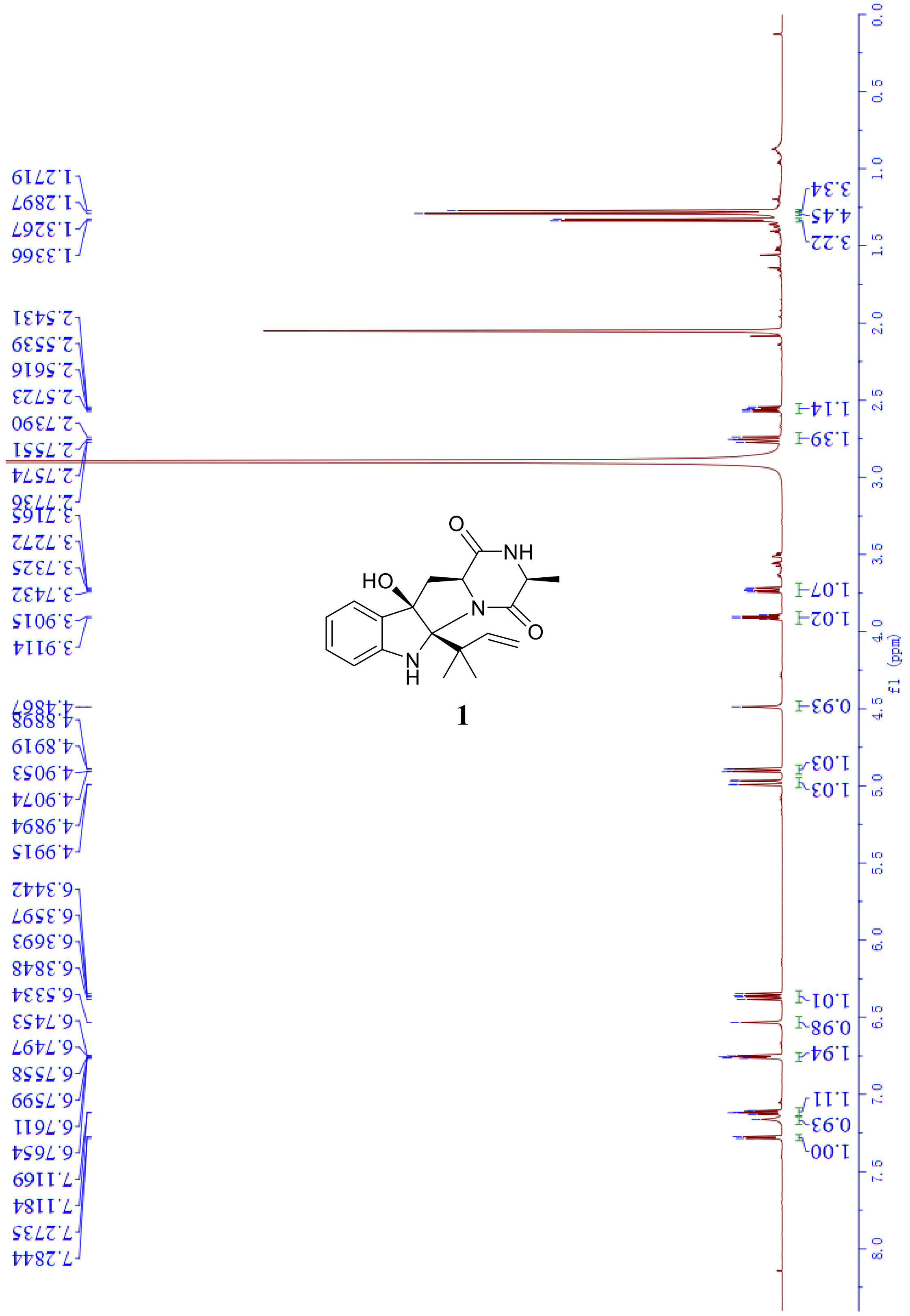

1

Figure S4. The ^{13}C NMR spectrum of eurotiumin A (**1**) in CD_3COCD_3.

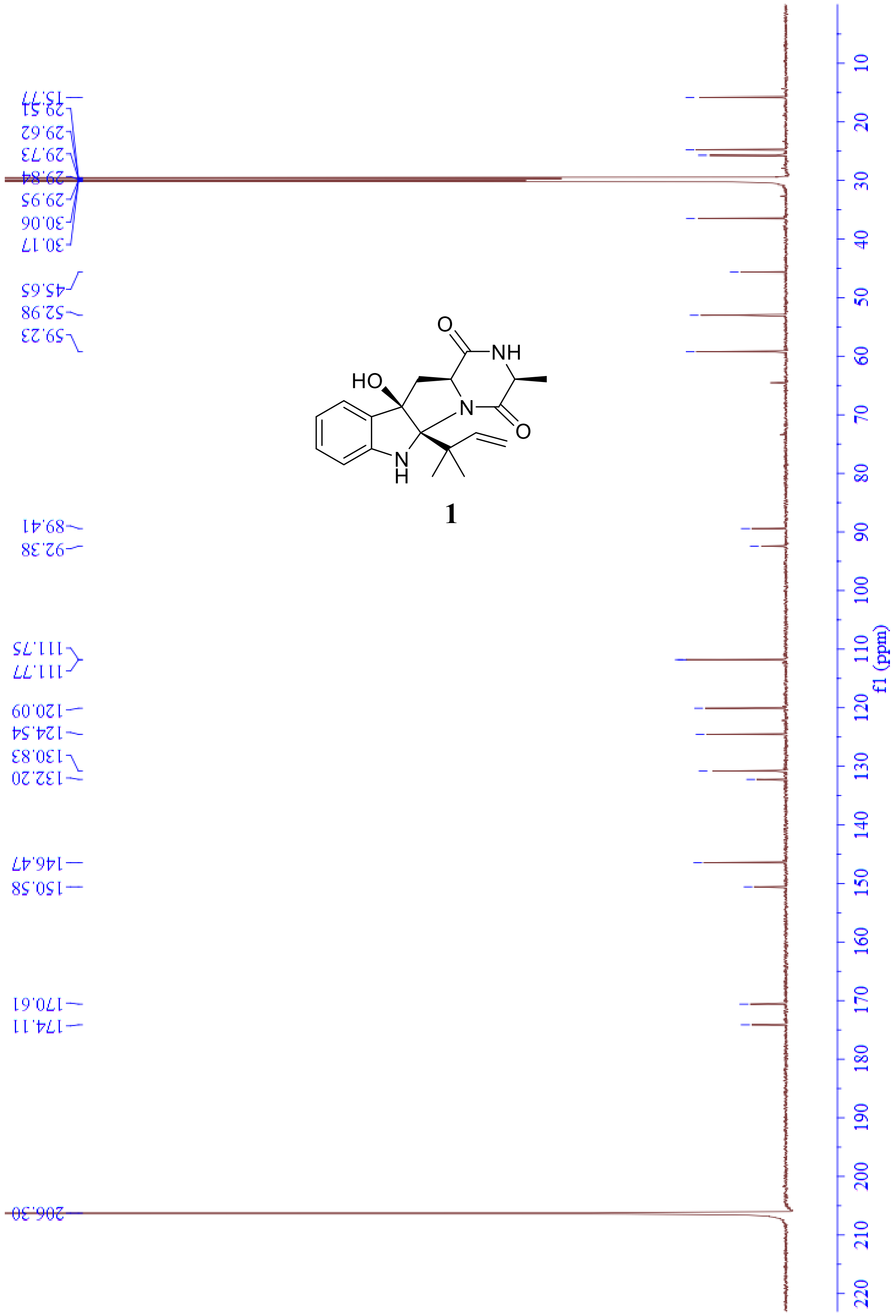

Figure S5. The HSQC spectrum of eurotiumin A (**1**) in CD_3COCD_3.

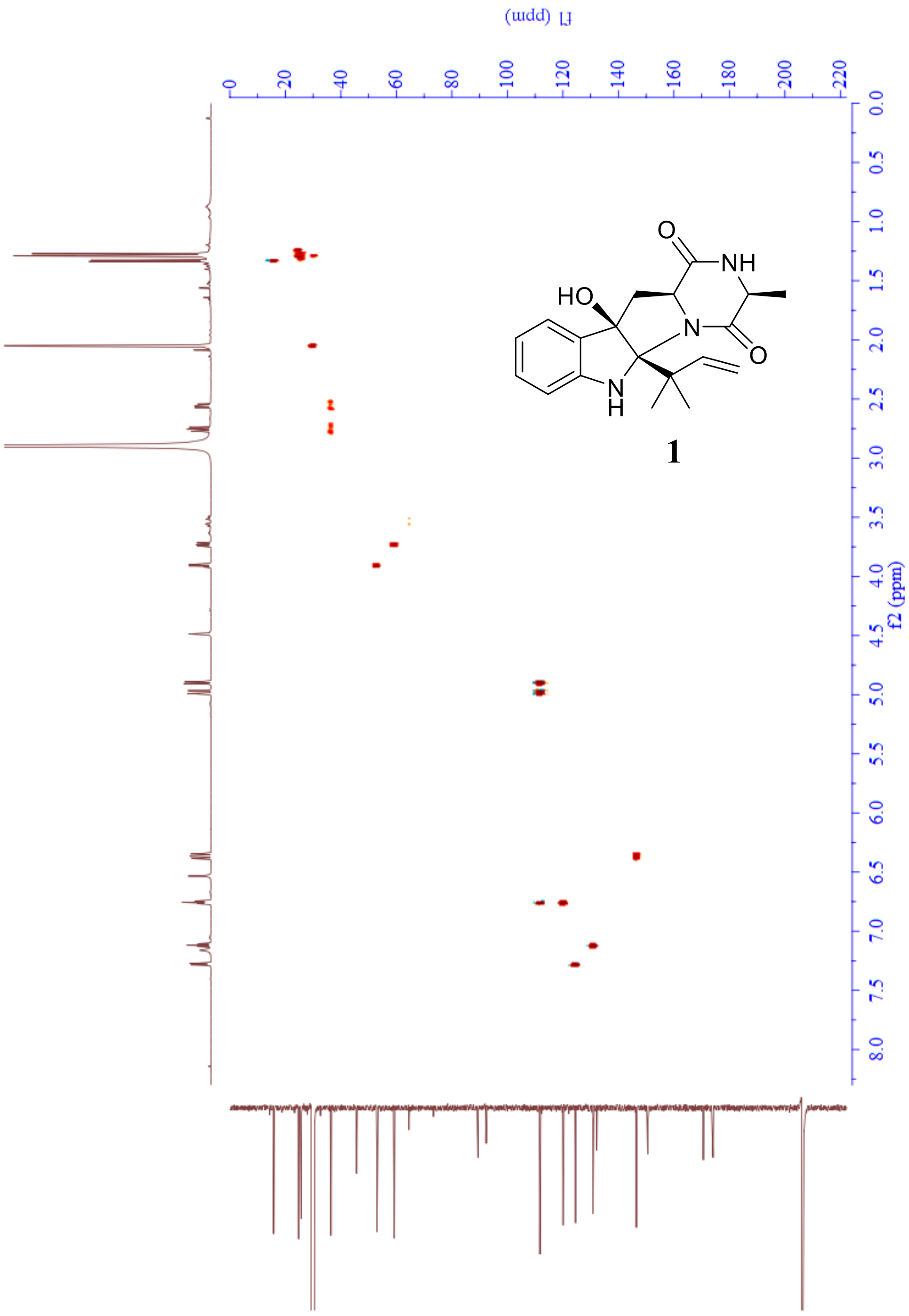

Figure S6. The HMBC spectrum of eurotiumin A (**1**) in CD_3COCD_3.

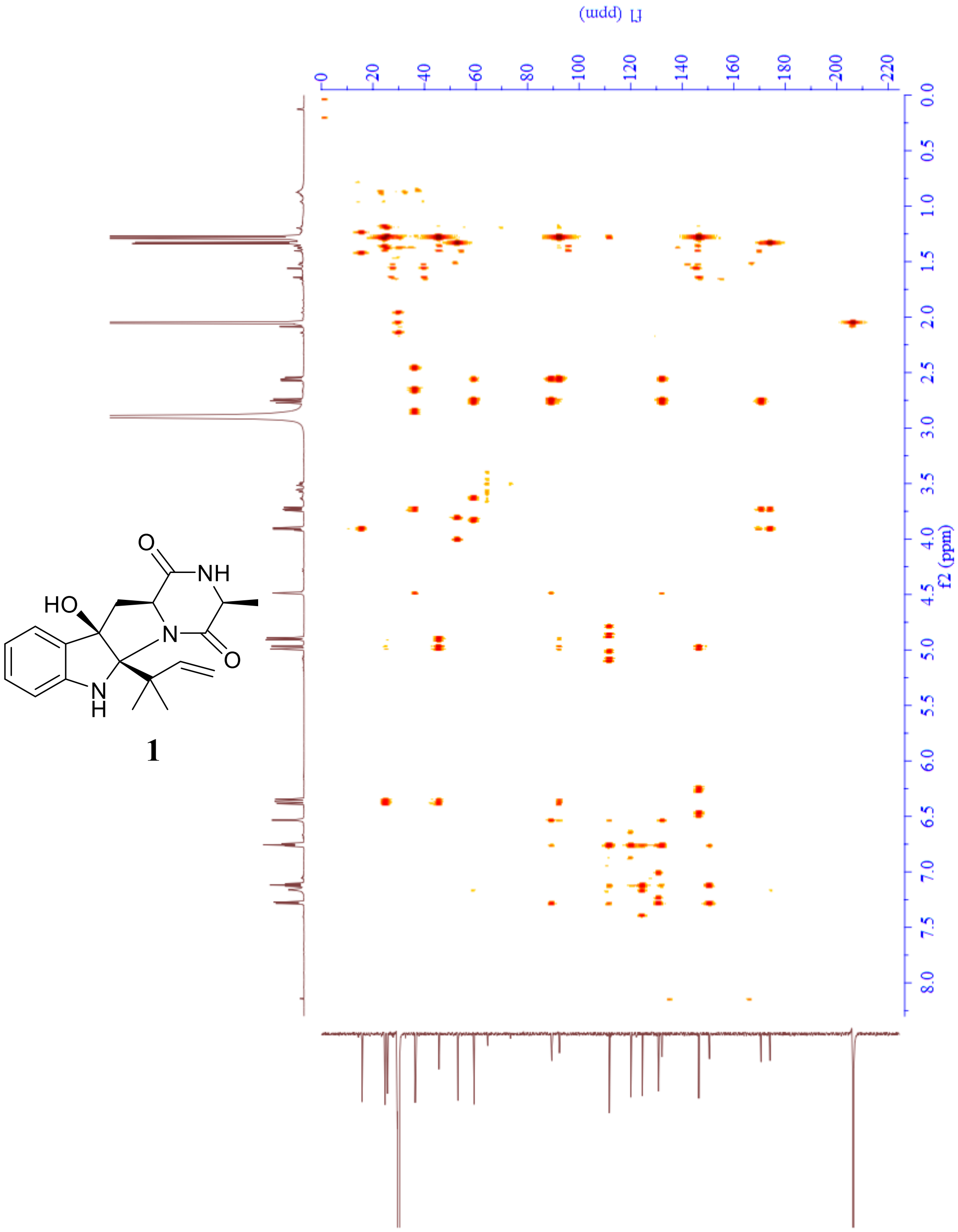

1

Figure S7. The ^1H–^1H COSY spectrum of eurotiumin A (**1**) in CD_3COCD_3.

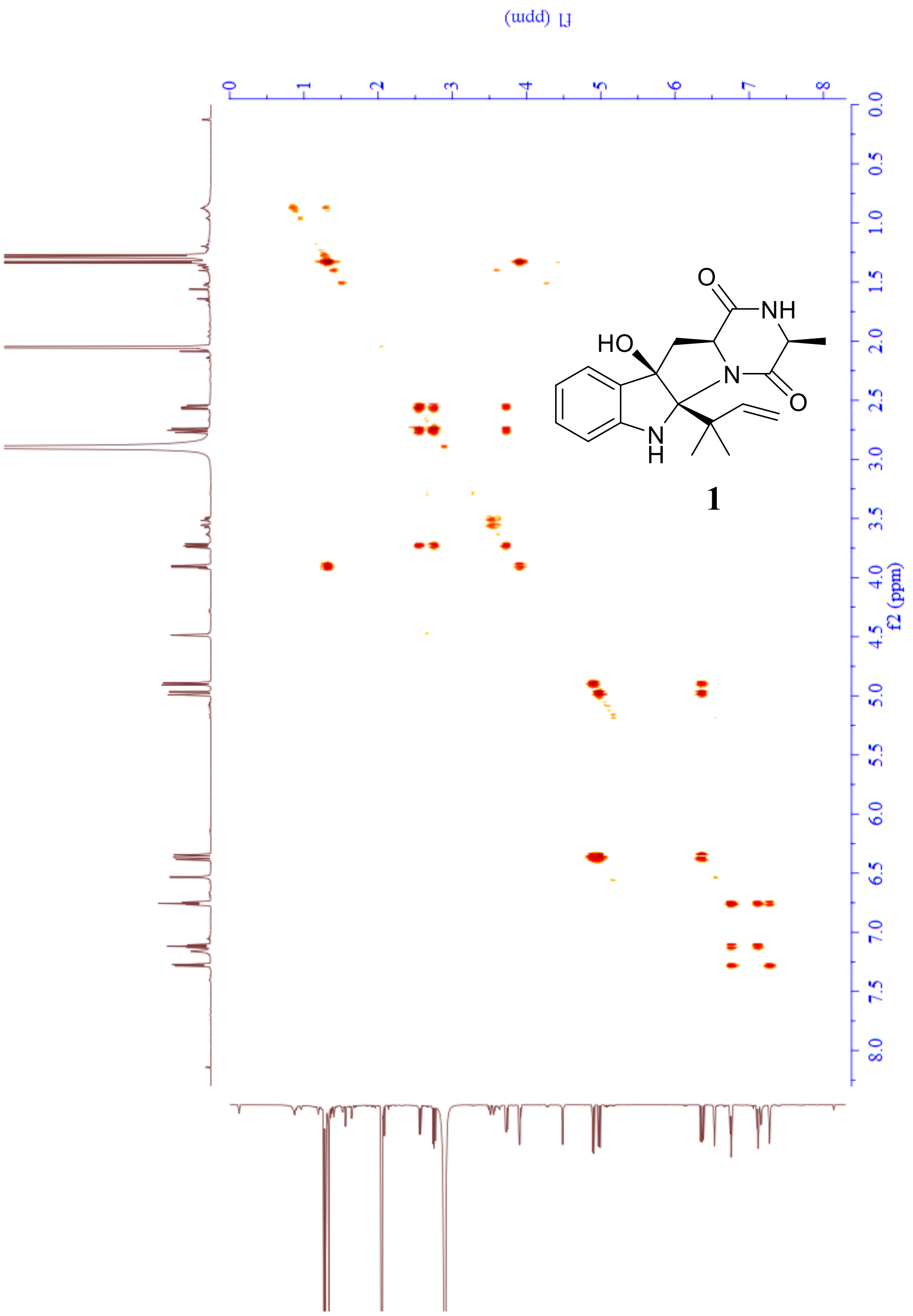

Figure S8. The NOESY spectrum of eurotiumin A (**1**) in CD_3COCD_3.

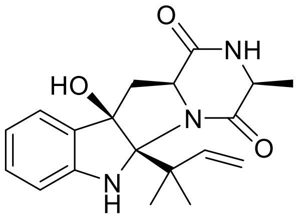

1

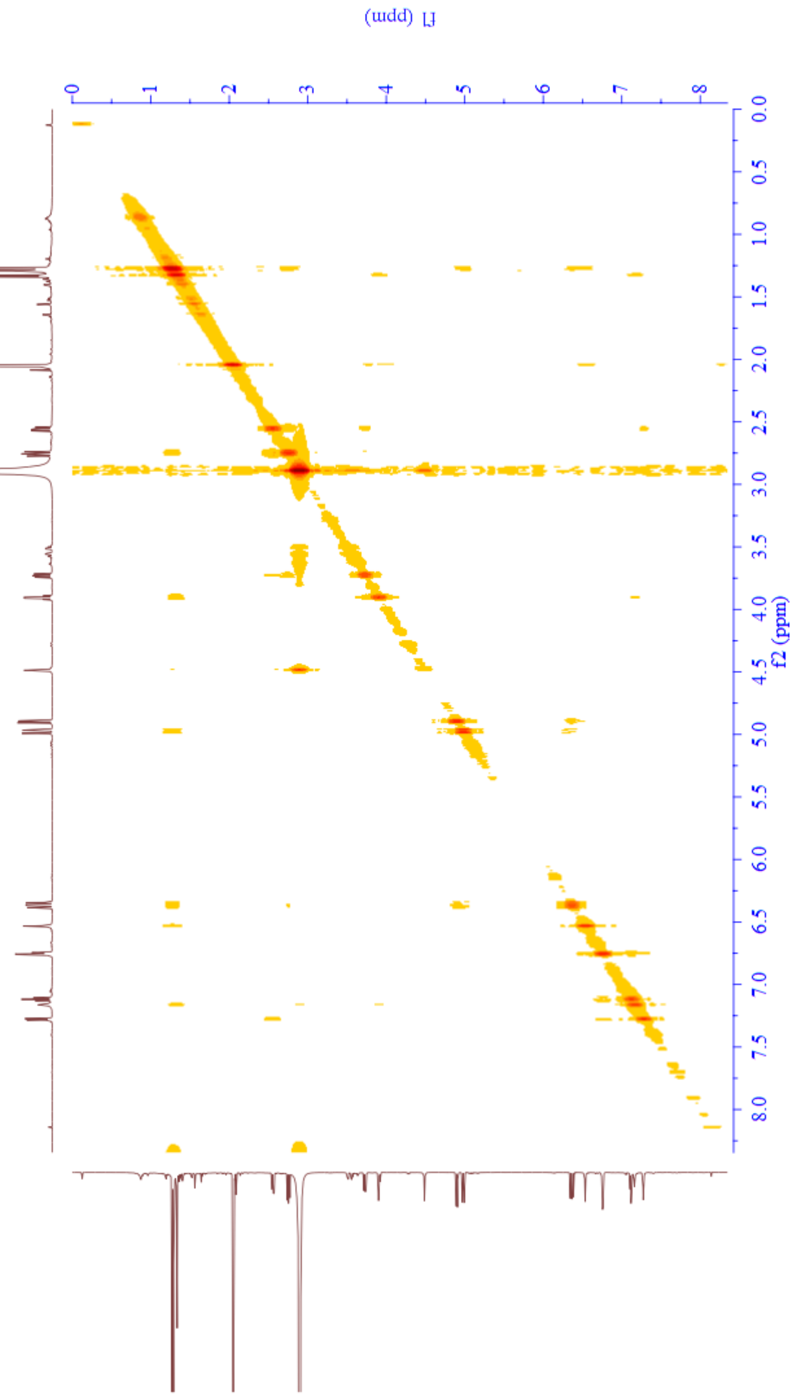

Figure S9. The HRESIMS spectrum of eurotiumin A (**1**).

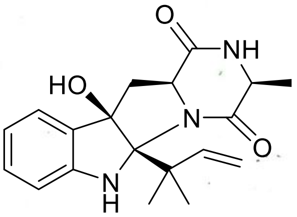

1

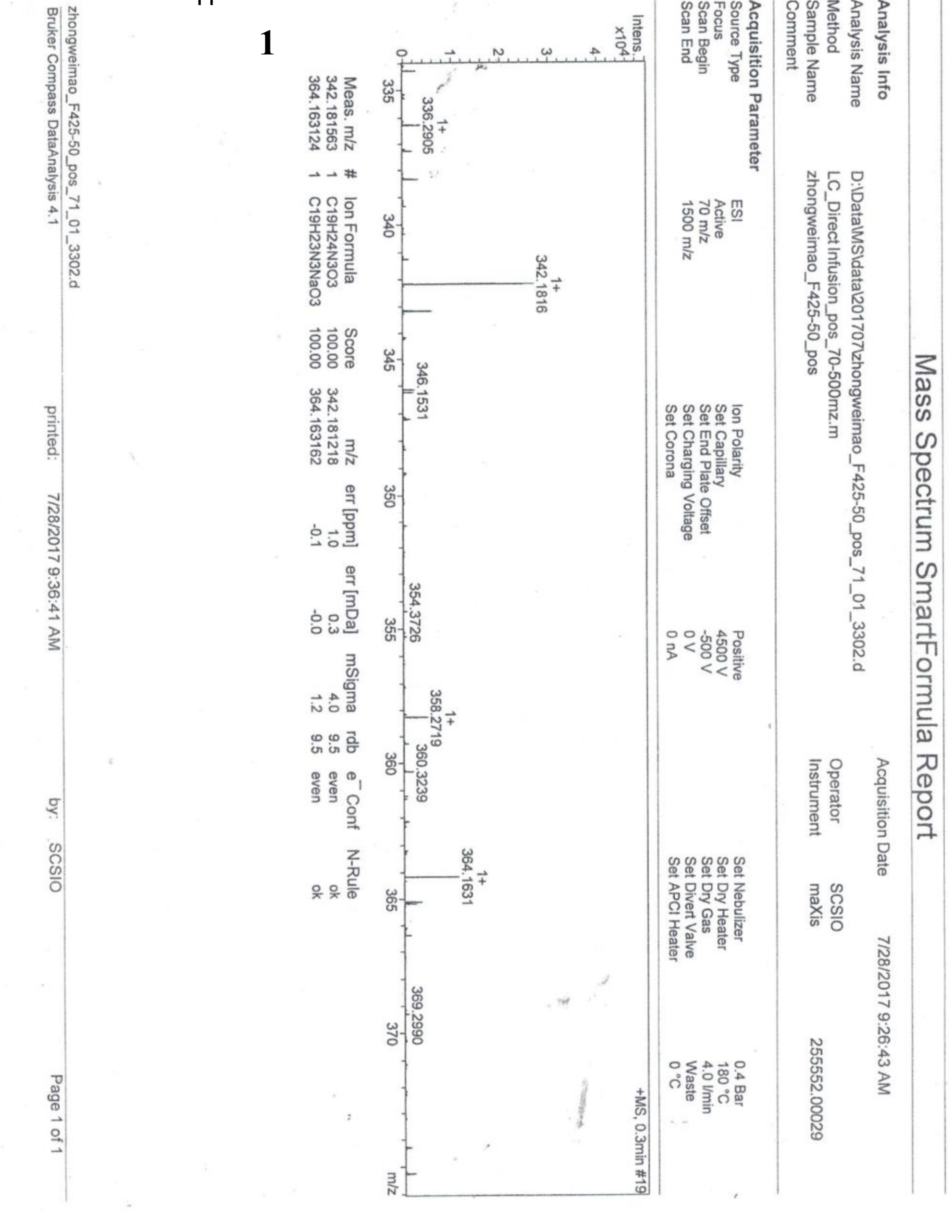

Figure S10. The IR spectrum of eurotiumin A (**1**).

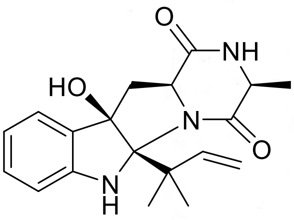

1

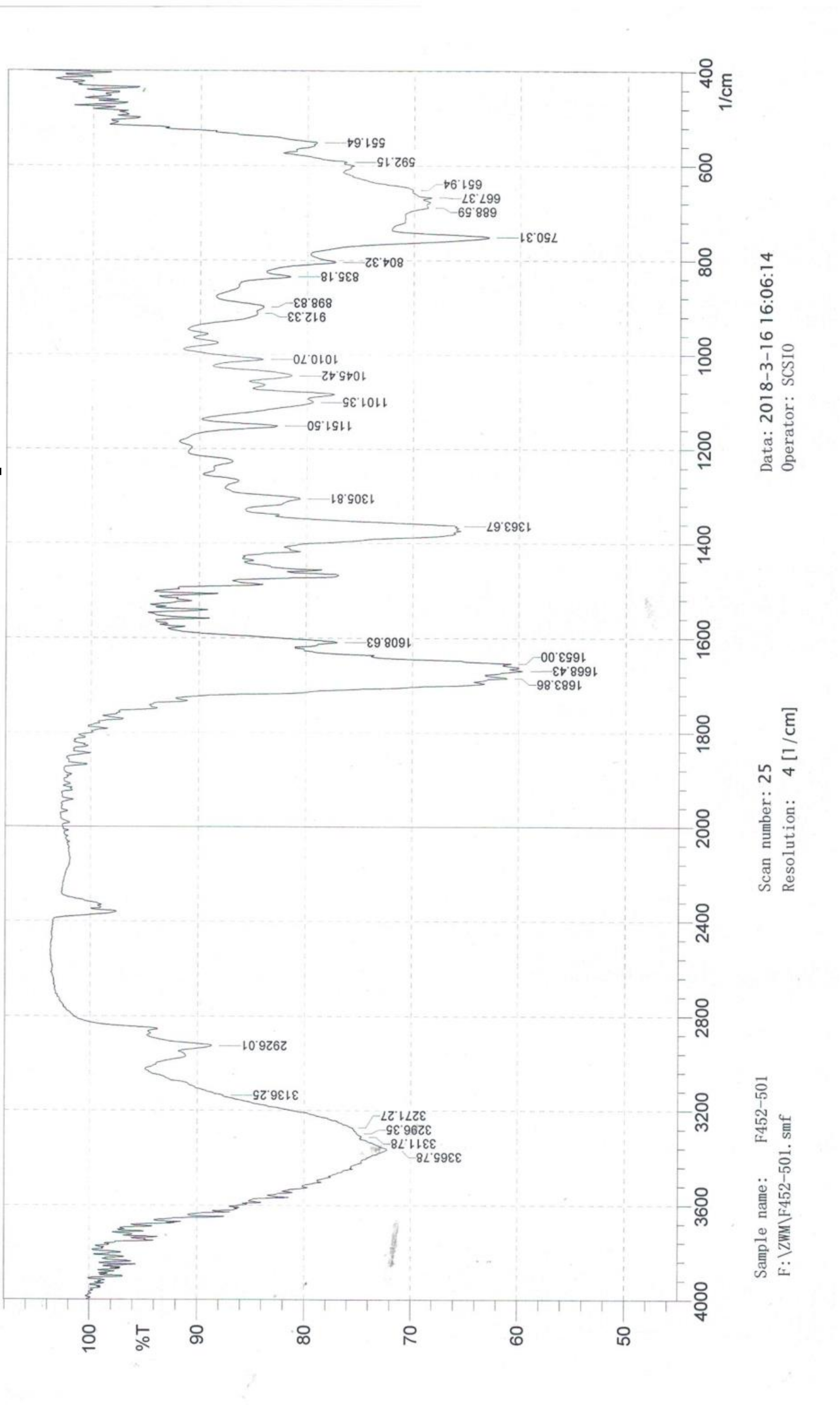

Figure S11. The UV spectrum of eurotiumin A (**1**).

光谱峰值检测报告

2017-12-26 18:31:52

数据集: F452-50-1 - RawData

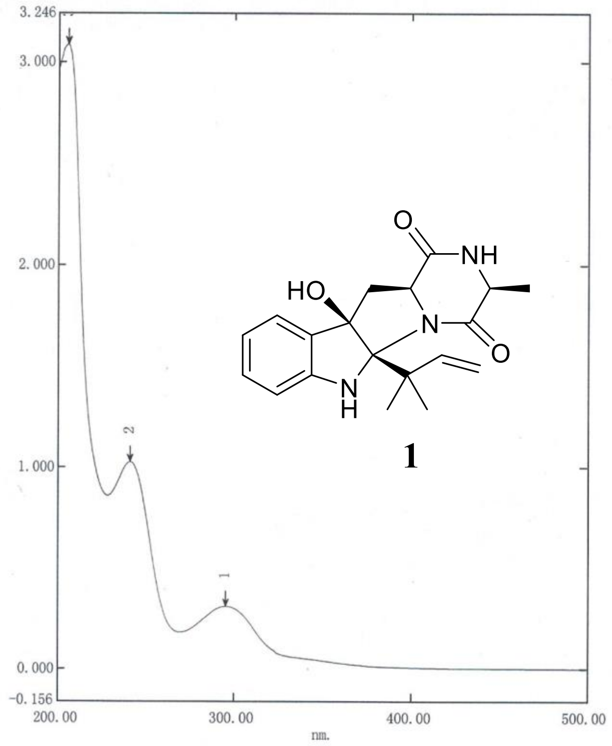

[测定属性]
波长范围 (nm.): 200.00 到 500.00
扫描速度: 中速
采样间隔: 0.2
自动采样间隔: 启用
扫描模式: 单个

[仪器属性]
仪器类型: UV-2600 系列
测定方式: 吸收值
狭缝宽: 2.0
积分时间: 0.1 秒.
光源转换波长: 323.0 nm
检测器单元: 直接
S/R 转换: 标准
阶梯校正: OFF

[附件属性]
附件: 无

[数据处理参数]
阈值: 0.0100000
点: 4
内插: 停用
平均: 停用

[样品准备属性]
重量:
体积:
稀释:
光程长:
附加信息:

No.	P/V	波长(nm)	吸收值	描述
1	↑	295.00	0.308	
2	↑	241.00	1.024	
3	↑	205.60	3.091	

页 1 / 1

Figure S12. The ^{1}H NMR spectrum of eurotiumin B (**2**) in CD_3COCD_3.

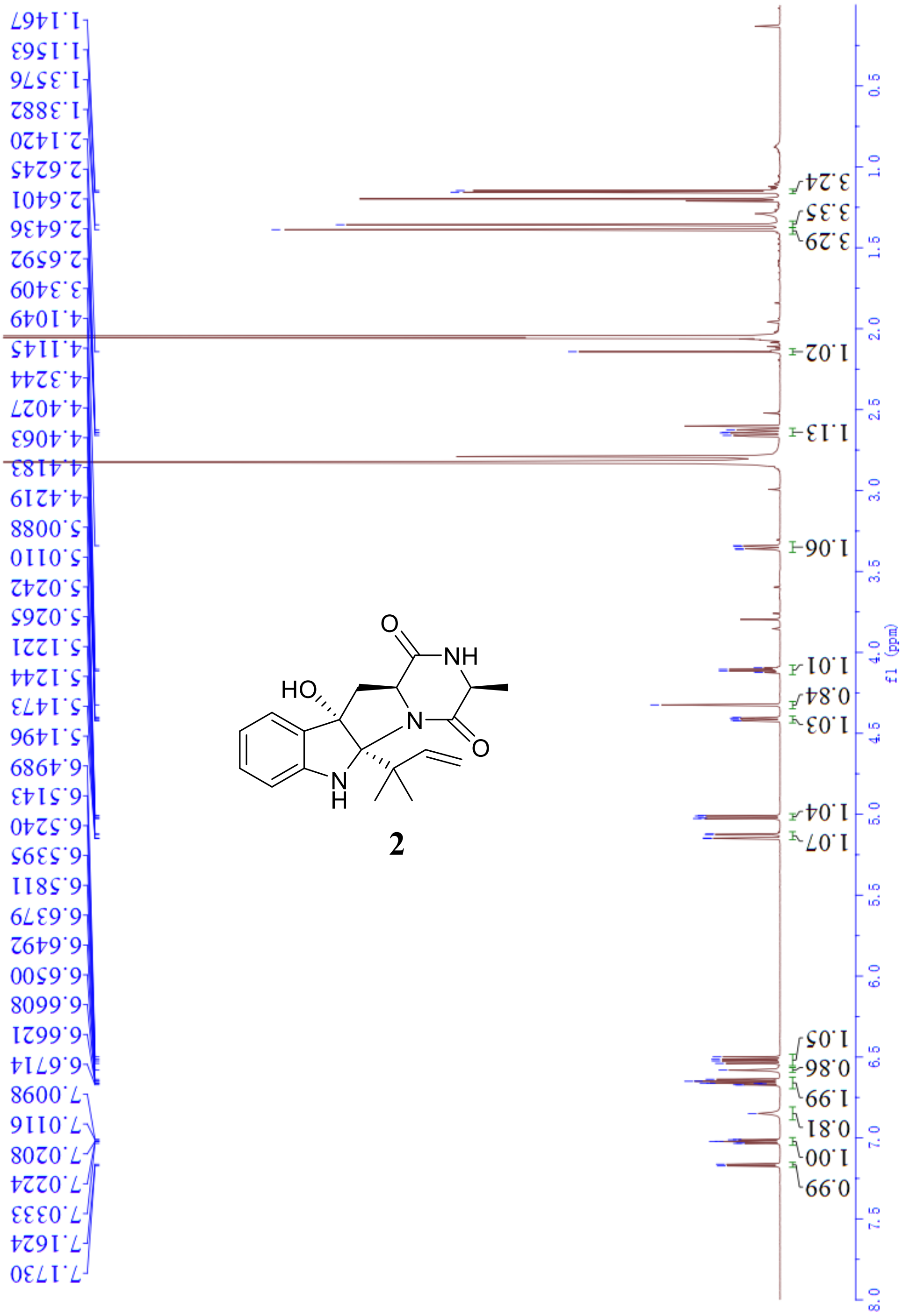

Figure S13. The ^{13}C NMR spectrum of eurotiumin B (**2**) in CD_3COCD_3.

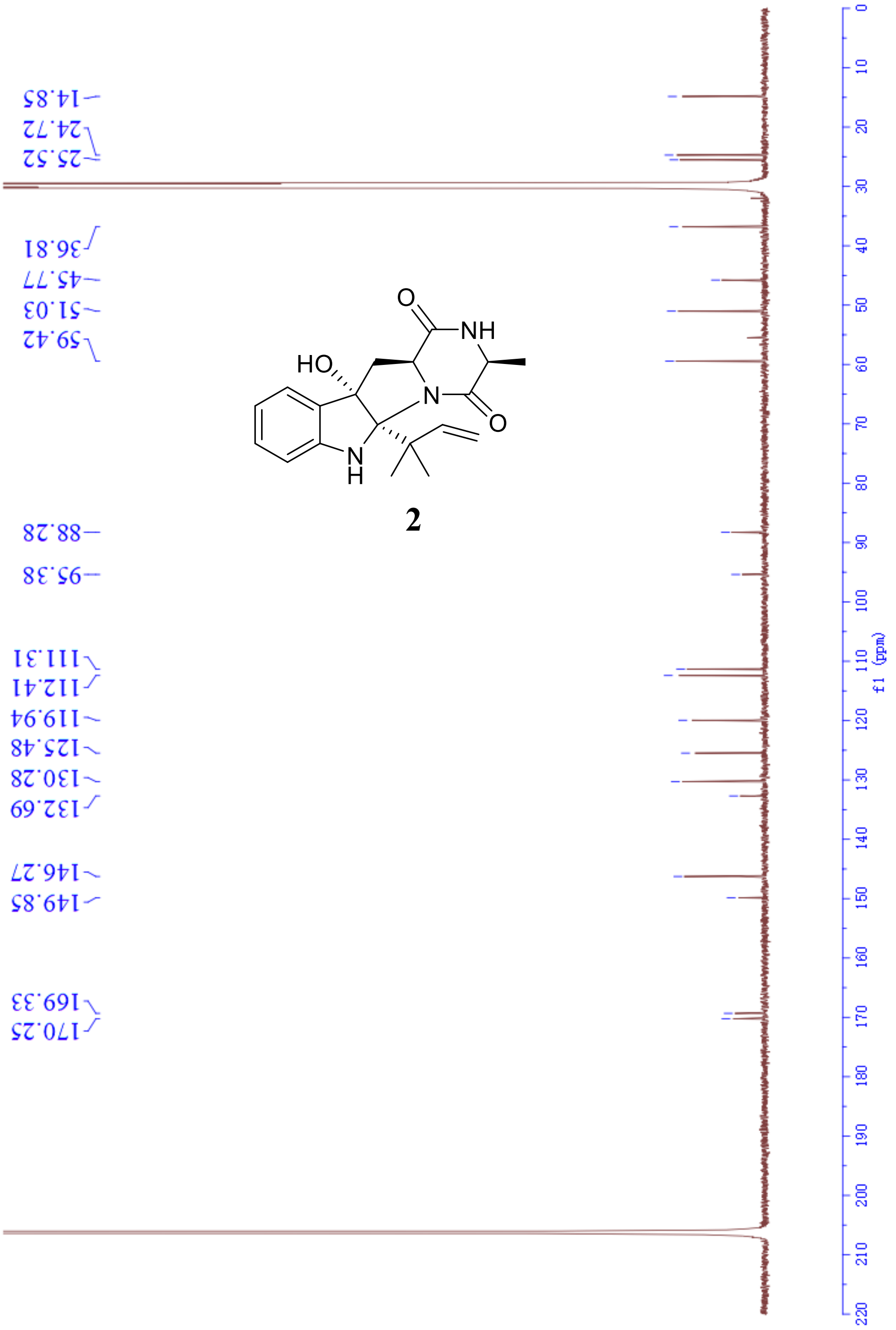

Figure S14. The HSQC spectrum of eurotiumin B (**2**) in CD_3COCD_3.

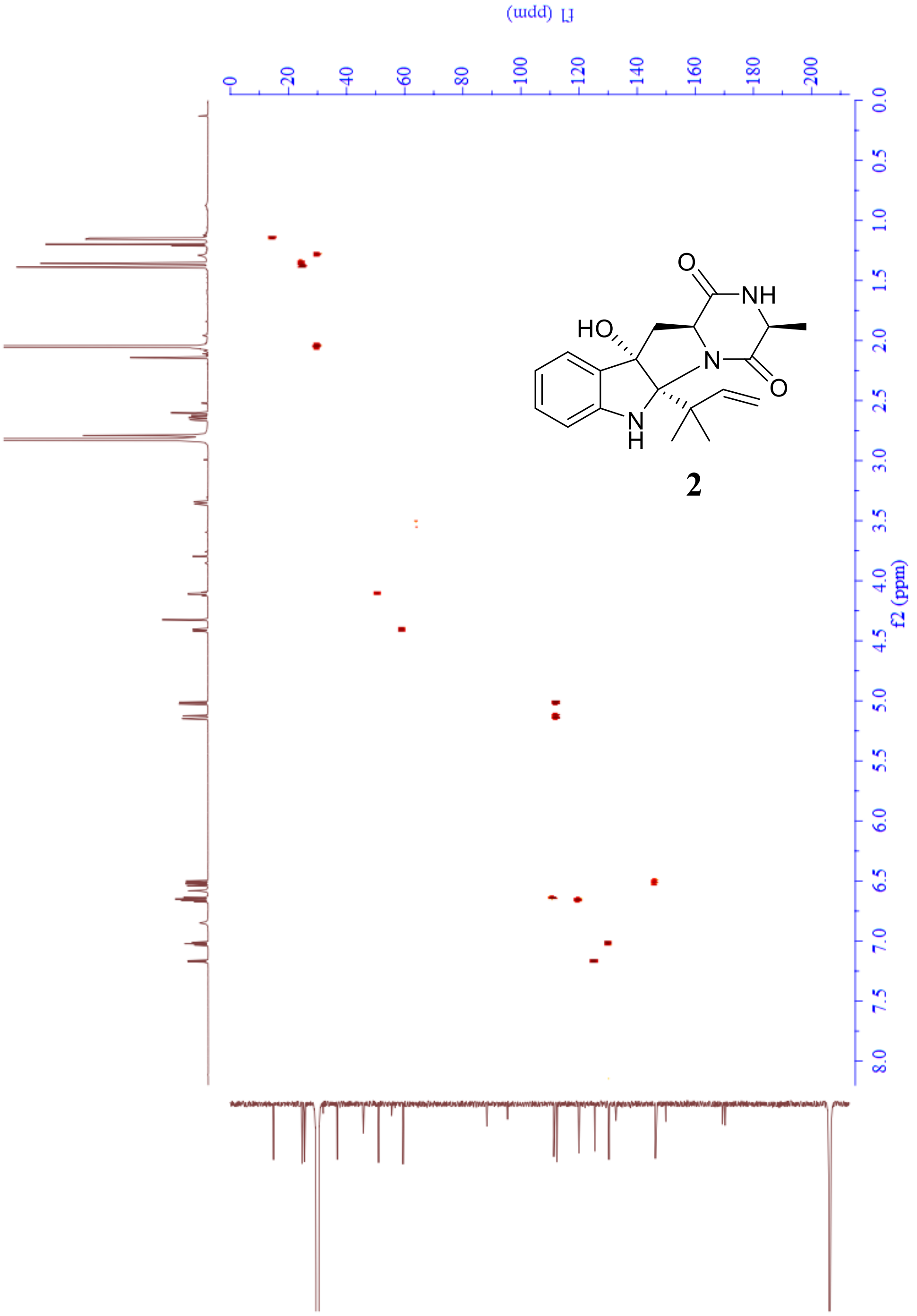

Figure S15. The HMBC spectrum of eurotiumin B (**2**) in CD_3COCD_3.

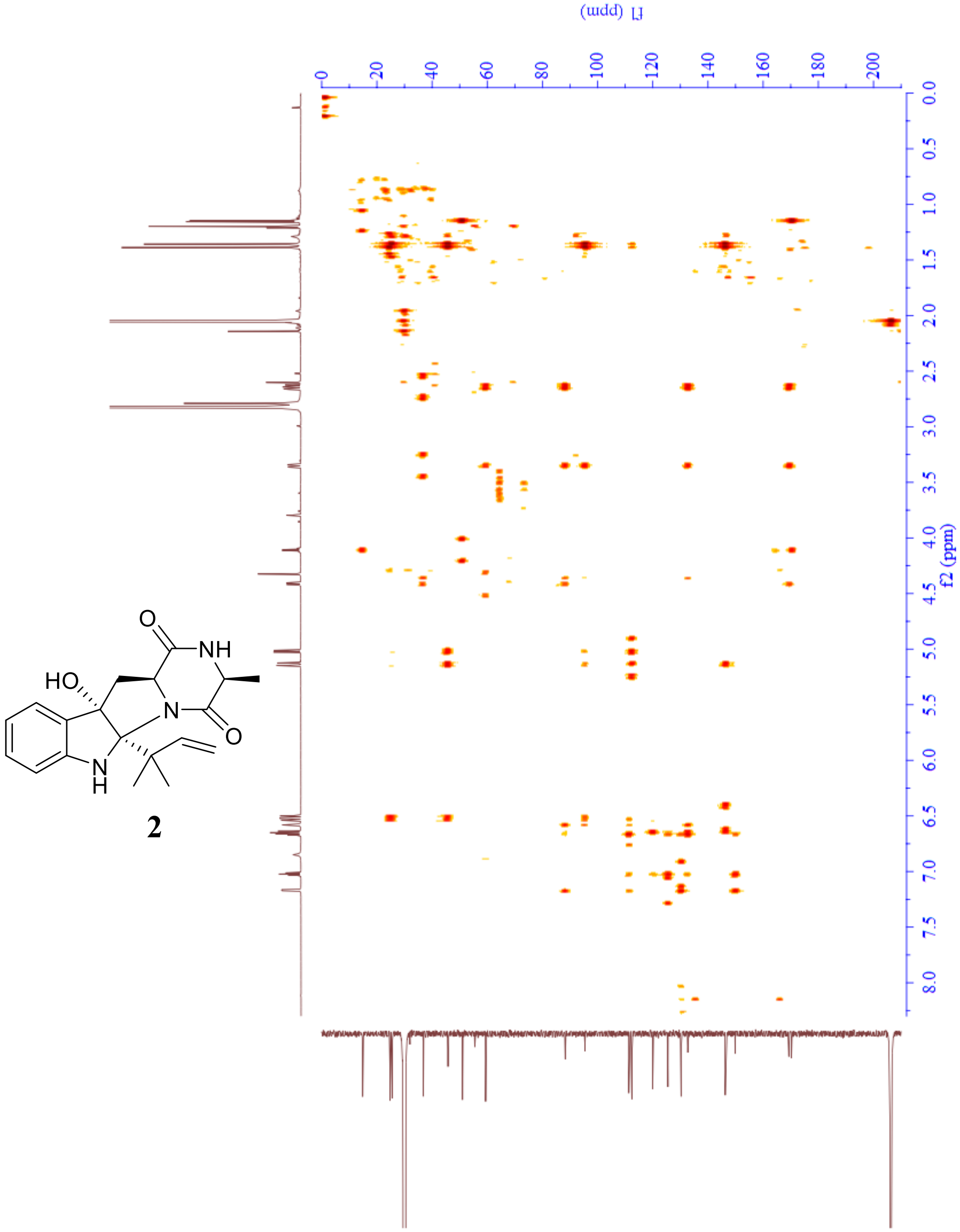

2

Figure S16. The ^{1}H–^{1}H COSY spectrum of eurotiumin B (**2**) in CD_3COCD_3.

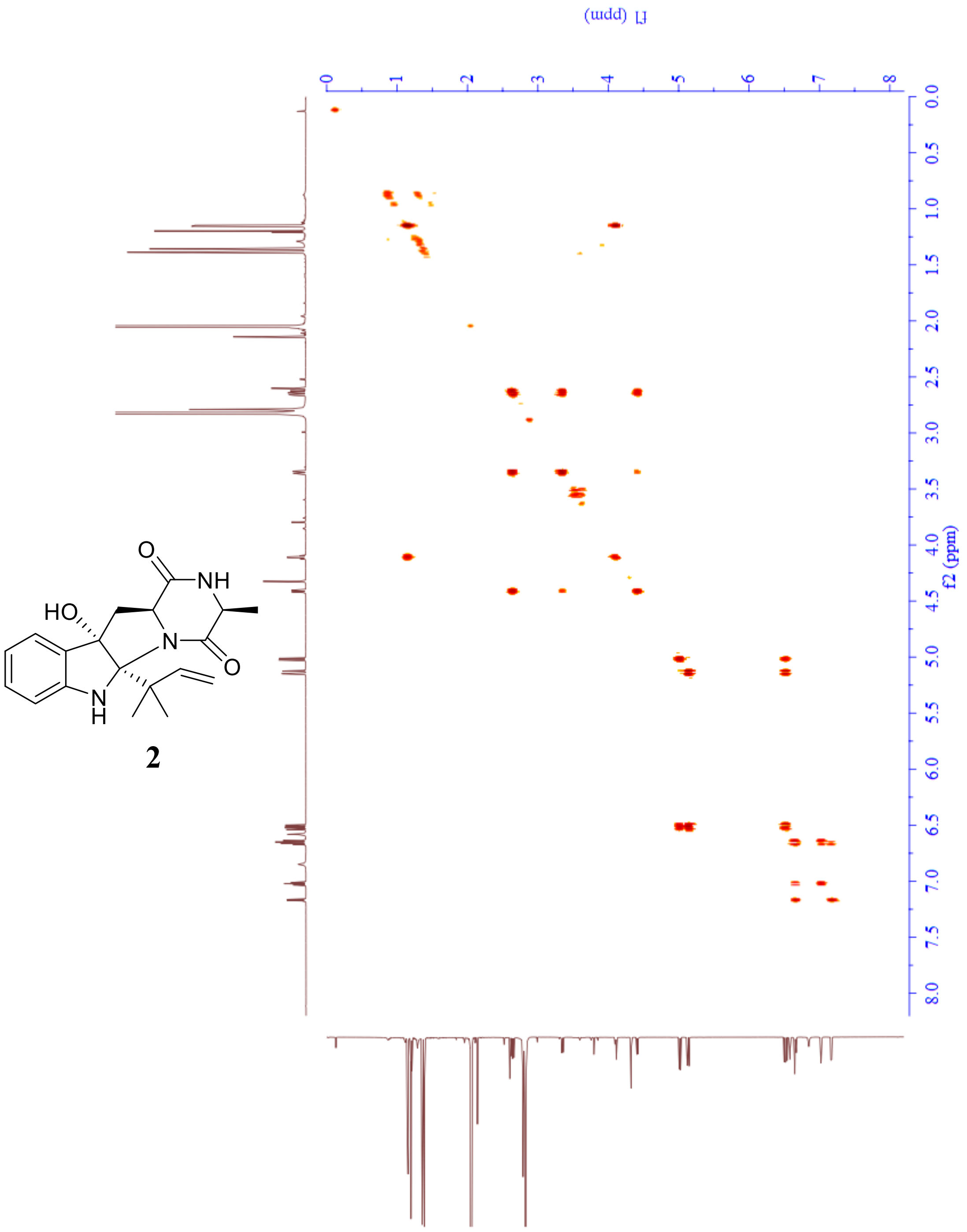

2

Figure S17. The NOESY spectrum of eurotiumin B (**2**) in CD_3COCD_3.

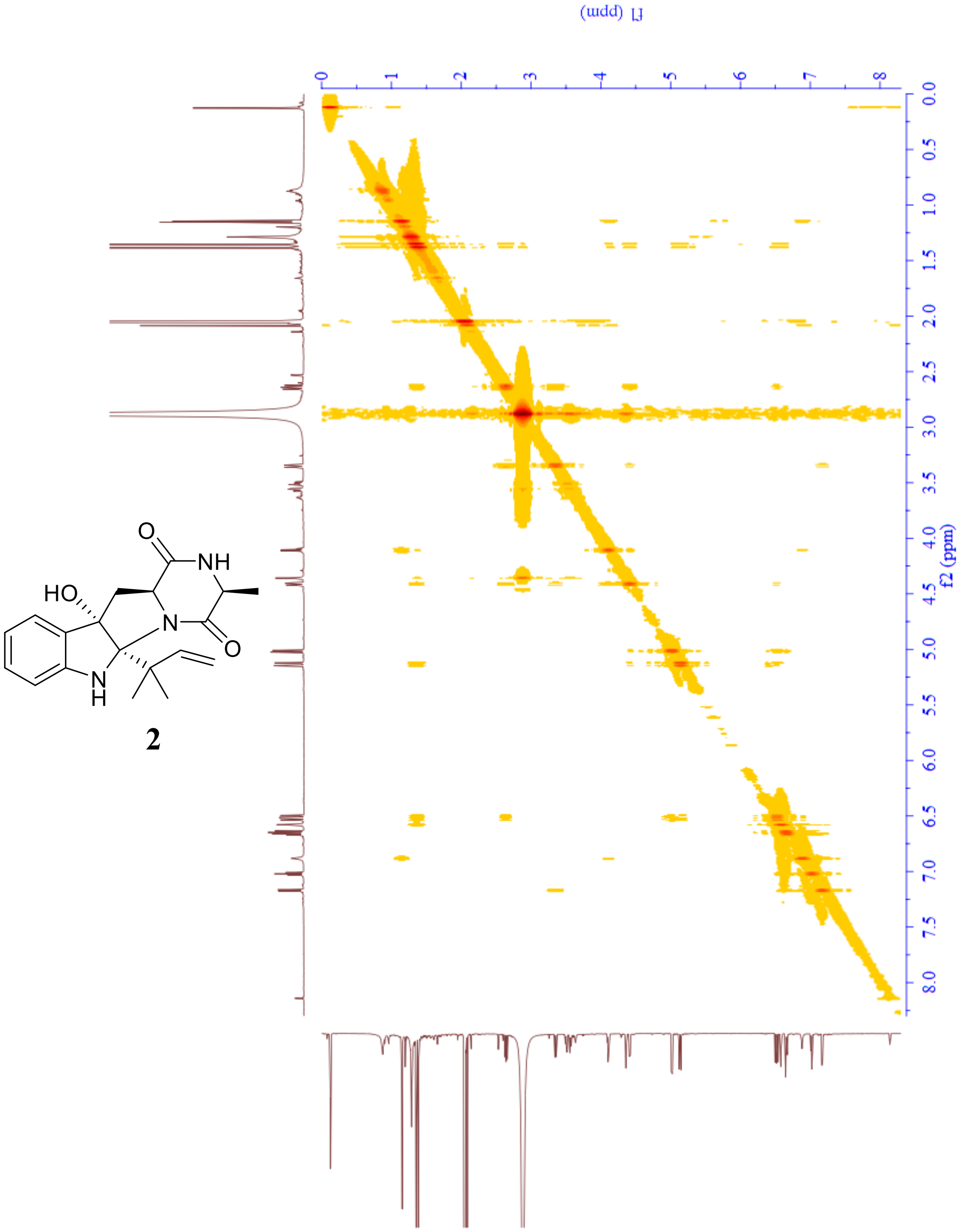

2

Figure S18. The HRESIMS spectrum of eurotiumin B (**2**).

光谱峰值检测报告

2018-01-04 17:46:30

数据集: F452-51-2 - RawData

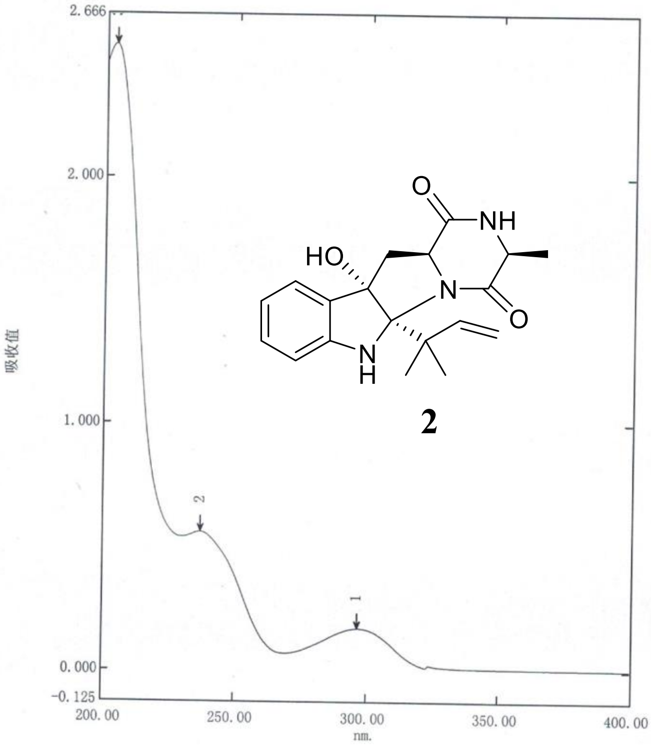

[测定属性]
波长范围 (nm.): 200.00 到 400.00
扫描速度: 中速
采样间隔: 0.2
自动采样间隔: 启用
扫描模式: 单个

[仪器属性]
仪器类型: UV-2600 系列
测定方式: 吸收值
狭缝宽: 2.0
积分时间: 0.1 秒.
光源转换波长: 323.0 nm
检测器单元: 直接
S/R 转换: 标准
阶梯校正: OFF

[附件属性]
附件: 无

[数据处理参数]
阈值: 0.0100000
点: 4
内插: 停用
平均: 停用

[样品准备属性]
重量:
体积:
稀释:
光程长:
附加信息:

No.	P/V	波长(nm)	吸收值	描述
1	⬆	296.40	0.168	
2	⬆	237.00	0.561	
3	⬆	203.60	2.539	

页 1/1

Figure S19. The IR spectrum of eurotiumin B (**2**).

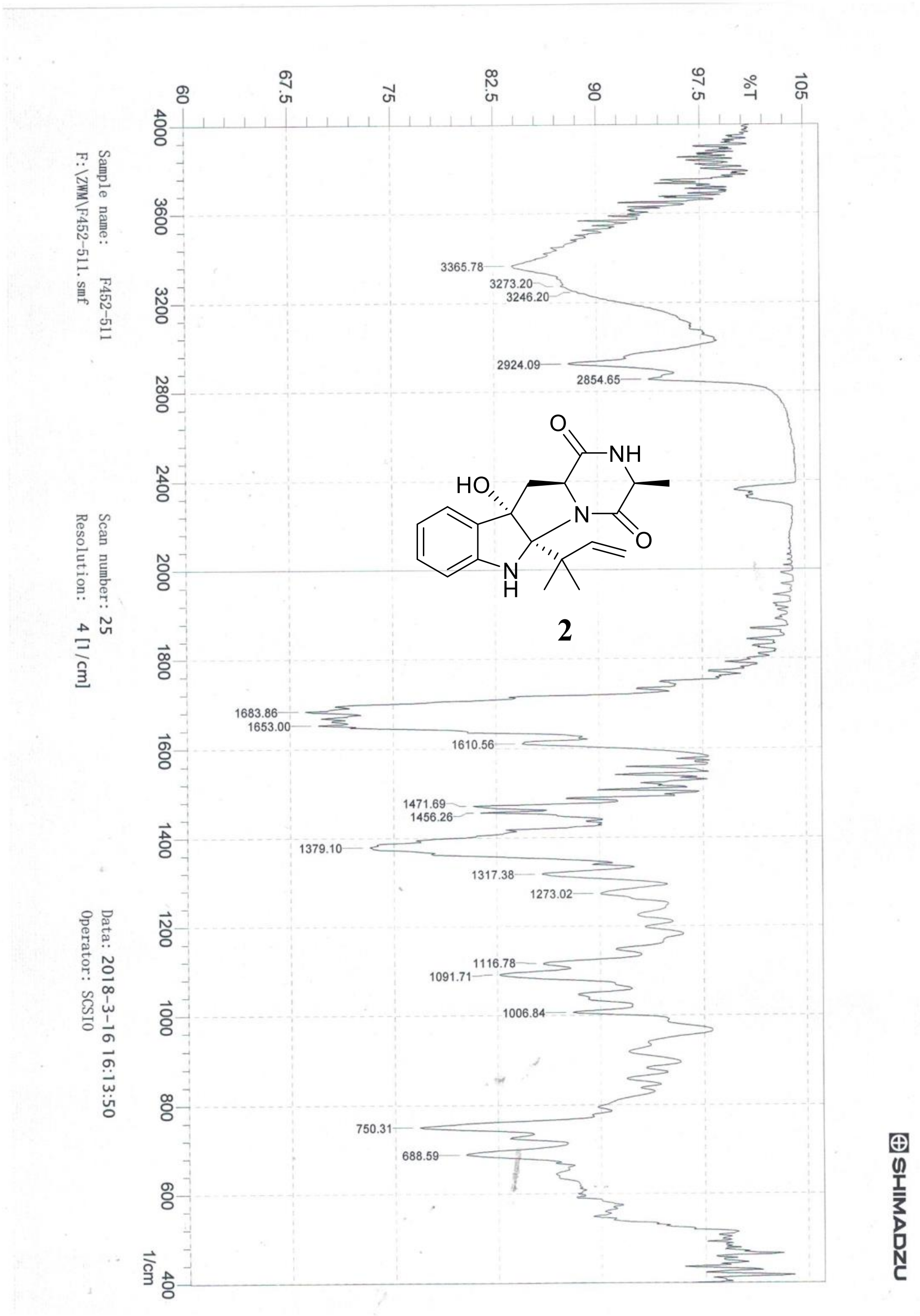

Figure S20. The UV spectrum of eurotiumin B (**2**).

光谱峰值检测报告

2018-01-04 17:46:30

数据集: F452-51-2 - RawData

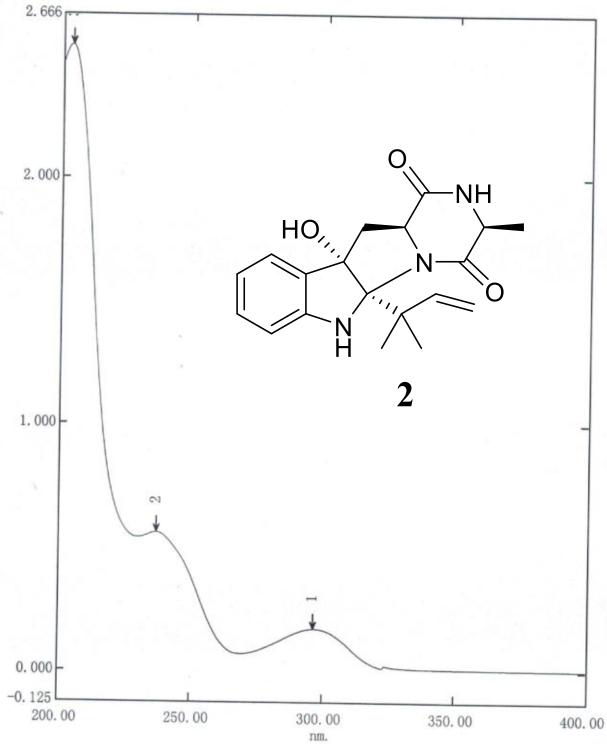

[测定属性]
波长范围 (nm.): 200.00 到 400.00
扫描速度: 中速
采样间隔: 0.2
自动采样间隔: 启用
扫描模式: 单个

[仪器属性]
仪器类型: UV-2600 系列
测定方式: 吸收值
狭缝宽: 2.0
积分时间: 0.1 秒.
光源转换波长: 323.0 nm
检测器单元: 直接
S/R 转换: 标准
阶梯校正: OFF

[附件属性]
附件: 无

[数据处理参数]
阈值: 0.0100000
点: 4
内插: 停用
平均: 停用

[样品准备属性]
重量:
体积:
稀释:
光程长:
附加信息:

No.	P/V	波长(nm)	吸收值	描述
1	⬆	296.40	0.168	
2	⬆	237.00	0.561	
3	⬆	203.60	2.539	

页 1/1

Figure S21. The ^{1}H NMR spectrum of eurotiumin C (**3**) in DMSO-d_6.

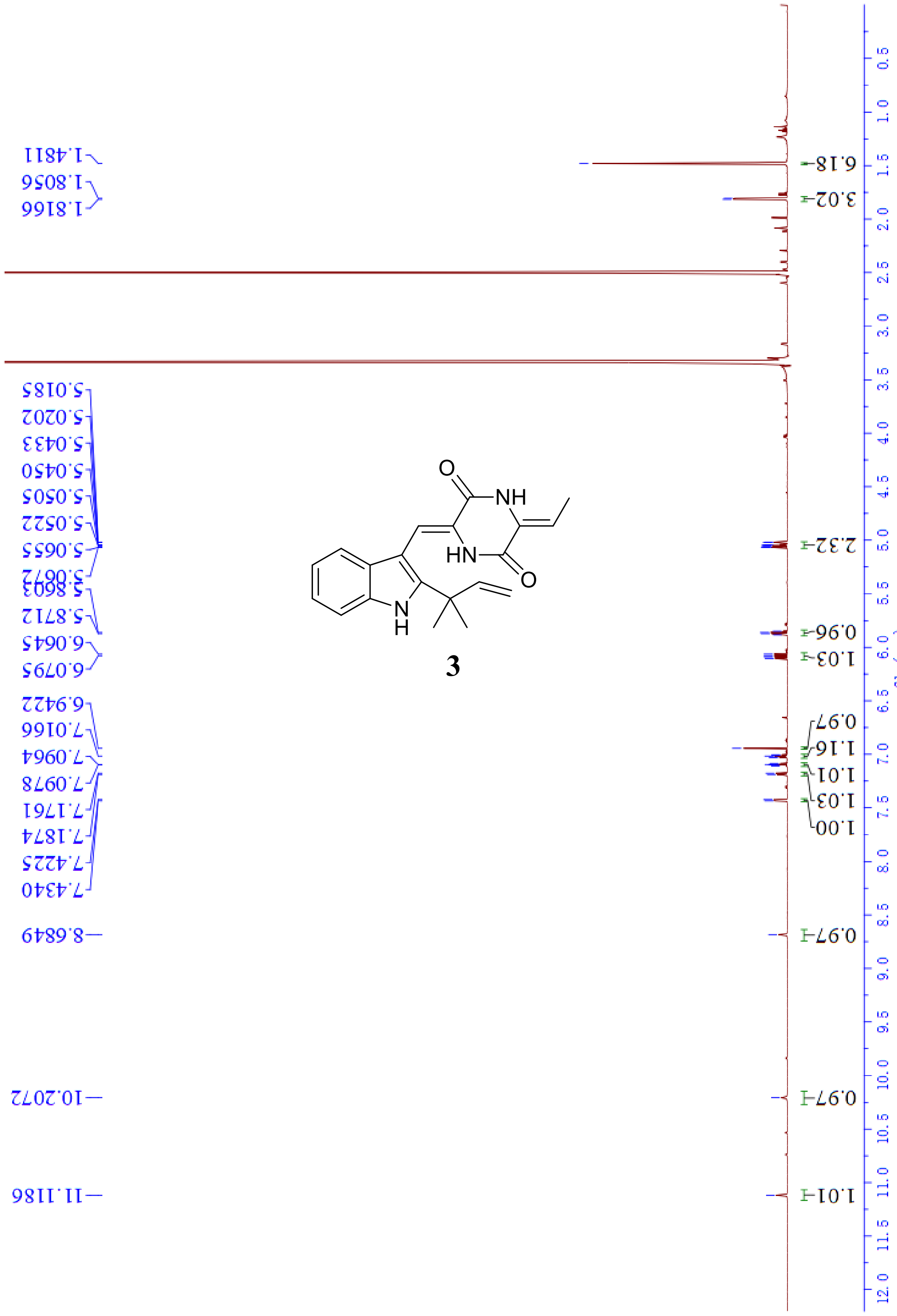

Figure S22. The ^{13}C NMR spectrum of eurotiumin C (**3**) in DMSO-d_6.

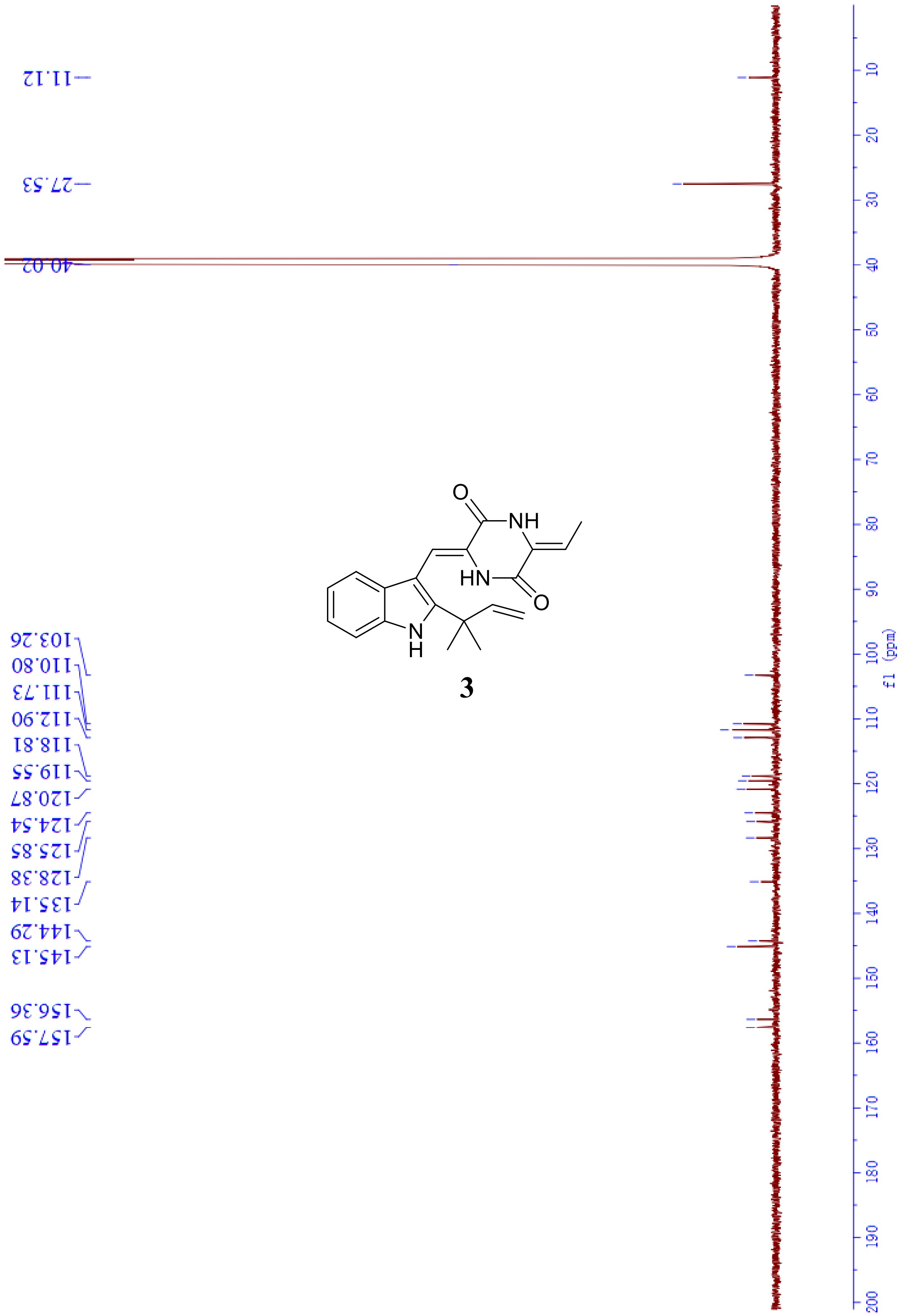

Figure S23. The HSQC spectrum of eurotiumin C (**3**) in DMSO-d_6.

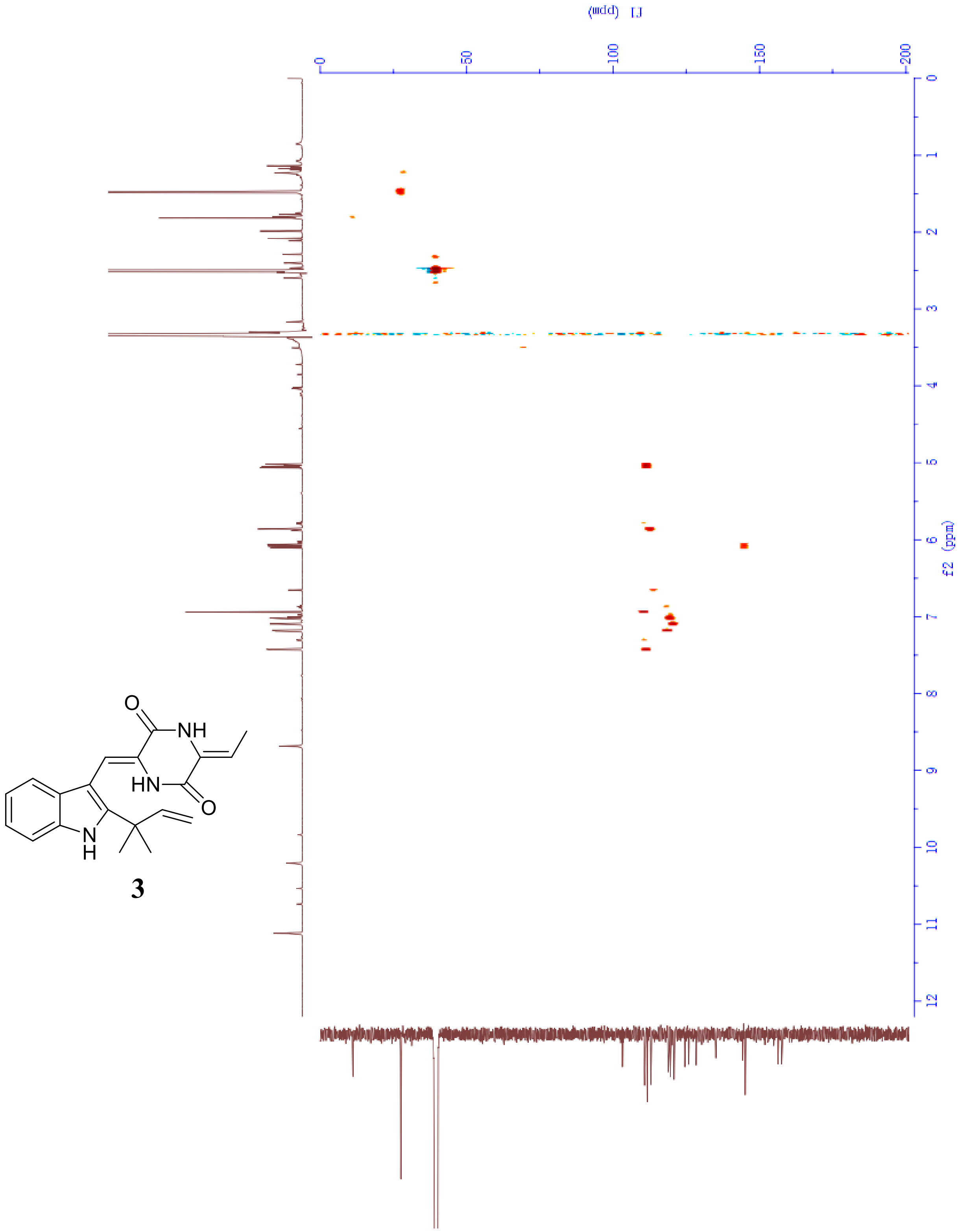

3

Figure S24. The HMBC spectrum of eurotiumin C (**3**) in DMSO-d_6.

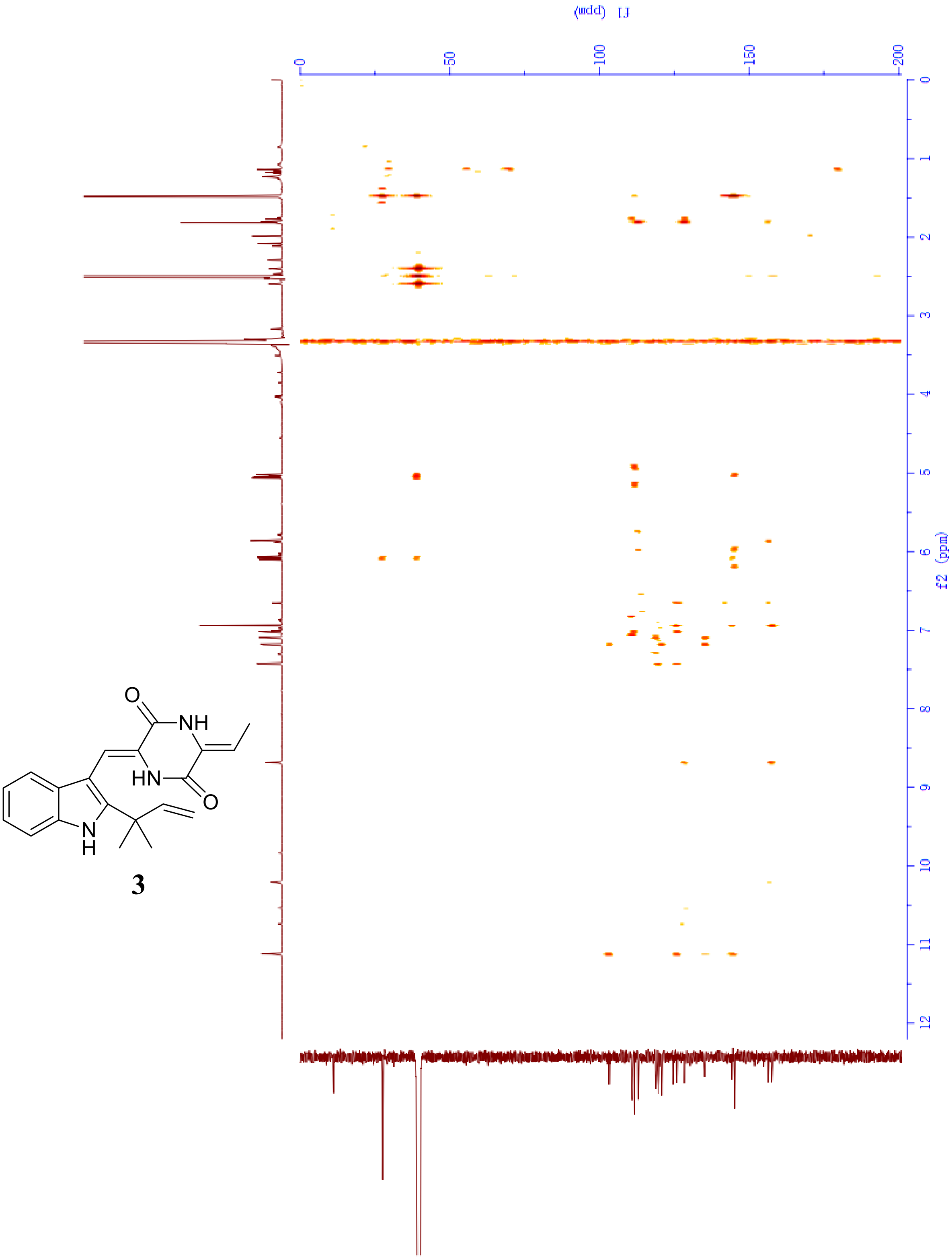

Figure S25. The ^{1}H–^{1}H COSY spectrum of eurotiumin C (**3**) in DMSO-d_6.

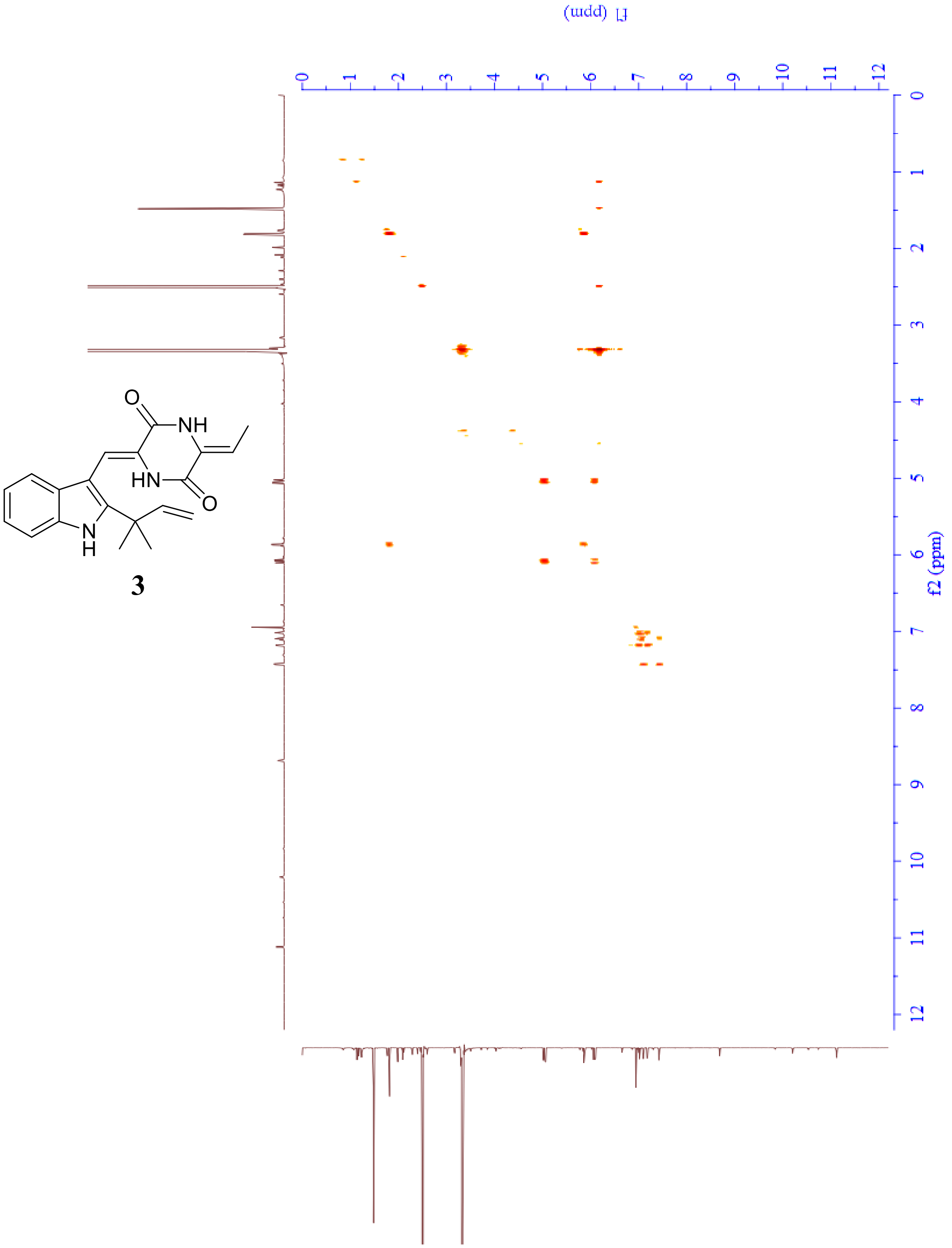

Figure S26. The NOESY spectrum of eurotiumin C (**3**) in DMSO-d_6.

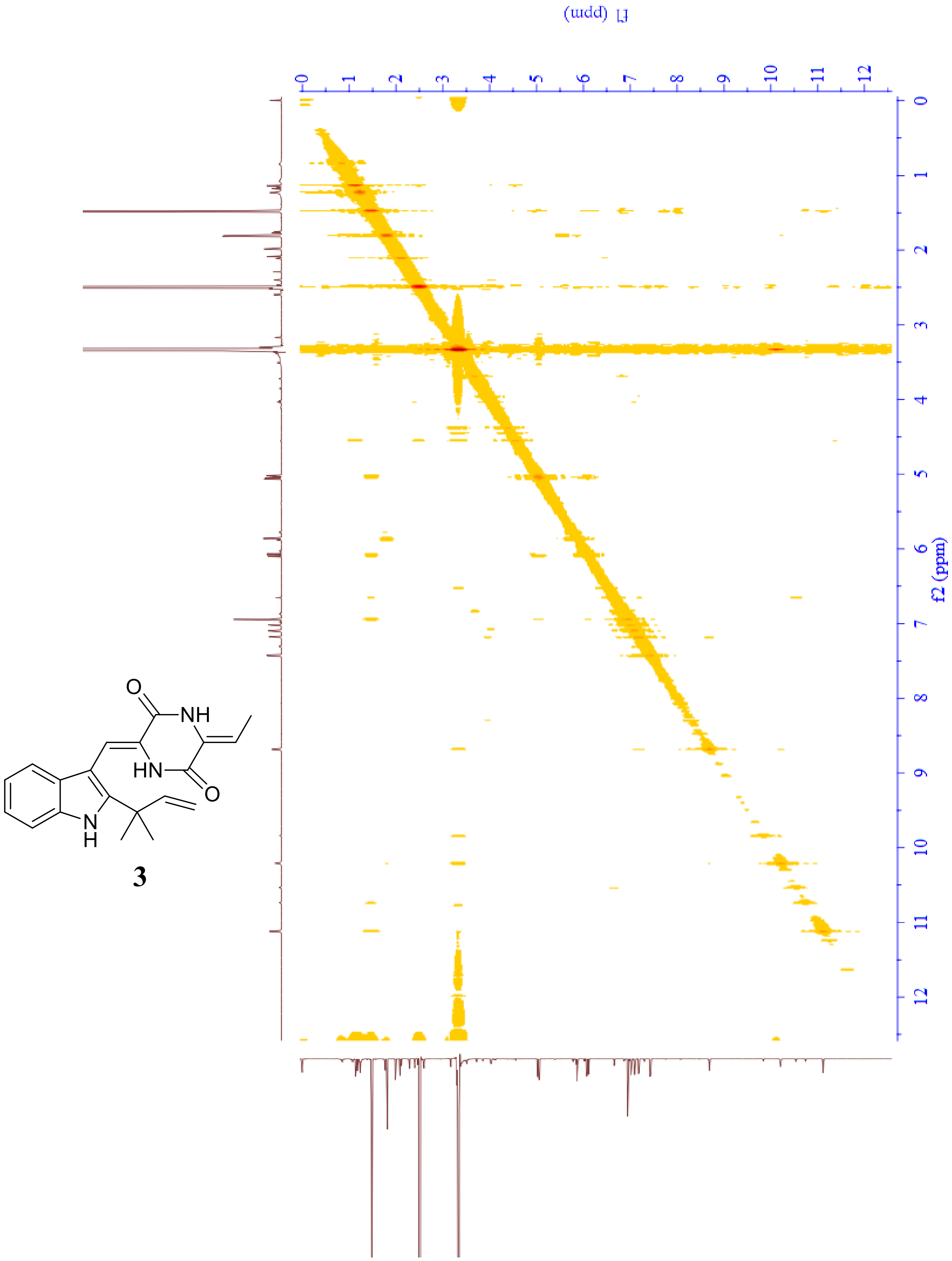

Figure S27. The HRESIMS spectrum of eurotiumin C (**3**).

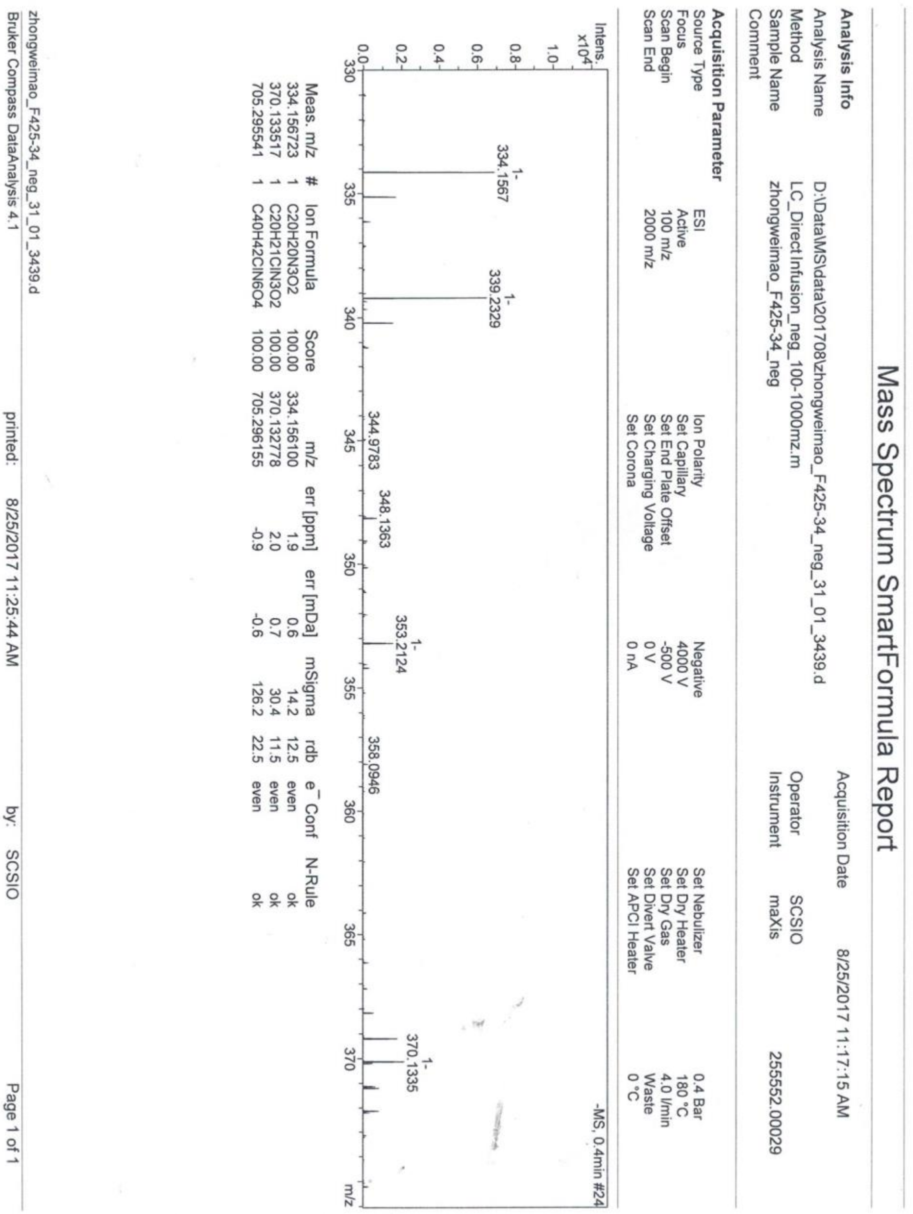

Meas. m/z	#	Ion Formula	Score	m/z	err [ppm]	err [mDa]	mSigma	rdb	e⁻ Conf	N-Rule
334.156723	1	C20H20N3O2	100.00	334.156100	1.9	0.6	14.2	12.5	even	ok
370.133517	1	C20H21ClN3O2	100.00	370.132778	2.0	0.7	30.4	11.5	even	ok
705.295541	1	C40H42ClN6O4	100.00	705.296155	-0.9	-0.6	126.2	22.5	even	ok

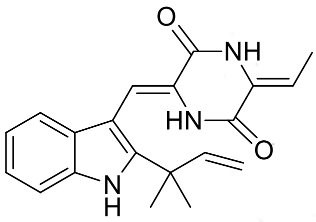

3

Figure S28. The IR spectrum of eurotiumin C (**3**).

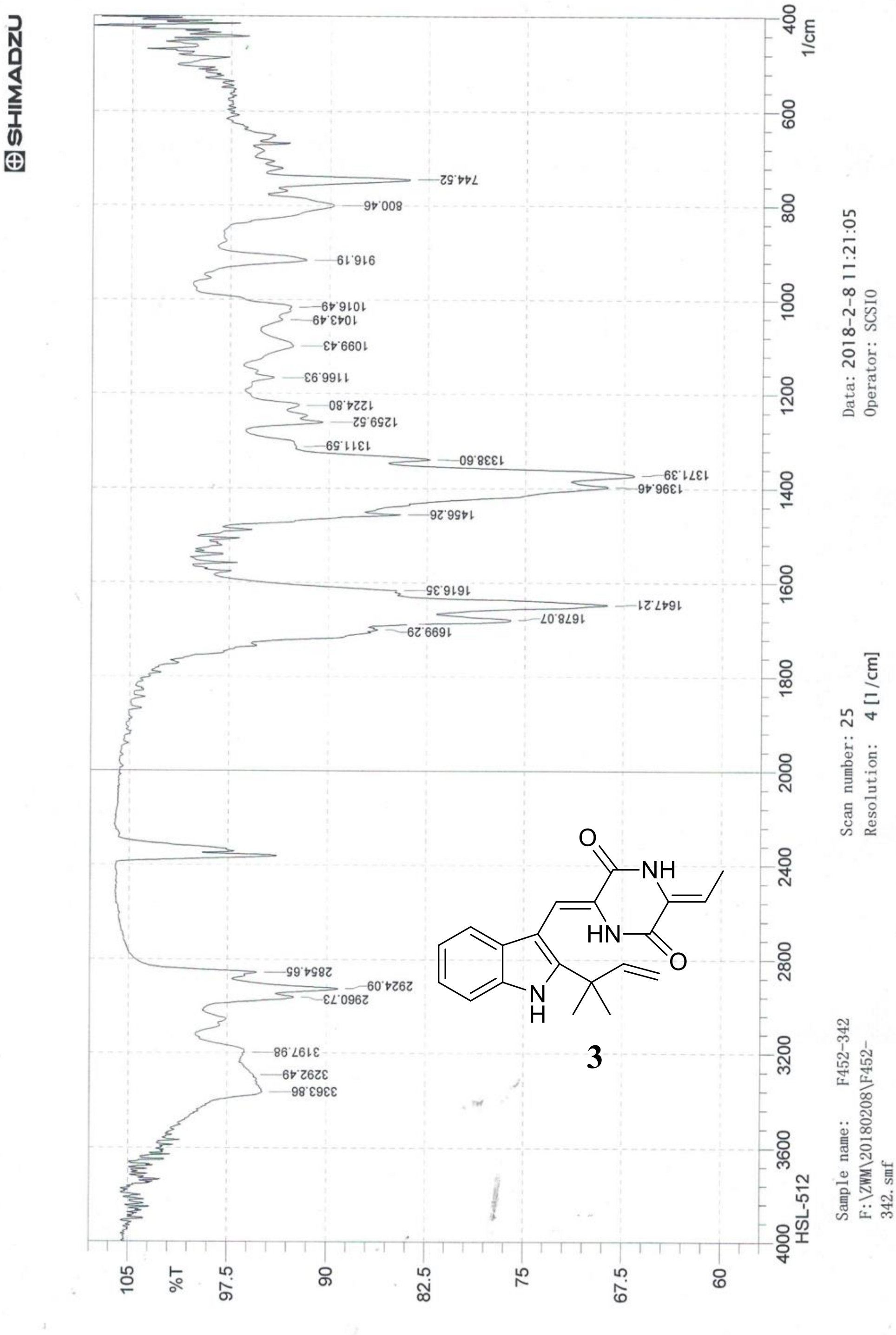

Figure S29. The UV spectrum of eurotiumin C (**3**).

光谱峰值检测报告

2018-02-06 17:19:07

数据集: F452-34-2 - RawData

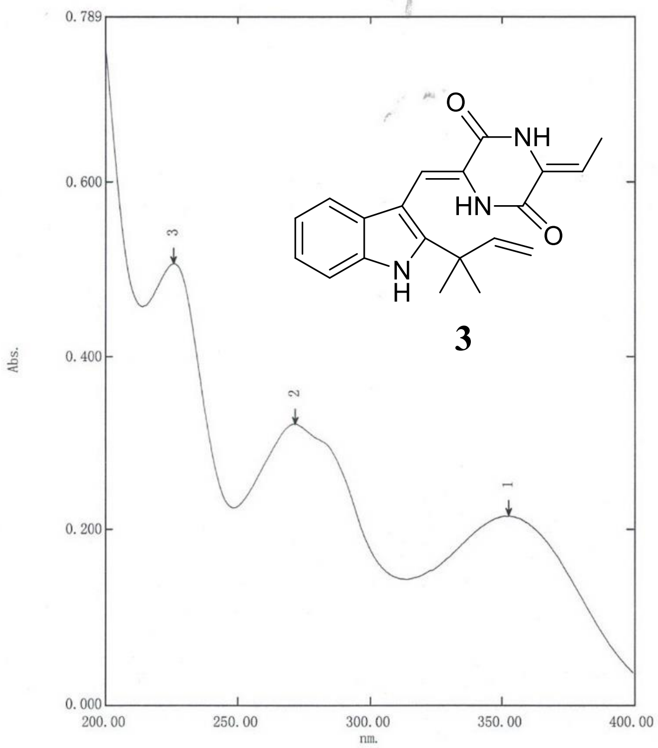

[测定属性]
波长范围 (nm.): 200.00 到 400.00
扫描速度: 中速
采样间隔: 0.2
自动采样间隔: 启用
扫描模式: 单个

[仪器属性]
仪器类型: UV-2600 系列
测定方式: 吸收值
狭缝宽: 2.0
积分时间: 0.1 秒.
光源转换波长: 323.0 nm
检测器单元: 直接
S/R 转换: 标准
阶梯校正: OFF

[附件属性]
附件: 无

[数据处理参数]
阈值: 0.0100000
点: 5
内插: 停用
平均: 停用

[样品准备属性]
重量:
体积:
稀释:
光程长:
附加信息:

No.	P/V	波长(nm)	吸收值	描述
1	↑	352.80	0.216	
2	↑	271.80	0.321	
3	↑	225.80	0.506	

页 1 / 1

Figure S30. The ^{1}H NMR spectrum of eurotiumin D (**4**) in CD_3COCD_3.

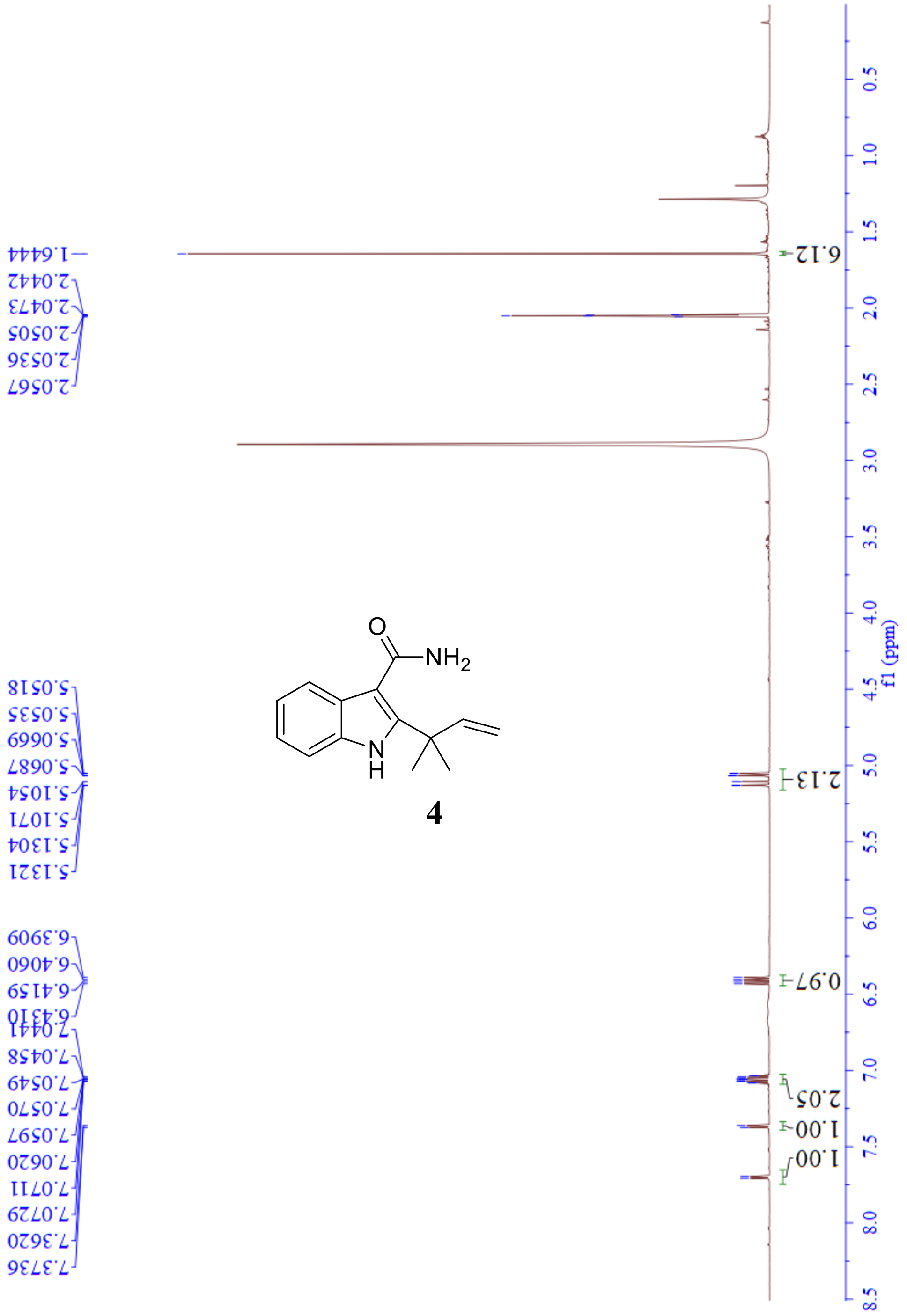

Figure S31. The ^{13}C NMR spectrum of eurotiumin D (**4**) in CD_3COCD_3.

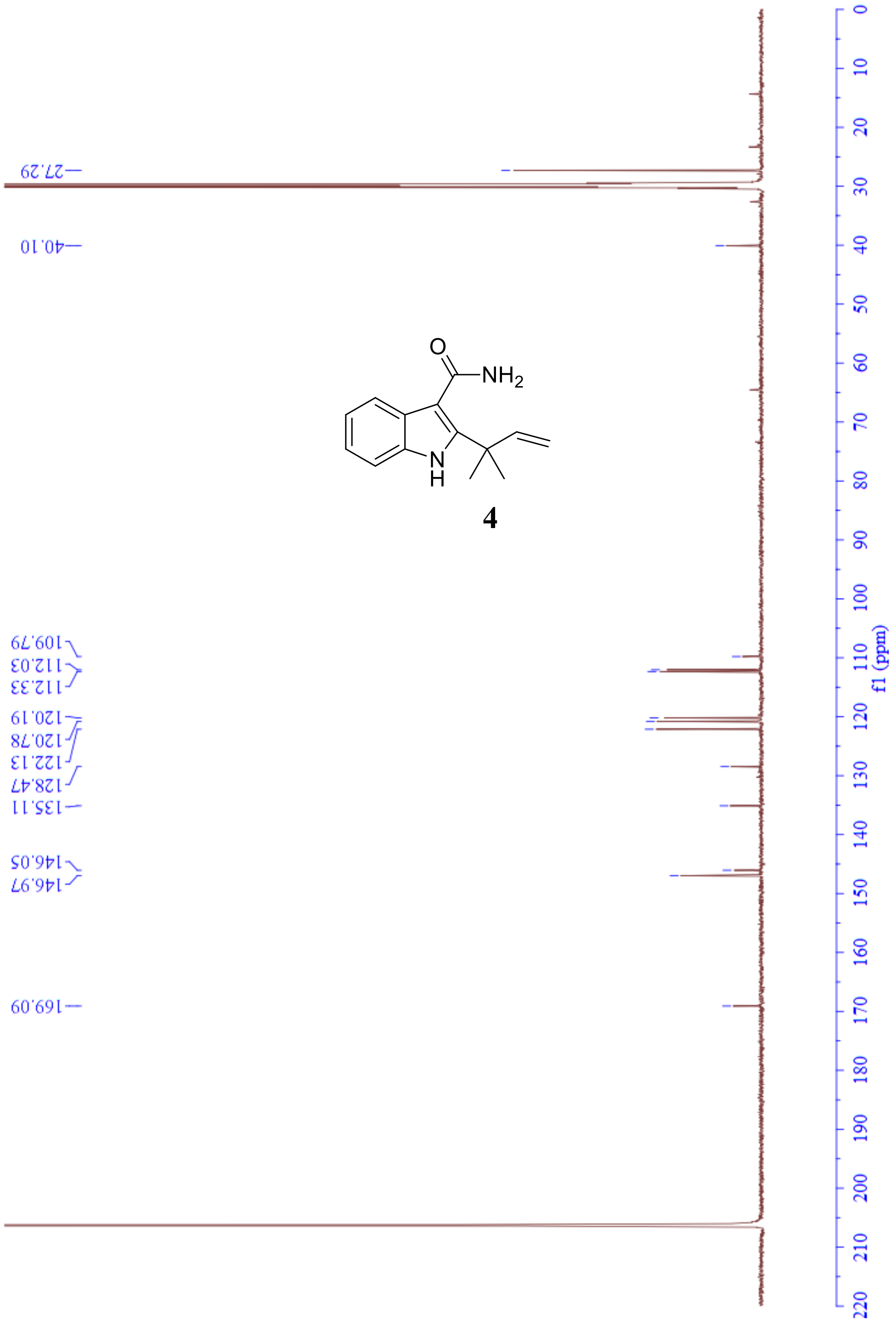

Figure S32. The HSQC spectrum of eurotiumin D (**4**) in CD_3COCD_3.

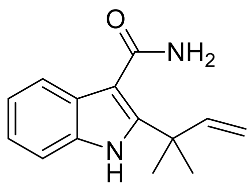

4

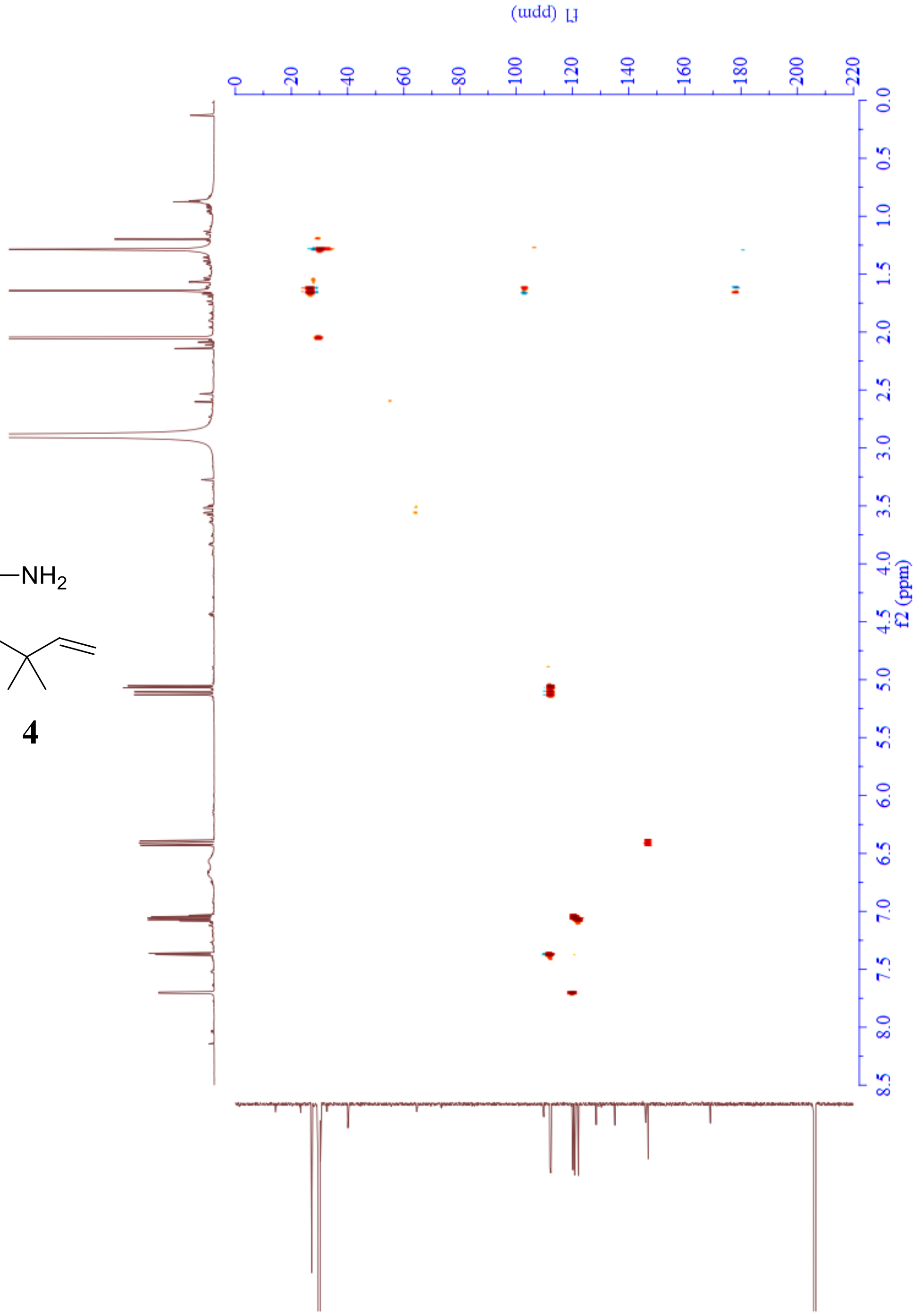

Figure S33. The HMBC spectrum of eurotiumin D (**4**) in CD3COCD3.

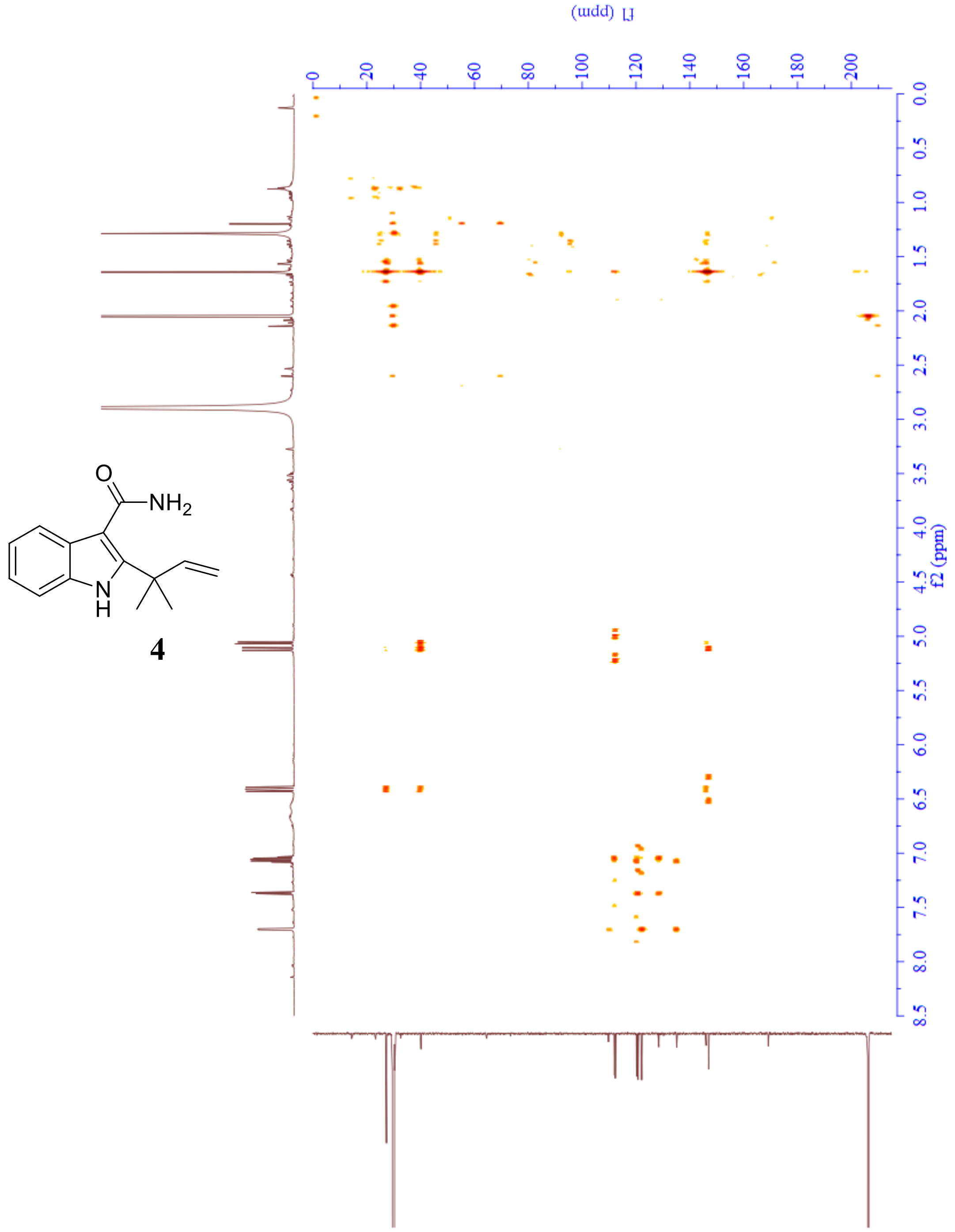

Figure S34. The ^{1}H–^{1}H COSY spectrum of eurotiumin D (**4**) in CD_3COCD_3.

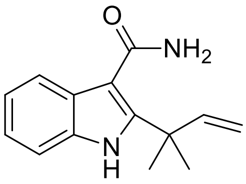

4

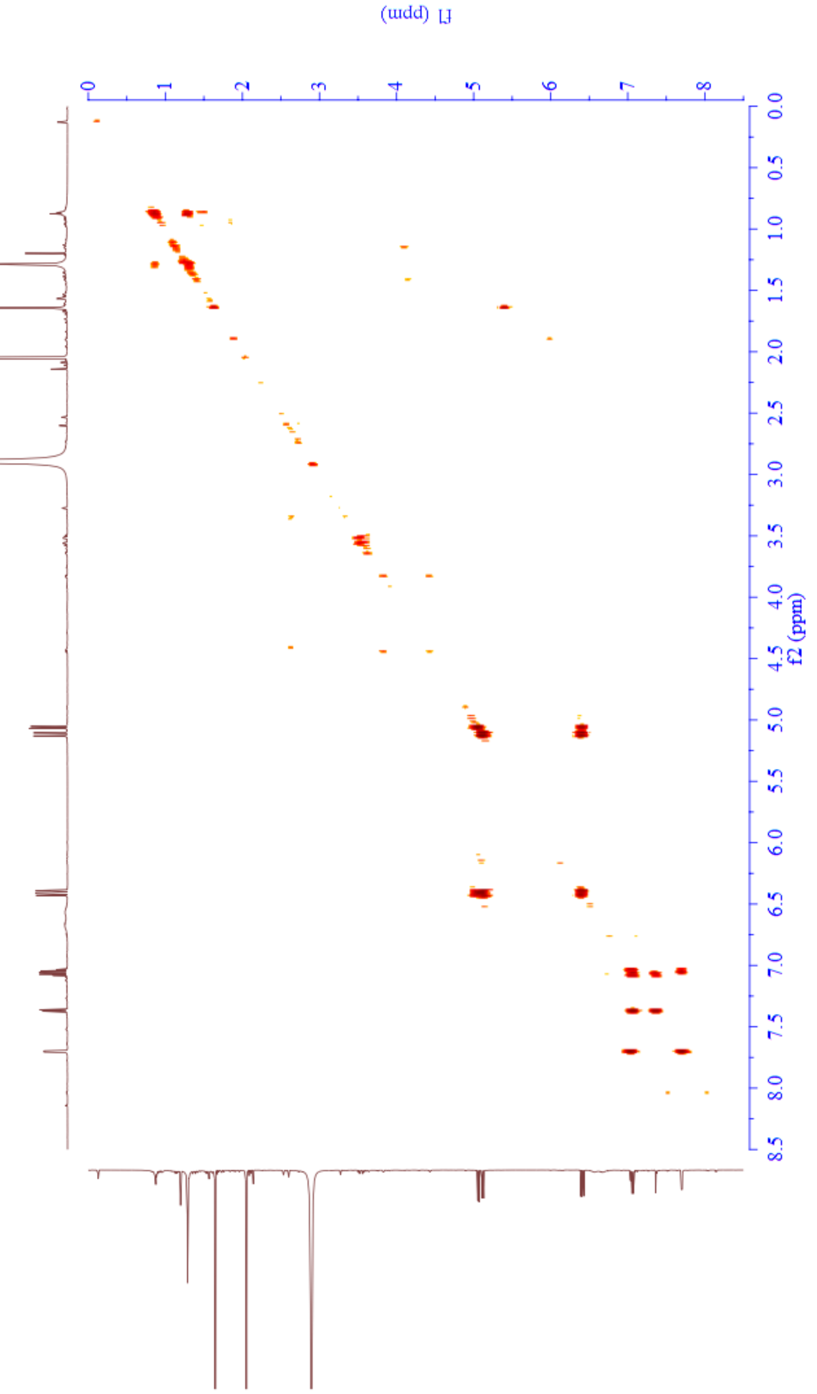

Figure S35. The NOESY spectrum of eurotiumin D (**4**) in CD_3COCD_3.

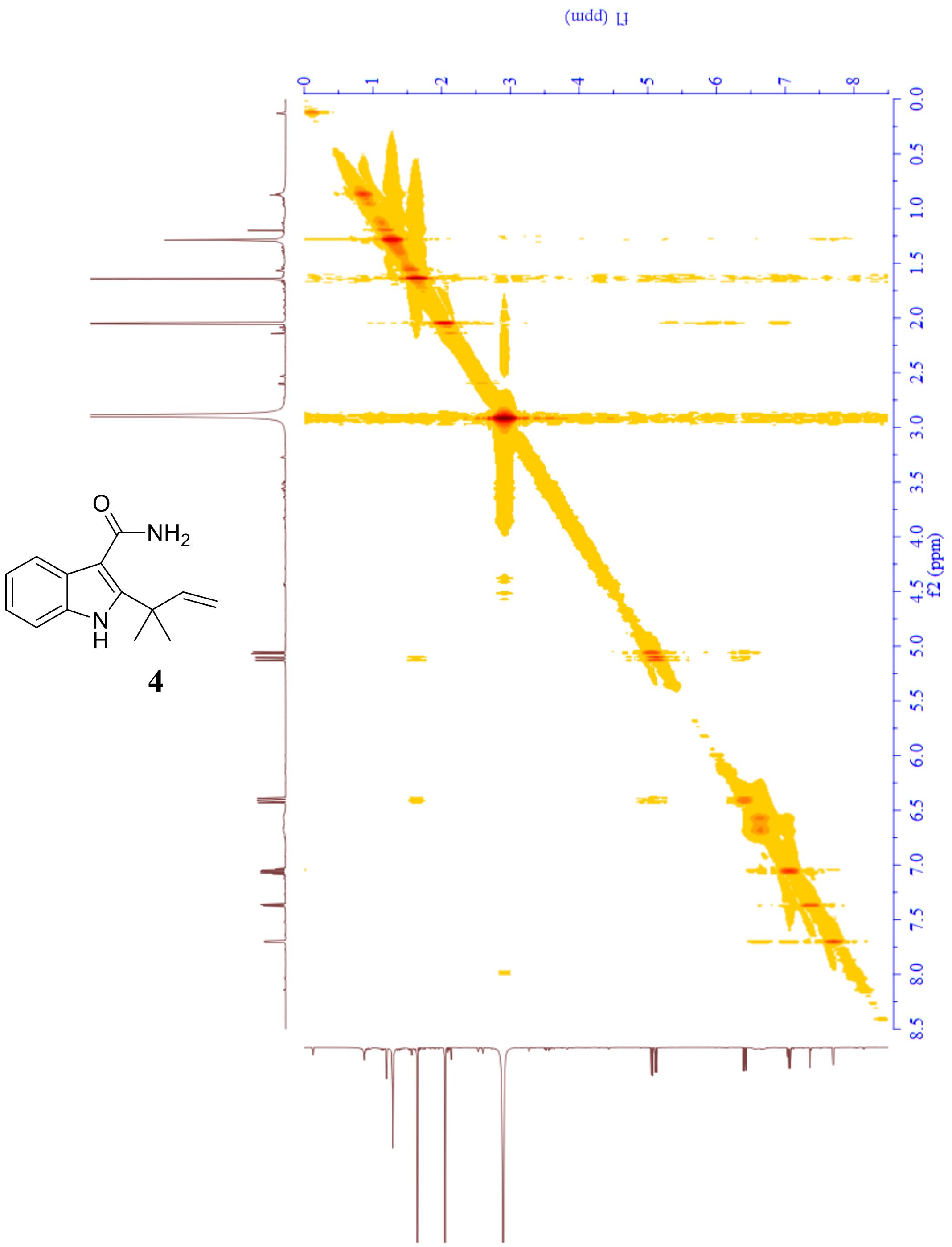

4

Figure S36. The HRESIMS spectrum of eurotiumin D (**4**).

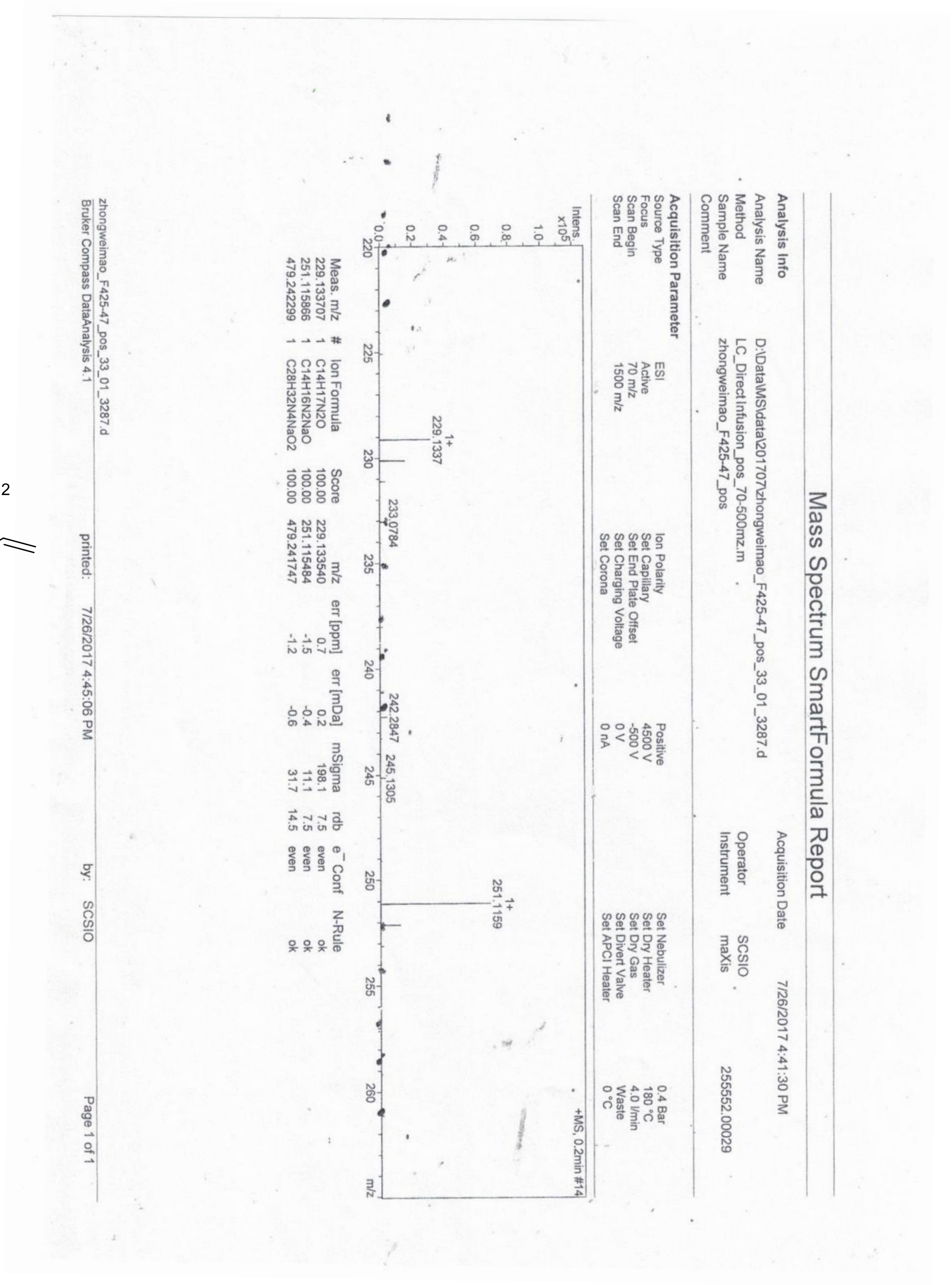

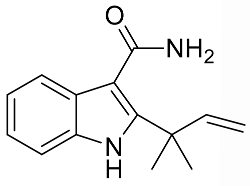

4

Figure S37. The IR spectrum of eurotiumin D (**4**).

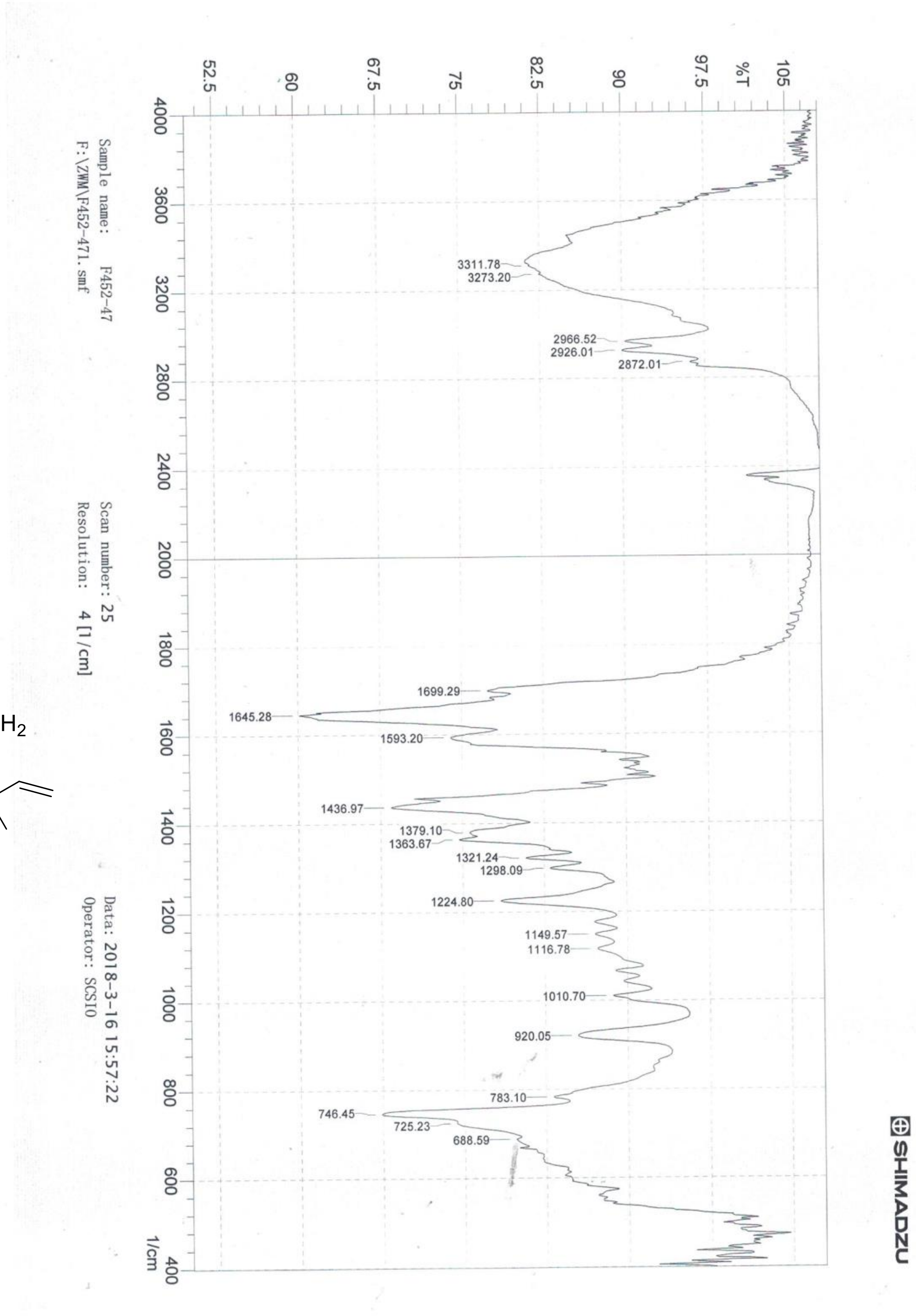

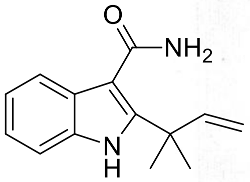

4

Figure S38. The UV spectrum of eurotiumin D (**4**).

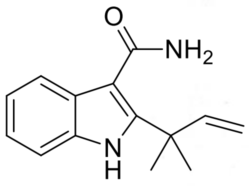

4

光谱峰值检测报告

2018-03-15 18:03:59

数据集: F452-47 - RawData

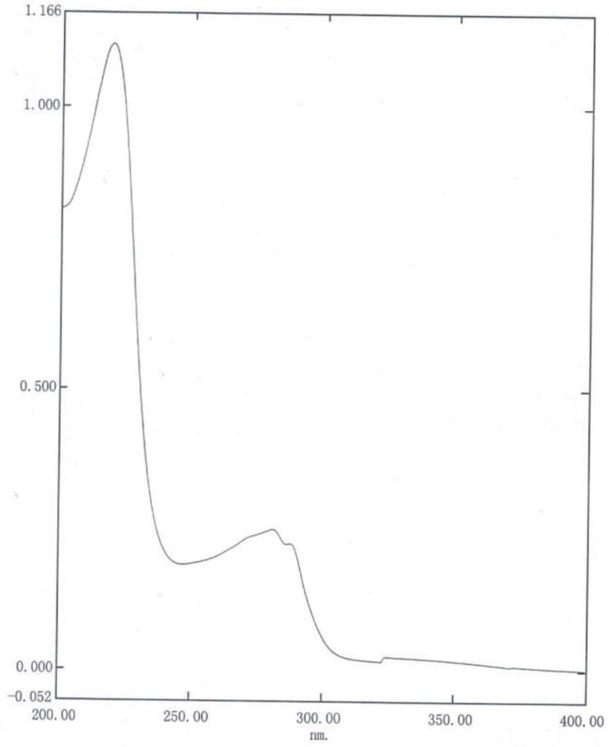

[测定属性]
波长范围 (nm.): 200.00 到 400.00
扫描速度: 中速
采样间隔: 0.2
自动采样间隔: 启用
扫描模式: 单个

[仪器属性]
仪器类型: UV-2600 系列
测定方式: 吸收值
狭缝宽: 2.0
积分时间: 0.1 秒.
光源转换波长: 323.0 nm
检测器单元: 直接
S/R 转换: 标准
阶梯校正: OFF

[附件属性]
附件: 无

[数据处理参数]
阈值: 0.0100000
点: 5
内插: 停用
平均: 停用

[样品准备属性]
重量:
体积:
稀释:
光程长:
附加信息:

No.	P/V	波长(nm)	吸收值	描述
1	↑	287.60	0.226	
2	↑	280.80	0.251	
3	↑	219.00	1.111	

页 1 / 1

Figure S39. The ¹H NMR spectrum of eurotiumin E (**14**) in CD_3COCD_3.

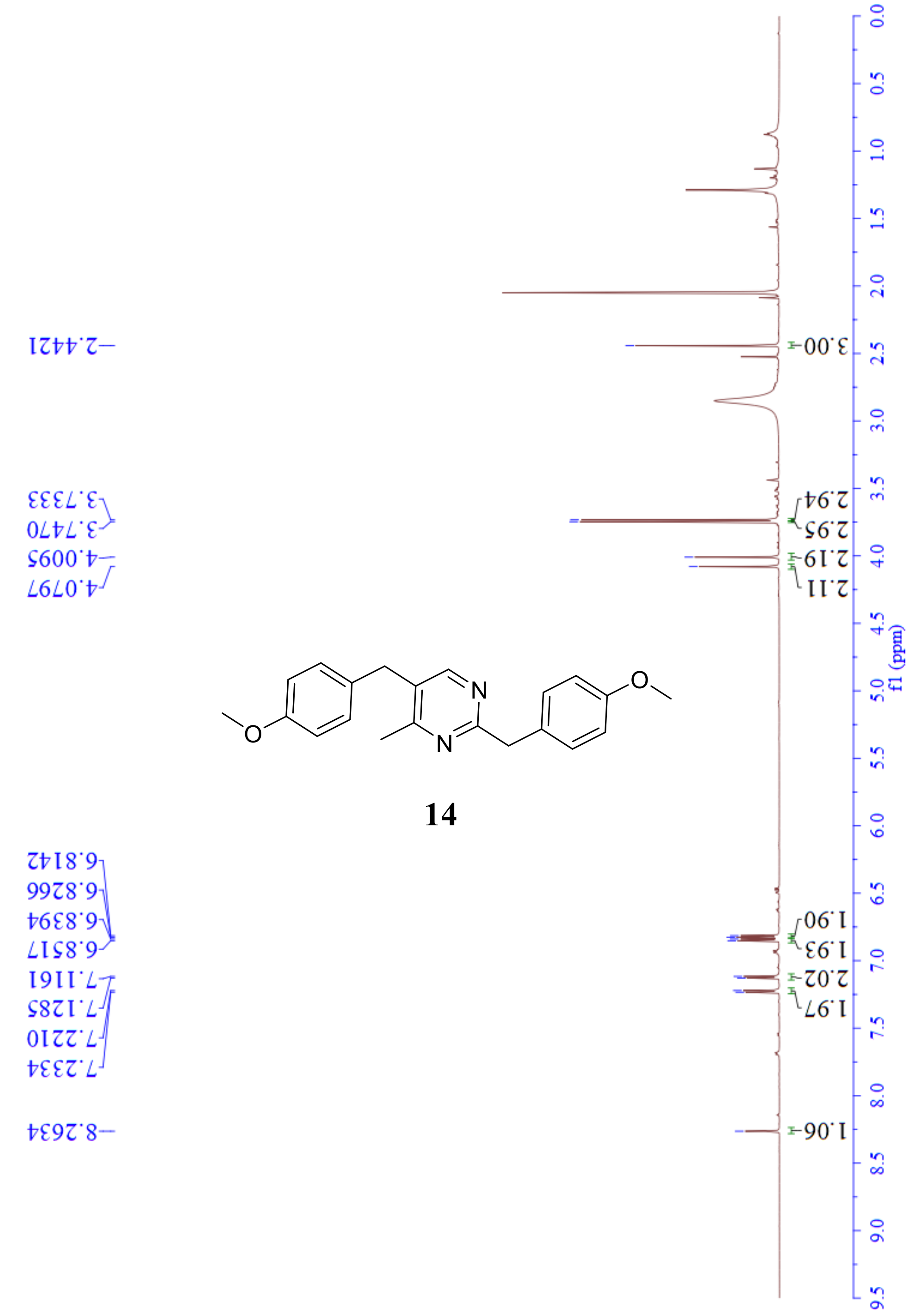

14

Figure S40. The ^{13}C NMR spectrum of eurotiumin E (**14**) in CD_3COCD_3.

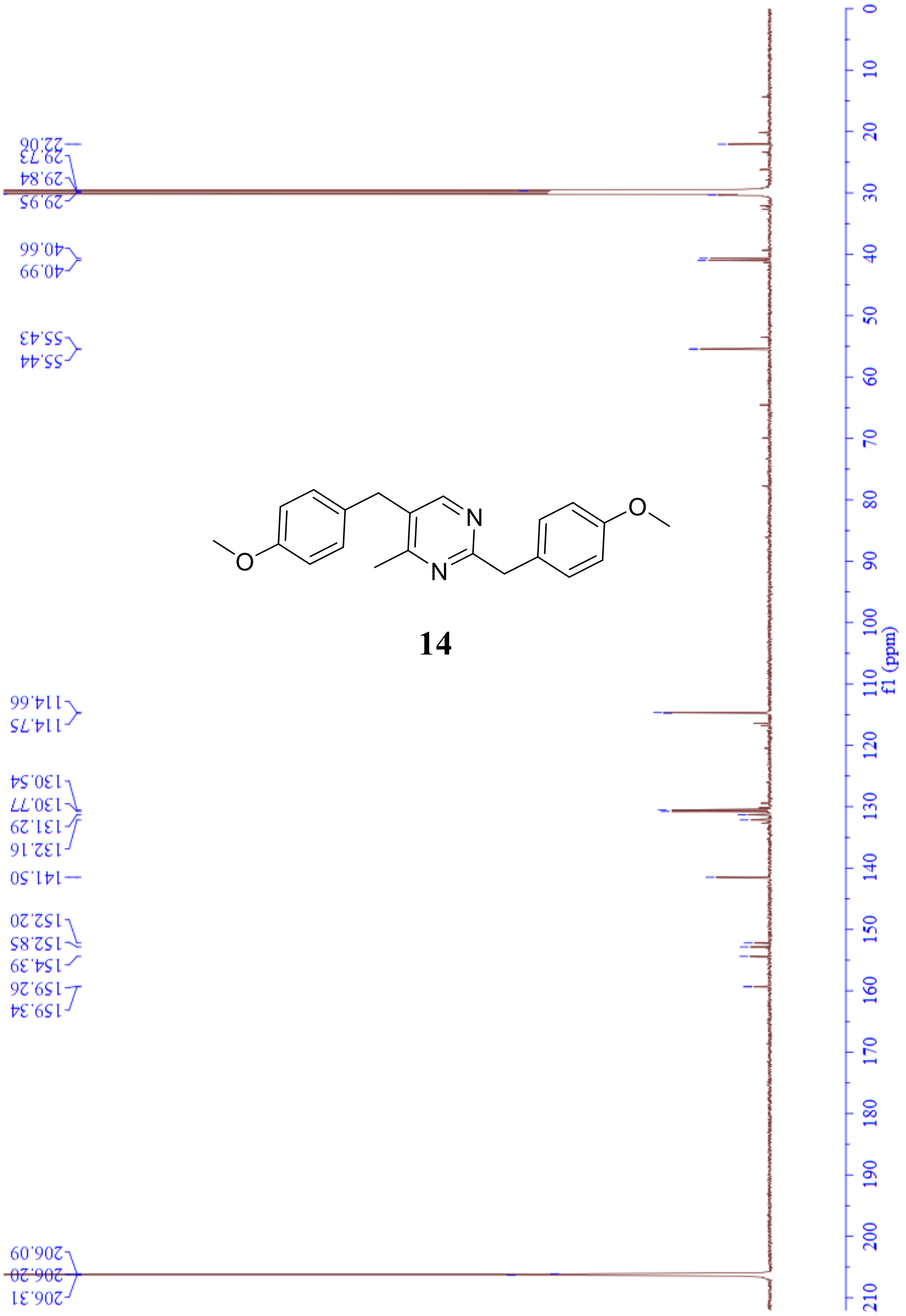

Figure S41. The HSQC spectrum of eurotiumin E (14) in CD_3COCD_3.

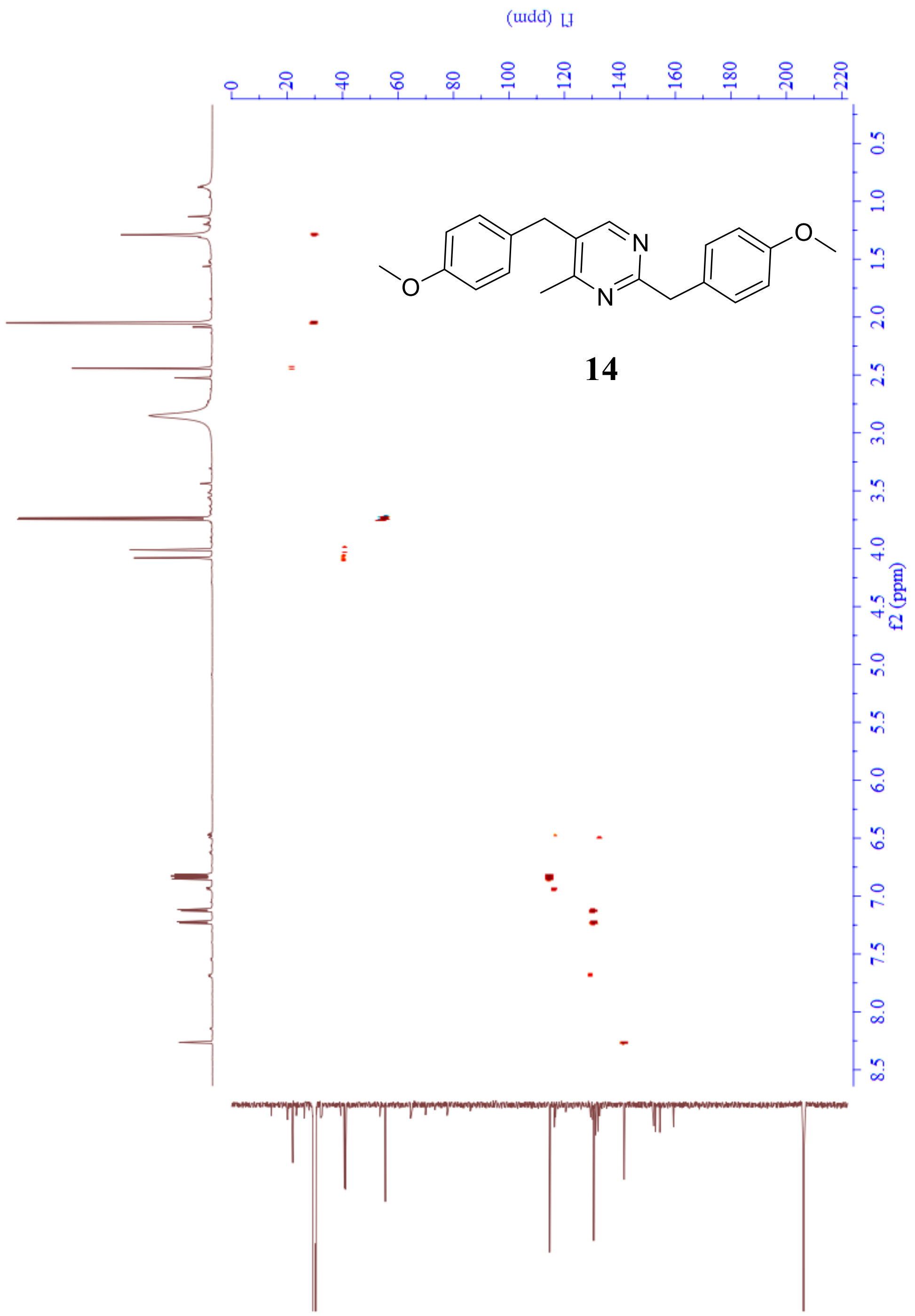

Figure S42. The HMBC spectrum of eurotiumin E (**14**) in CD_3COCD_3.

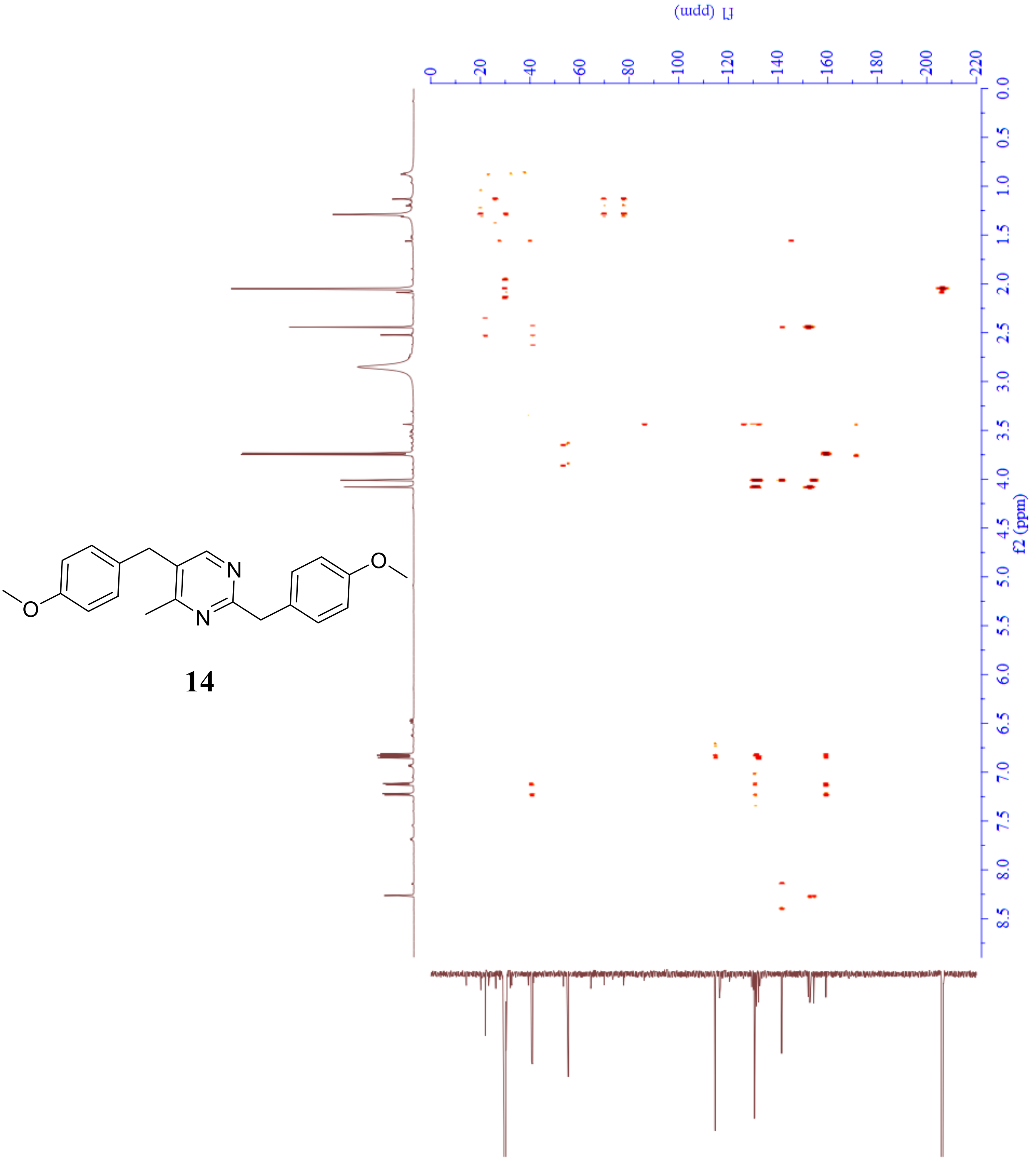

Figure S43. The ^1H–^1H COSY spectrum of eurotiumin E (**14**) in CD_3COCD_3.

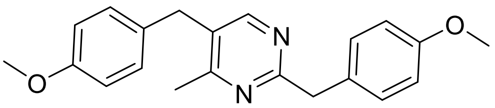

14

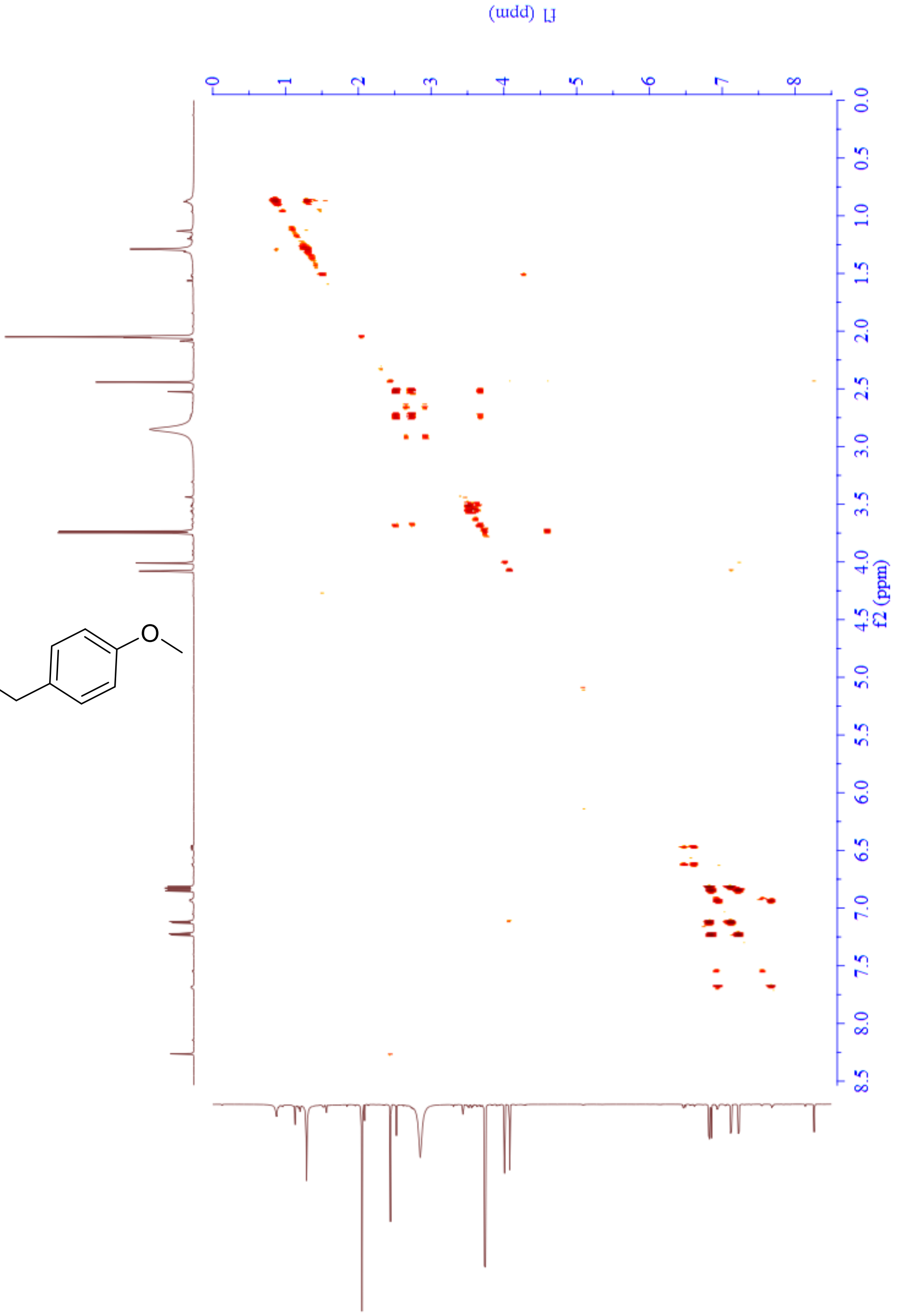

Figure S44. The HRESIMS spectrum of eurotiumin E (**14**).

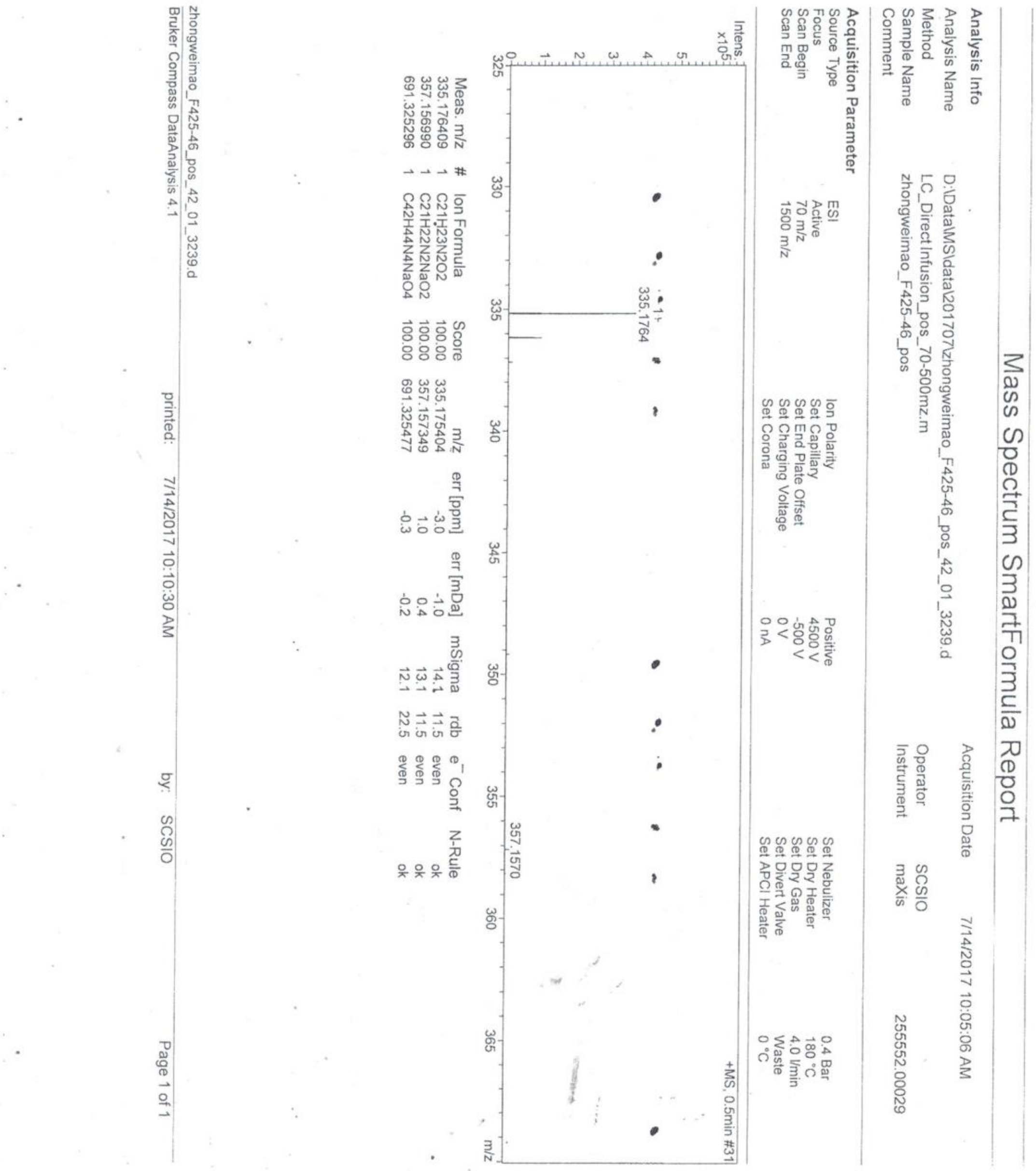

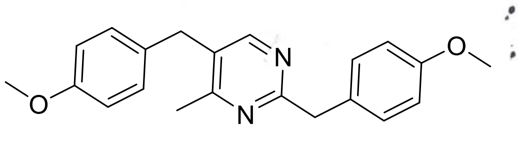

14

Figure S45. The UV spectrum of eurotiumin E (**14**).

光谱峰值检测报告

2018-03-15 17:57:40

数据集: F452-46-1 - RawData

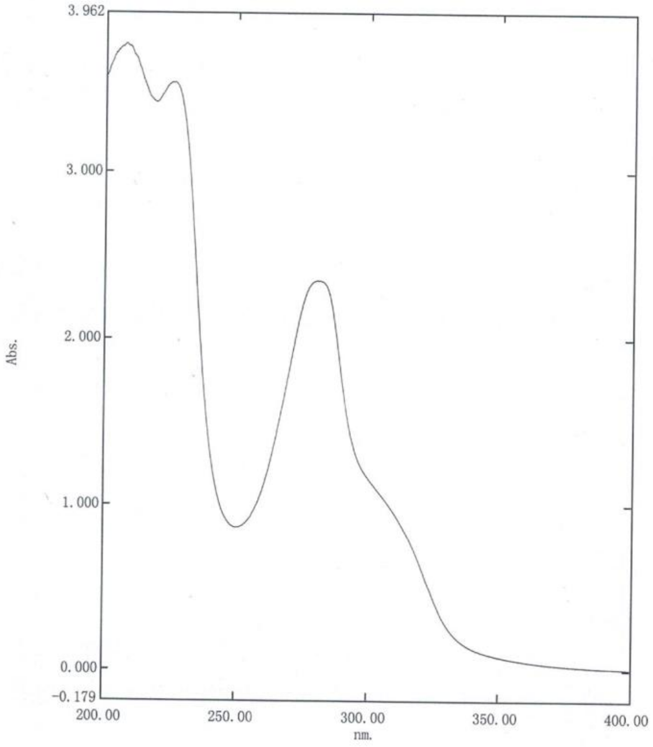

[测定属性]
波长范围 (nm.): 200. 00 到 400. 00
扫描速度: 中速
采样间隔: 0. 2
自动采样间隔: 启用
扫描模式: 单个

[仪器属性]
仪器类型: UV-2600 系列
测定方式: 吸收值
狭缝宽: 2. 0
积分时间: 0. 1 秒.
光源转换波长: 323. 0 nm
检测器单元: 直接
S/R 转换: 标准
阶梯校正: OFF

[附件属性]
附件: 无

[数据处理参数]
阈值: 0. 0100000
点: 5
内插: 停用
平均: 停用

[样品准备属性]
重量:
体积:
稀释:
光程长:
附加信息:

No.	P/V	波长(nm)	吸收值	描述
1	↑	280. 40	2. 346	
2	↑	226. 20	3. 541	
3	↑	207. 60	3. 773	

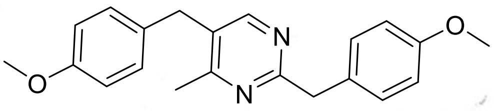

14

页 1 / 1

MS and NMR data of compounds 5–13

Compound **5**: yellow solid, positive HRESIMS *m/z* 482.2775 [M + Na]$^+$ (calcd for $C_{29}H_{37}N_3NaO_2$, 482.2778). ^{13}C NMR（acetone-d_6, 175 MHz）, δ_C: 144.4 (C-2), 104.8 (C-3), 127.6 (C-3a), 110.9 (C-4), 133.4 (C-5), 125.4 (C-6), 126.4 (C-7), 134.6 (C-7a), 116.8 (C-8), 125.1 (C-9), 160.3 (C-10), 52.3 (C-12), 166.7 (C-13), 40.1 (C-15), 146.1 (C-16), 112.4 (C-17), 27.9 (C-18/19), 20.8 (C-20), 35.1 (C-21), 123.0 (C-22), 131.9 (C-23), 25.9 (C-24), 17.9 (C-25), 32.6 (C-26), 123.4 (C-27), 133.2 (C-28), 25.9 (C-29), 17.9 (C-30).

Compound **6**: yellow solid, positive ESIMS *m/z* 414.4 [M + Na]$^+$. ^{13}C NMR（acetone-d_6, 175 MHz）, δ_C: 144.3 (C-2), 105.0 (C-3), 125.4 (C-3a), 117.7 (C-4), 121.2 (C-5), 123.2 (C-6), 126.8 (C-7), 134.9 (C-7a), 110.7 (C-8), 127.4 (C-9), 160.2 (C-10), 52.2 (C-12), 166.9 (C-13), 40.1 (C-15), 146.1 (C-16), 112.4 (C-17), 27.9 (C-18/19), 20.7 (C-20), 28.9 (C-21), 123.2 (C-22), 133.4 (C-23), 25.9 (C-24), 17.9 (C-25).

Compound **7**: yellow solid, positive HRESIMS *m/z* 392.2344 [M + H]$^+$ (calcd for $C_{24}H_{30}N_3O_2$, 392.2333). ^{13}C NMR（acetone-d_6, 125 MHz）, δ_C: 145.2 (C-2), 103.8 (C-3), 127.2 (C-3a), 118.6 (C-4), 134.0 (C-5), 123.1 (C-6), 112.3 (C-7), 134.7 (C-7a), 111.8 (C-8), 125.5 (C-9), 160.8 (C-10), 52.2 (C-12), 167.1 (C-13), 40.0 (C-15), 145.9 (C-16), 112.2 (C-17), 27.9 (C-18/19), 20.8 (C-20), 35.0 (C-21), 125.5 (C-22), 131.9 (C-23), 25.8 (C-24), 17.8 (C-25).

Compound **8**: red solid, positive HRESIMS *m/z* 460.2967 [M + H]$^+$ (calcd for $C_{29}H_{38}N_3O_2$, 460.2959). ^{13}C NMR（acetone-d_6, 175 MHz）, δ_C: 142.7 (C-2), 103.0 (C-3), 127.6 (C-3a), 131.8 (C-4), 131.7 (C-5), 124.3 (C-6), 110.0 (C-7), 135.5 (C-7a), 113.4 (C-8), 130.2 (C-9), 159.3 (C-10), 52.2 (C-12), 166.8 (C-13), 39.8 (C-15), 146.4 (C-16), 111.7 (C-17), 27.5 (C-18/19), 21.2 (C-20), 28.7 (C-21), 127.6 (C-22), 131.0 (C-23), 25.8 (C-24), 18.2 (C-25), 31.9 (C-26), 127.6 (C-27), 131.1 (C-28), 25.9 (C-29), 17.9 (C-30).

Compound **9**: yellow solid, negative ESIMS *m/z* 338.2 [M - H]$^-$. ^{13}C NMR（dimethyl sulfoxide-d_6, 175 MHz）, δ_C: 144.2 (C-2), 103.8 (C-3), 126.2 (C-3a), 111.5 (C-4), 119.5 (C-5), 120.7 (C-6), 119.3 (C-7), 135.1 (C-7a), 111.3 (C-8), 125.1 (C-9), 165.8 (C-10), 79.1 (C-12), 161.4 (C-13), 39.1 (C-15), 145.2 (C-16), 111.7 (C-17), 27.8 (C-18), 27.5 (C-19), 24.7 (C-20).

Compound **10**: yellow oil, positive HRESIMS *m/z* 322.1560 [M + H]$^+$ (calcd for $C_{19}H_{20}N_3O_2$, 322.1550). ^{13}C NMR（acetone-d_6, 125 MHz）, δ_C: 145.3 (C-2), 104.2 (C-3), 127.1 (C-3a), 119.7 (C-4), 120.9 (C-5), 122.3 (C-6), 112.5 (C-7), 136.3 (C-7a), 112.2 (C-8), 126.3 (C-9), 157.8 (C-10), 136.1 (C-12), 156.6 (C-13), 40.1 (C-15), 145.9 (C-16), 112.4 (C-17), 27.9 (C-18/19), 100.1 (C-20).

Compound **11**: yellow oil, positive HRESIMS *m/z* 392.1972 [M + H]$^+$ (calcd for $C_{23}H_{26}N_3O_3$,

392.1969). ^{13}C NMR（acetone-d_6, 125 MHz）, δ_C: 146.9 (C-2), 104.3 (C-3), 127.3 (C-3a), 119.2 (C-4), 134.8 (C-5), 123.5 (C-6), 112.5 (C-7), 135.0 (C-7a), 117.3 (C-8), 124.6 (C-9), 157.5 (C-10), 152.6 (C-12), 160.6 (C-13), 40.2 (C-15), 145.7 (C-16), 112.7 (C-17), 28.1 (C-18/19), 35.2 (C-21), 125.4 (C-22), 131.9 (C-23), 25.8 (C-24), 17.9 (C-25).

Compound **12**: yellow oil, positive HRESIMS *m*/*z* 462.3121 $[M + H]^+$ (calcd for $C_{29}H_{40}N_3O_2$, 462.3115). ^{13}C NMR（acetone-d_6, 125 MHz）, δ_C: 141.5 (C-2), 104.2 (C-3), 129.1 (C-3a), 115.2 (C-4), 134.0 (C-5), 123.0 (C-6), 123.5 (C-7), 132.4 (C-7a), 29.7 (C-8), 54.7 (C-9), 168.8 (C-10), 50.9 (C-12), 168.0 (C-13), 39.1 (C-15), 145.9 (C-16), 112.5 (C-17), 28.1 (C-18), 28.0 (C-19), 20.0 (C-20), 34.7 (C-21), 124.6 (C-22), 131.7 (C-23), 25.8 (C-24), 18.0 (C-25), 31.5 (C-26), 123.0 (C-27), 133.0 (C-28), 25.9 (C-29), 18.0 (C-30).

Compound **13**: white solid, positive ESIMS *m*/*z* 245 $[M + H]^+$. ^{13}C NMR（acetone-d_6, 125 MHz）, δ_C: 166.9 (C-1), 45.9 (C-3), 22.8 (C-4), 29.3 (C-5), 60.0 (C-6), 170.9 (C-7), 57.6 (C-9), 38.1 (C-10), 137.4 (C-1′), 131.0 (C-2′/6′), 131.0 (C-3′/5′), 128.0 (C-4′).
